# Quantifying the role of nitrogen in achieving global Sustainable Development Goals

**DOI:** 10.1038/s41467-026-76777-w

**Published:** 2026-08-15

**Authors:** Yi Zhou, Xiuming Zhang, Yiyang Zou, Chen Wang, Xin Xu, Baojing Gu

**Affiliations:** 1 State Key Laboratory of Soil Pollution Control and Safety, Zhejiang University, Hangzhou, China; 2 College of Environmental and Resource Sciences, Zhejiang University, Hangzhou, China; 3 Policy Simulation Laboratory, Zhejiang University, Hangzhou, China; 4 International Institute for Applied Systems Analysis, Laxenburg, Austria; 5 Institute of Fundamental and Transdisciplinary Research, Zhejiang University, Hangzhou, China

**Keywords:** Environmental sciences, Sustainability, Element cycles

## Abstract

Achieving the 2030 Sustainable Development Goals (SDGs) requires balancing well-being with environmental protection, yet the role of nitrogen across these goals remains poorly understood. Here, we show how dietary nitrogen intake and nitrogen release to air and water are associated with SDG progress across 166 countries from 2000 to 2022. Higher per-capita nitrogen intake is positively associated with social and economic SDGs, whereas nitrogen release is negatively associated with environmental goals. Economic development is linked to improved SDG performance but also to greater dietary nitrogen demand and nitrogen losses, while warming is associated with pressure on food systems and nitrogen management. Nitrogen-related factors account for 38% of the variation in SDG scores, compared with 34% for climatic conditions and 28% for socio-economic factors. These findings identify nitrogen management as a cross-cutting component of sustainable development and support strategies that align food security, pollution control and climate adaptation across development contexts.

## Abstract (web summary)

Balanced nitrogen intake and lower nitrogen releases are linked to progress across the Sustainable Development Goals, helping align human well-being with environmental protection and highlighting the need for context-specific nitrogen management.

## Introduction

In 2015, the United Nations introduced the Sustainable Development Goals (SDGs), a set of 17 interconnected objectives aimed at eradicating poverty, protecting ecosystems, and promoting shared prosperity^1^. However, escalating climate change, resource scarcity, and environmental degradation have made the path toward achieving these goals by 2030 increasingly uncertain, with notable shortfalls persisting across economic prosperity, social inclusion, and environmental sustainability (Supplementary Fig. 1). The complexity and interdependence of these global challenges undermine the effectiveness of isolated policy measures or single-goal strategies^2–4^, highlighting the need to identify systemic drivers that influence progress across multiple SDGs^5–7^. Nitrogen is a critical driver of global sustainability, influencing a broad range of SDGs through both direct and indirect pathways. As an essential nutrient for food production, nitrogen supports the diets of nearly half of the global population, playing a pivotal role in alleviating poverty (SDG 1) and ensuring food security (SDG 2)^8,9^. However, its losses in agriculture and industrial production generate multiple environmental externalities^10^. In 2022, global nitrogen losses reached 142 million tonnes (Tg), more than three times the safe planetary boundary of 43 Tg^11^, exacerbating threats to ecosystem stability (SDGs 14 and 15) and human health (SDGs 3 and 6). Furthermore, emission of nitrous oxide (N_2_O), with a global warming potential approximately 300 times that of carbon dioxide (CO_2_), also leads to global climate change (SDG 13)^12,13^. Thus, sustainable production and consumption (SDG 12) are intrinsically linked to effective nitrogen management to promote its production function while mitigating its environmental and climate impacts^14^. Considering the varying sensitivity of ecosystems and the differing levels of development across countries, regional equity and international cooperation justice are also impacted by nitrogen pollution (SDGs 10, 16, and 17)^15^.

To meet the nutritional needs of the global population without exceeding its safe planetary boundary, it is essential to consider the diversity of agricultural systems and the broader context of non-agricultural nitrogen releases. Agricultural food systems remain the primary drivers of nitrogen cycle disruptions at national, regional, and global scales^16^. Therefore, ensuring the sustainable use of nitrogen necessitates measures that improve nitrogen use efficiency (NUE) while minimizing environmental impacts. Although origin-based solutions may offer more specific and impactful results, policymakers still have a wide range of options available to address nitrogen pollution across the whole food production and consumption chain^17,18^. Integrated management across the whole chain and aligning policies across different environmental domains are critical to avoiding the risk of substituting one form of nitrogen pollution for another^19^. Although the increasing number of studies assessing the relationship between agricultural nitrogen use, its impact on food security, the environment, and nitrogen’s role in sustainable development, most of these studies focus on one or a few SDGs, such as SDG 2 and SDG 12^15,20–22^. Few studies have systematically quantified dietary nitrogen intake and nitrogen losses across the full supply chain across all sectors, or assessed how these metrics relate to progress on the overall SDG index. Their contributions to global sustainability, therefore, remain poorly resolved.

This study addresses this knowledge gap by integrating a global Coupled Human And Natural Systems (CHANS) model with structural equation modeling (SEM) and principal component analysis (PCA). We assess the influence of nitrogen on SDG progress across 166 countries from 2000 to 2022. Specifically, the study (1) examines temporal trends in per capita nitrogen intake from food systems, including both plant based and animal based consumption, and total nitrogen releases, including emissions to air and losses to water, (2) quantifies the effects of nitrogen related drivers on the SDG index, a composite indicator of national performance across the 17 SDGs calculated as the average of individual SDG scores, (3) explores interactions among nitrogen dynamics, socioeconomic factors, and environmental conditions, and (4) evaluates the relative contributions of these drivers to SDG performance. By embedding nitrogen flows within broader socioeconomic and climatic contexts, this study provides a comprehensive assessment of nitrogen’s role in shaping global sustainability. The findings offer insights for improving nitrogen management and for supporting integrated policies aligned with the 2030 Sustainable Development Agenda.

## Results and discussion

### Nitrogen intake and release

Using the Food Balances database from the Food and Agriculture Organization (FAO)^23^, we derived country-level per-capita dietary nitrogen intake. From 2000 to 2022, global dietary nitrogen intake showed a consistent upward trajectory, increasing by approximately 25%, from 4.3 to 5.4 kg of nitrogen per capita per year (Supplementary Fig. 2a). This increase was driven by rising consumption of both plant- and animal-based foods, with animal-derived sources contributing more substantially to the overall growth. Over the period 2000-2022, per capita nitrogen intake from plant-based foods rose by 16%, from 2.7 to 3.1 kg, whereas intake from animal-based foods increased by 40%, from 1.6 to 2.2 kg. This shift reflects broader trends of income growth and dietary transition, particularly in emerging economies where demand for protein-rich diets has accelerated^24^. Since the late 20th century and especially during the early 21st century, the globalization of food supply chains and urbanization have further enhanced access to animal products through improved distribution and affordability^25^.

Nitrogen release estimates are based on CHANS model outputs in this study. In parallel, global per capita nitrogen release declined by approximately 8%, from 20 to 18 kg between 2000 and 2022 (Supplementary Fig. 2b). This study considers four major forms of nitrogen loss: ammonia (NH_3_), nitrogen oxides (NO_x_), nitrous oxide (N_2_O), and nitrate (NO_3_^-^). Emissions to the atmosphere decreased by 11%, and nitrogen losses to water declined by 5%. Among these, global average per capita NO_x_ emissions saw the steepest reduction, falling from 4.9 to 3.8 kg nitrogen, representing a 22% decrease. This achievement is largely attributed to stricter emission standards in the transportation sector, industrial upgrades, investments in clean fuels^26,27^, and the widespread adoption of electric and low-emission vehicles^28,29^. In contrast, reductions in nitrogen losses to water have lagged due to limited improvements in agricultural NUE and the slow modernization of wastewater treatment infrastructure in many regions^30,31^.

**Fig. 1:**
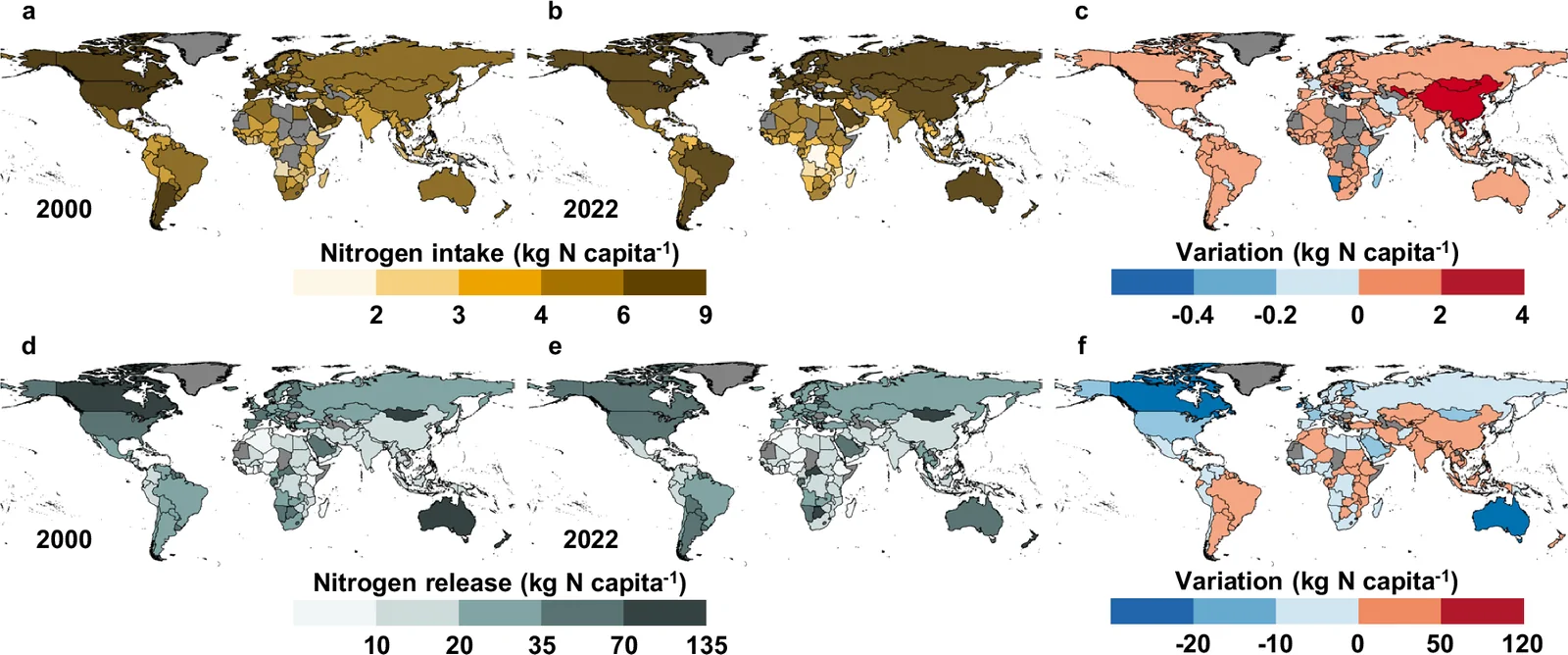
Global patterns and changes in total nitrogen intake and release from 2000 to 2022. **a, b** Spatial distribution of total dietary nitrogen intake in 2000 and 2022, respectively. **c** Change in total dietary nitrogen intake from 2000 to 2022. **d, e** Spatial distribution of total nitrogen release to air and water in 2000 and 2022, respectively. **f** Change in total nitrogen release from 2000 to 2022. Dietary nitrogen intake was calculated from the FAO Food Balances database (https://doi.org/10.4060/cd7162en). Nitrogen release includes NH_3_, NO_x_, and N_2_O emissions to air and NO_3_^-^ losses to water, estimated using the CHANS model. All values are expressed as kg N capita^−1^; changes represent values in 2022 minus values in 2000. Gray areas indicate missing data. The base map with administrative boundaries was created using data from GADM (https://gadm.org/data.html), without endorsement.

Substantial regional disparities emerged in dietary nitrogen patterns. In 2022, China and North America had the highest per capita nitrogen intake, at 7.7 kg and 7.2 kg, respectively, which is approximately 44% and 34% higher than the global average. North America’s nitrogen intake was primarily driven by meat-centric dietary habits, with nitrogen from animal-based foods approximately double that from plant sources, reaching 4.8 kg per capita and accounting for 67% of total nitrogen intake. In contrast, China led globally in per capita plant-based nitrogen intake at 4.6 kg, accounting for 59% of total nitrogen intake, a pattern shaped by traditional grain-based diets dominated by rice and wheat^32^. Similar trends are observed in the Middle East, North Africa, and India, although dietary protein sources remain relatively constrained^33,34^. In 2022, per capita dietary nitrogen intake in Sub-Saharan Africa was 3.5 kg. Of this total, nitrogen from animal-based foods accounted for only 0.7 kg per capita, the lowest among all regions, indicating that food access and nutritional status in this region remain far below the global average. Between 2000 and 2022, China also experienced substantial growth in grain-based nitrogen intake (Supplementary Fig. 3c), reflecting national strategies to improve food security and enhance agricultural self-reliance^35,36^. In Europe and Brazil, animal-based nitrogen intake reached 4.1 and 3.8 kg per capita, respectively, accounting for more than 60% of total dietary nitrogen. These levels are underpinned by extensive livestock production systems, abundant pasturelands, industrialized meat processing, and high consumer demand for animal proteins^37,38^. Overall, nitrogen intake increased only modestly in high-income countries but rose more rapidly in low- and middle-income countries, as classified under the World Bank fiscal year 2024 income classification^39^ (Fig. 1c). In the latter group, rising incomes, urban expansion and improved food accessibility were associated with higher nitrogen consumption^40,41^.

Despite the persistence of global nitrogen pollution, some countries have made notable progress in reducing emissions (Fig. 1f and Supplementary Fig. 4c, f). North America and Europe achieved significant reductions in per capita nitrogen release, with declines of 31% (equivalent to 17 kg of nitrogen per capita) and 24% (7 kg of nitrogen per capita), respectively. These reductions are the result of integrated environmental policies, technological innovation, and regulatory enforcement across agricultural, industrial, and transportation sectors^42^. Yet, nitrogen releases in many high-income countries remain substantial due to persistently high resource demand and production intensity^43^. For example, in 2022, per-capita nitrogen release was 38 kg in North America and 24 kg in Europe, both above the global average. In contrast, nitrogen release has continued to rise in Brazil, China, India, and other parts of Asia. In these regions, the extensive use of synthetic fertilizers, limited adoption of precision agriculture, and weak institutional frameworks for nitrogen management have intensified pollution^44^. Brazil, in particular, experienced a 27% increase in atmospheric nitrogen losses and a 22% rise in nitrogen losses to water, closely linked to agricultural intensification and the expansion of livestock production^45^. Additionally, widespread deforestation and land-use change have increased ecosystem vulnerability, further driving nitrogen losses^46^. These trends underscore the need for improved nitrogen governance in regions undergoing rapid agricultural transformation and ecological disruption.

### Driving pathways

We first discuss nitrogen–SDG associations by world region, because regional groupings capture spatial heterogeneity in dietary structure, agricultural systems, climatic background, and nitrogen-management context. These regional patterns provide the geographical basis for interpreting the country-level associations identified by the regression analysis. Using a fixed-effects regression model (FEM), we estimate the impact and extent of different factors on the SDG index. A significant positive association is observed between nitrogen intake from plant-based foods and the SDG index (Table 1), particularly within the social dimension, which includes SDGs 1, 3, 4, 5, 10 and 16. This relationship is most pronounced in regions such as Other Asia, India, the Middle East and North Africa, and Sub-Saharan Africa (Supplementary Table 4), underscoring the central role of food security in promoting social well-being^47,48^. Compared to animal-based foods, dietary nitrogen from plant-based sources is generally associated with lower environmental burdens and is regarded as one of the most influential strategies for feeding a projected global population of 10 billion by 2050^49^. Diets rich in plant-based foods are also linked to reduced diet-related morbidity^50^.

**Table 1:** Impact of nitrogen-related factors on Sustainable Development Goal (SDG) performance

	SDG index	Economy	Society	Environment
Plant-based nitrogen intake	3.364***	2.606***	5.829***	0.201
(kg nitrogen capita^−1^)	(0.161)	(0.139)	(0.272)	(0.196)
Animal-based nitrogen intake	3.921***	3.397***	5.669***	1.551***
(kg nitrogen capita^−1^)	(0.109)	(0.095)	(0.184)	(0.133)
Nitrogen release to air	−0.049***	−0.056***	−0.012	−0.091***
(kg nitrogen capita^−1^)	(0.009)	(0.008)	(0.015)	(0.011)
Nitrogen release to water	−0.047**	−0.048**	−0.039	−0.101***
(kg nitrogen capita^−1^)	(0.023)	(0.020)	(0.039)	(0.028)
Country	Yes	Yes	Yes	Yes
Year	Yes	Yes	Yes	Yes
N	3455	3455	3455	3455
Adjusted R^2^	0.411	0.403	0.361	0.081

Each column represents a separate regression model. Standard errors (s.e.) are clustered at the country level, with country and year effects controlled in all regression equations. Plant-based nitrogen intake refers to the total mass of nitrogen ingested per capita annually from plant-based food. Animal-based nitrogen intake refers to the total mass of nitrogen ingested per capita annually from animal-based food. Nitrogen release to air refers to the total mass of nitrogen emitted per capita annually into the atmosphere (including NH_3_, NO_x_, and N_2_O). Nitrogen release to water refers to the total mass of nitrogen lost per capita annually into water bodies (including leaching and runoff). Standard errors are reported in parentheses. Statistical significance is denoted as follows: * *P* < 0.1, ** *P* < 0.05, *** *P* < 0.01.

Nitrogen intake from animal-based foods is positively correlated with the overall SDG index, with a stronger correlation observed in the social dimension and the economic dimension (SDGs 2, 6 to 9, 11, 12 and 17) (Supplementary Table 3). This reflects the contribution of animal product consumption to agricultural productivity, food security, and economic development^51^. While livestock production contributes to economic and social development goals, it accounts for one-third of current anthropogenic nitrogen releases, 68% of which are associated with feed production, thereby posing a major sustainability challenge^52^. In China, the correlation between animal-based nitrogen intake and environmental SDGs is negative (Supplementary Table 5), highlighting unsustainable practices in the livestock sector, such as low manure recycling rates and inadequate manure management^53^.

In addition, decoupling of crop and livestock production further contributes to these challenges, exacerbating agriculture-related pollution and reinforcing health and environmental inequalities^54^. These patterns are further corroborated by the negative relationship between nitrogen release and the SDG index. Given that animal-based nitrogen intake is a dominant driver of nitrogen losses across the full agricultural production chain, it significantly contributes to air and water pollution. Nitrogen release, especially in Europe, the Middle East and North Africa, severely hinders progress in the environmental dimension of the SDGs (Supplementary Table 5). In regions with intensive agricultural activity, nitrogen loss to water contributes to eutrophication and ecosystem degradation^55^, further impeding sustainability outcomes (Supplementary Table 2).

**Fig. 2:**
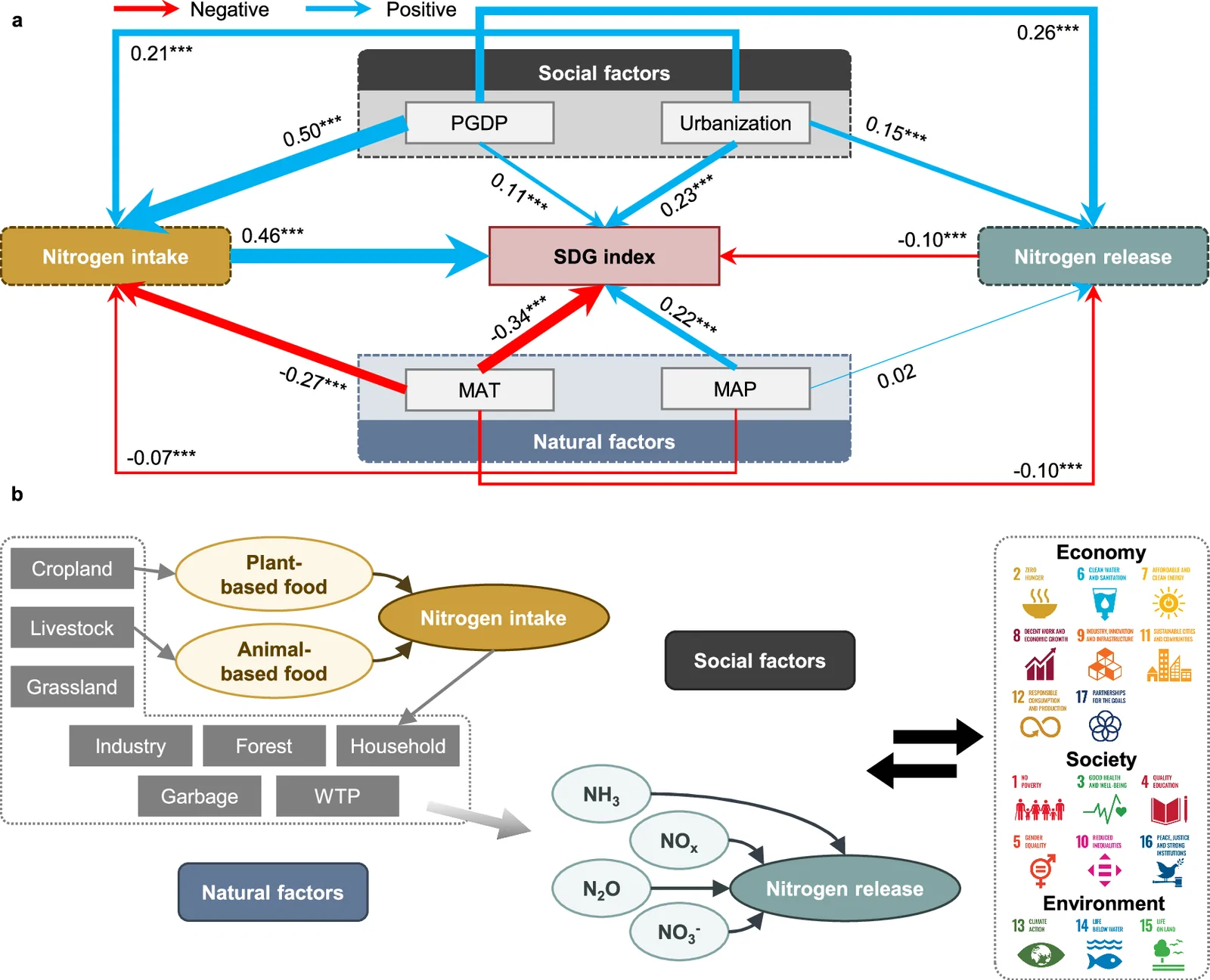
Driving impacts of social, natural, and nitrogen-related factors on the Sustainable Development Goal (SDG) index. **a** The impacts of social, natural, and nitrogen-related factors on the SDG index as deduced through a structural equation model. Asterisks indicate statistical significance: * *P* < 0.1, ** *P* < 0.05, *** *P* < 0.01. Path coefficients represent standardized impacts; red and blue lines indicate negative and positive impacts, respectively, and line width corresponds to impact magnitude. Detailed P values are provided in Supplementary Table 6. PGDP denotes per capita gross domestic product (constant 2021 US$), Urbanization denotes urbanization rate (%), MAT denotes mean annual temperature (°C), and MAP denotes mean annual precipitation (mm). **b** Conceptual model summarizing the selected indicators for social, natural, and nitrogen-related factors and their relationships with the 17 SDGs. WTP denotes wastewater treatment plants. The SDG icons were obtained from the United Nations Sustainable Development Goals website (https://www.un.org/sustainabledevelopment). The content of this publication has not been approved by the United Nations and does not reflect the views of the United Nations or its officials or Member States.

Our SEM analysis (Fig. 2a) reveals the complex multivariate relationships between nitrogen-related factors, socio-economic conditions, environmental factors, and their collective influence on the SDG index. Nitrogen intake from plant-based and animal-based foods shows a significant positive correlation with the SDG index (standardized path coefficient of 0.46). This direct relationship highlights the role of agricultural nitrogen in supporting food production systems, thereby contributing to broader SDG progress^56^. In contrast, nitrogen release shows a direct negative correlation with the SDG index (−0.10), indicating that increasing nitrogen losses are associated with declining SDG progress, particularly within the environmental domain.

Economic growth has a positive direct impact on the SDG index, and its influence is further mediated through nitrogen intake and nitrogen release. The indirect contributions through nitrogen intake and nitrogen release are 0.23 and −0.026, respectively, with the indirect contribution through nitrogen intake accounting for 73% of the total impact. Economic growth, driven by increased resource demand, particularly in food production and industry, leads to higher nitrogen intake. However, higher resource consumption and waste generation also result in increased nitrogen release. While economic growth supports SDG progress, its environmental impacts require mitigation through sustainable production methods^57^. Urbanization also contributes positively to the SDG index (0.23), primarily through improved infrastructure and resource use efficiency with advanced technologies and management. However, urbanization also increases pollution in densely populated areas, amplifying nitrogen release in concentrated urban regions^58^, with a negative impact of 0.015. At the same time, it exerts an indirect positive influence of 0.10 through nitrogen intake, reflecting its role in improving urban residents’ diets and in promoting larger agricultural land areas and farm size, which enhance agricultural productivity^59^.

Natural factors further complicate these relationships. Climate change poses a significant threat to global sustainable development, with a strong negative direct correlation with the SDG index (−0.34). Global warming disrupts climate stability, exacerbates the risk of natural disasters, and threatens food systems^60^. It also affects nitrogen intake, resulting in a reduction of −0.12, consistent with evidence that global warming alone could reduce crop yields by 21%^60^. Additionally, the bidirectional influences of reactive nitrogen on climate cannot be overlooked, with both warming effects from N_2_O emission and cooling effects from NO_x_ and NH_3_ emission through aerosol formation^61^, and the warming influence of nitrogen emission significantly outweighs the regional cooling effect on a global scale^62^.

The impact of precipitation on the SDG index is more complex. Altered precipitation patterns change the form, magnitude, and timing of nitrogen losses, resulting in indirect negative impacts through nitrogen release. On one hand, increased precipitation can intensify nitrogen leaching and runoff, reducing soil nitrogen availability and constraining crop growth, resulting in an indirect impact of −0.03. On the other hand, increased precipitation helps alleviate water scarcity, enhances the performance of water-supply infrastructure, and supports more sustainable water resource management^63^. Studies have found that heavy precipitation events would increase nitrogen loads in U.S. water bodies by about 20% by the end of the century^64^, with future hotspots of agricultural nitrogen loss expected in China, Southeast Asia, East Africa, and Brazil^65^. Therefore, although precipitation poses challenges from an agricultural perspective, its net influence on the SDG index is positive (0.19), as it contributes to progress toward water-related goals.

### Factors contributing to the SDGs

Understanding the key drivers of SDG progress across regions in 2022 is essential for identifying primary relationships among factors and developing context-specific strategies. This understanding requires both spatial and developmental perspectives. The regional analysis in the previous section highlights geographical heterogeneity in nitrogen intake, nitrogen release, and their associations with SDG performance. Here, we further evaluate the relative contributions of nitrogen-related, socio-economic, and natural factors at the national scale and interpret these contributions using both regional patterns and income-group classification. The regional perspective helps identify where nitrogen-related constraints are concentrated, whereas the income-group perspective reflects differences in development stage, infrastructure, governance capacity, fertilizer accessibility, and pollution-control ability.

**Fig. 3:**
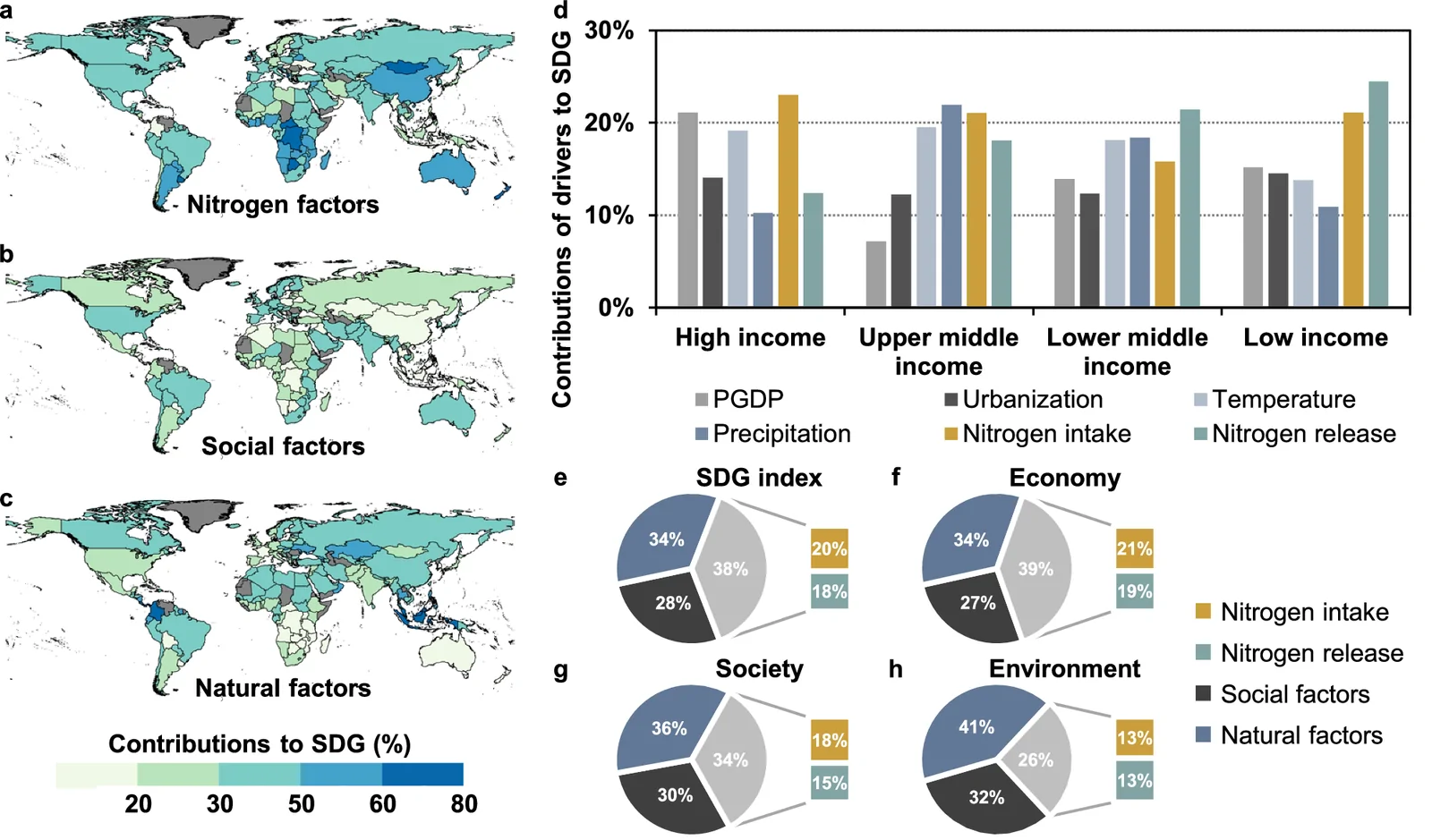
Contribution of nitrogen-related, social, and natural factors to Sustainable Development Goal (SDG) index performance. Relative contributions of nitrogen-related (**a**), social (**b**), and natural (**c**) factors to the national SDG index in 2022 (% of explained variance from PCA regression). **d** Contributions comparison for six key factors (nitrogen intake, nitrogen release, per capita GDP (PGDP), urbanization rate (Urbanization), mean annual temperature (Temperature), and mean annual precipitation (Precipitation)) across income groups (high, upper middle, lower middle, and low income). Income groups follow the World Bank fiscal year 2024 classification based on 2022 gross national income per capita calculated using the Atlas method. Contributions of nitrogen intake, nitrogen release, social factors, and natural factors to overall SDG performance (**e**) and across the three SDG dimensions: Economy (**f**), Society (**g**), and Environment (**h**). Gray areas in maps (**a–c**) indicate missing data. The base map with administrative boundaries was created using data from the GADM (https://gadm.org/data.html), without endorsement.

This study classifies the driving forces into three categories: nitrogen-related factors, including nitrogen intake and release; socio-economic factors, including per capita gross domestic product (GDP) and urbanization rate; and natural factors, including mean annual temperature and precipitation. The PCA was used to evaluate their contributions to SDG index variation (Fig. 3). Globally, nitrogen-related factors emerged as the key driver, accounting for 38% of the variation, followed by natural conditions (34%) and socio-economic factors (28%).

The relative contributions of these drivers vary markedly across countries (Fig. 3a–c). Brazil and India are classified as upper-middle-income and lower-middle-income countries, respectively, under the World Bank fiscal year 2024 income classification^39^. In both countries, the combined contributions of socio-economic, natural and nitrogen-related factors are comparatively large, which may reflect the interaction between development conditions and environmental vulnerability. Notably, the impact of socio-economic factors exceeds the global average in many regions. India, in particular, exhibits a high reliance, with socio-economic conditions contributing 35% to SDG advancement. These findings highlight the importance of economic growth, infrastructure development, and urbanization quality in driving progress. By contrast, in the Former Soviet Union region, natural conditions account for nearly 50% of the contribution. The dominance of cold and subarctic climates renders the region highly sensitive to climate variability^66^, which poses substantial risks to food security, water resources, and ecosystem health, core components of the SDG framework (Supplementary Fig. 5e). In Sub-Saharan Africa, nitrogen-related factors contribute 47% to SDG progress (Supplementary Fig. 5a, b), underscoring the heavy reliance on nitrogen inputs for crop productivity and agricultural sustainability. Limited fertilizer access and weak pollution control infrastructure constrain both nitrogen use and emissions management, which in turn hinders SDG progress^67^. In contrast, Europe and North America display more balanced contributions across the three categories, reflecting a synergistic integration of economic development, environmental stewardship, and nitrogen management.

The income-group perspective provides an additional lens for understanding the regional patterns described above (Fig. 3d). In the SEM analysis, socio-economic variables, particularly GDP and urbanization, show strong associations with both nitrogen intake and nitrogen release, suggesting that the development stage is closely linked to the way nitrogen dynamics interact with SDG performance. This interpretation is also consistent with the contribution analysis across income groups. While the contribution of nitrogen intake remains relatively similar across the four income groups, the contribution of nitrogen release decreases with increasing income level. This pattern suggests that, as countries improve pollution control and reactive nitrogen management, the relative importance of nitrogen release for SDG performance declines, whereas other drivers, especially climate-related constraints, become proportionally more prominent. For example, high-income and upper-middle-income countries have generally made substantial progress toward the SDGs and have more stable economic development, stronger environmental governance, and more advanced nitrogen-loss control. With the Zero Hunger goal largely achieved and nitrogen losses more effectively managed, the potential for further improvements through traditional socio-economic factors is relatively limited^68^. However, because of the widespread, long-term, and transboundary nature of climate risks, global warming has become an increasingly important constraint on sustainable development in these countries, particularly in relation to climate adaptation, energy transition, and international responsibility^69^. By contrast, in low-income countries, nitrogen-related constraints remain dominant, contributing 46%, with both agricultural nitrogen use and dietary nitrogen intake playing central roles, as basic food security and nutrition needs often take precedence over climate adaptation strategies^70^.

Across the three SDG dimensions (Fig. 3e–h), socio-economic factors exert a greater impact on environmental SDGs (32%) compared to economic ones (27%). This contrast may reflect unequal capacities to translate economic development into environmental protection: high-income countries generally have greater financial and technological resources to invest in environmental technologies, whereas low- and lower-middle-income countries may face stronger pressure to prioritize short-term resource use over environmental protection because of financial, technological and external economic constraints^71^. Nitrogen-related drivers play substantial roles in both economic (39%) and social (34%) SDGs, reflecting their centrality to agricultural productivity, human nutrition, and pollution mitigation. These results highlight the importance of integrated nitrogen management in linking environmental sustainability with socio-economic development.

### Promoting the SDGs from a nitrogen perspective

Achieving the SDGs through improved nitrogen management requires strategies that account for both regional heterogeneity and differences in development stage. Regional differences reflect diverse agricultural systems, dietary structures, climatic conditions, and nitrogen-loss pathways, whereas income groups better capture differences in fertilizer accessibility, infrastructure, governance capacity, and pollution-control ability. Therefore, the policy discussion below is structured primarily by income group, while also recognizing the regional contexts in which these nitrogen-management challenges occur.

In low-income countries, where nitrogen intake and release have the most significant impact on SDG progress, improving access to fertilizers remains crucial, as nitrogen management plays a key role in enhancing agricultural productivity. Expanding fertilizer availability and promoting NUE through precision agriculture and education are essential for improving food security and reducing malnutrition^72^. These challenges are also relevant to many lower-middle-income countries, where farmer education and technical support are particularly important for improving NUE and reducing nitrogen losses. In many smallholder systems, fertilizer overapplication is often used as a risk-avoidance strategy to ensure yield security, especially where farmers face limited access to soil testing, technical advice, stable markets, or crop insurance^73^. Therefore, expanding fertilizer availability should be accompanied by extension services, field-based training, soil testing, nutrient budgeting, and site-specific fertilizer recommendations. Additionally, by gaining insights into farmers’ needs and preferences, governments can implement systematic policy interventions to improve fertilizer accessibility^74^. This is a key strategy for enhancing agricultural productivity, increasing farmers’ incomes, and ultimately reducing poverty levels. International support is also vital for addressing infrastructure gaps, while sustainable agricultural practices, such as the recycling of organic resources, can foster both environmental sustainability and poverty alleviation^75^. Although economic growth may present challenges, careful monitoring and regulation of fertilizer use are necessary to prevent unintended environmental damage.

In middle-income countries, where the contributions of various factors to SDG progress are relatively balanced, the impact of nitrogen release is more prominent in lower-middle-income countries. The primary challenge lies in balancing economic growth with environmental sustainability, particularly in regions dependent on intensive agriculture. These countries should focus on modernizing agricultural systems through integrated nutrient management, including the coordinated use of synthetic fertilizers and organic resources where locally appropriate^76^. However, organic amendments are not risk-free, and their outcomes depend on management conditions such as carbon-nitrogen ratio, application rate and timing, soil moisture, and aeration. If poorly managed, some organic amendments may stimulate denitrification and increase greenhouse gas emissions^77^. Therefore, improvements in NUE should also include complementary technologies, such as enhanced-efficiency fertilizers, controlled-release fertilizers, nitrification inhibitors, and crop varieties or rotations with biological nitrification inhibition or biological denitrification inhibition capacity^78^. Implementing pollution control frameworks, such as the European Union’s National Emissions Ceilings Directive^79^, can further mitigate nitrogen-related environmental harm. When designing policies, it is critical to ensure that smallholder farmers are not adversely affected. Providing compensation mechanisms, technical support, and incentives to enhance fertilizer use efficiency is key to protecting vulnerable communities from the negative consequences of nitrogen pollution^80^.

In high-income countries, where nitrogen intake from animal-based sources plays a more prominent role, there is an opportunity to internalize agricultural externalities into food prices. This approach can encourage sustainable farming practices and dietary changes, such as reducing animal protein consumption^81,82^. With access to advanced technologies and financial resources, these regions are well-positioned to enforce stringent nitrogen emission regulations. Investments in advancing NUE technologies, organic farming, and ecological practices can further reduce nitrogen’s environmental footprint^82^. However, it is crucial that policies also support agricultural productivity and farmers’ incomes, ensuring a balance between environmental goals and the economic realities of agriculture.

Effective nitrogen management is central to advancing the SDGs. By improving NUE across the whole chain, mitigating emissions, and addressing the environmental and economic impacts of nitrogen pollution, countries can make significant progress toward achieving these goals^9^. However, managing potential risks and ensuring that policies benefit all sectors, particularly vulnerable communities, is essential. International cooperation and knowledge sharing are crucial to supporting these efforts, making nitrogen management a key component of global sustainability. A balanced approach that addresses environmental, social, and economic needs will contribute to a more sustainable and equitable future for all.

## Methods

### Data sources

We categorized the data into three main categories: nitrogen-related factors, social factors, and natural factors. The data spans from 2000 to 2022, covering a total of 23 years of panel data across 166 countries. The nitrogen intake data used in this study are derived from the Food Balances database of the United Nations Food and Agriculture Organization (FAO, https://doi.org/10.4060/cd7162en)^23^, which provides detailed protein supply records on plant-based and animal-based food consumption categories.

Full-system nitrogen release estimates were derived primarily from the CHANS mass-balance framework^83^. Inputs used to parameterize CHANS were assembled from three main sources. First, national- or regional-level socio-economic and activity data were compiled from global statistical sources (e.g., FAOSTAT, the World Bank (https://data.worldbank.org/), and IFASTAT), covering population and economic activity, land use, agricultural production and management (including cropland area, fertilizer use, crop yields, livestock numbers, and manure management), transport activity, biomass burning, and wastewater treatment. Second, non-agricultural activity data (e.g., fuel consumption) were taken from the International Energy Agency (IEA) World Energy Balances (https://www.iea.org/data-and-statistics/data-product/world-energy-balances). Third, parameters and emission factors were obtained from the recent literature (field experiments and meta-analyses) and national emission inventories, including variables such as crop nitrogen content, harvest indices, manure recycling rates, and region-specific emission factors; where country-specific factors were unavailable, globally documented default values were applied to ensure consistent accounting. The data sources for each subsystem are detailed in Supplementary Method 3.

To support the compilation, harmonization, and cross-checking of sectoral emission information, we drew on several widely used global emission datasets (Supplementary Fig. 6), including the Community Emissions Data System (CEDS, https://www.pnnl.gov/projects/ceds)^84^, the Emissions Database for Global Atmospheric Research (EDGAR, https://edgar.jrc.ec.europa.eu/)^85^, the Integrated Model to Assess the Global Environment (IMAGE, https://www.pbl.nl/en/image/home) framework^86^, and the Model of Agricultural Production and its Impact on the Environment (MAgPIE, https://www.pik-potsdam.de/en/institute/departments/activities/land-use-modelling/magpie)^87^.

Socio-economic variables were obtained from the World Bank database^88^ (https://data.worldbank.org/), from which per capita GDP (measured in 2021 USD equivalents) and urbanization rates for each country are calculated using total GDP and population statistics. For the cross-sectional contribution analysis and policy interpretation, countries were grouped according to the World Bank fiscal year 2024 income classification^39^, which is based on 2022 gross national income per capita calculated using the Atlas method. The four income groups are low-income, lower-middle-income, upper-middle-income and high-income economies (Supplementary Table 1). In this study, middle-income countries refer collectively to lower-middle-income and upper-middle-income countries. Natural-condition variables were derived from the most recent Copernicus Climate Change Service Information 2025 published by the European Union’s Copernicus Earth Observation Programme^89^ (https://climate.copernicus.eu/), which was used to extract and calculate the annual average temperature and annual average precipitation for each country.

### National-scale nitrogen budget

The national-scale nitrogen release data used in this study were derived from the global nitrogen budget constructed by the CHANS model^83^. CHANS is a data-driven mass-balance model that tracks nitrogen inputs, outputs, and accumulation across 14 coupled subsystems: cropland, livestock, grassland, human, industry, aquaculture, forest, pets, urban lawns, solid waste, wastewater, atmosphere, surface water, and groundwater (Supplementary Fig. 7). It resolves multiple reactive nitrogen fluxes, including atmospheric emissions of N_2_O, NO_x_, and NH_3_, and aquatic losses of NO_3_^-^, among others. Annual country-level NH_3_, NO_x_, N_2_O, and NO_3_^-^ fluxes were extracted from the relevant CHANS subsystems for 2000–2022^83,90^. Additional methodological details, including the sector-specific nitrogen budget calculations and a full description of the model framework, are provided in the Supplementary Methods.

The spatial boundary of CHANS covers 166 countries (national scale) in this study, and results are reported as both national totals and per-capita values over 2000–2022 for all core analyses. While the model internally represents 14 subsystems, results are aggregated into eight policy-relevant emitting sectors: cropland, livestock, grassland, forest, household, industry, garbage (solid-waste) treatment, and wastewater treatment plants. External inventories with shorter temporal coverage were used for calibration/validation only. Nitrogen releases from natural ecosystems (e.g., wetlands, oceans, wildlife) are not included in national losses. For the SDG regressions, we use NO_3_^-^ as the water-quality pressure indicator and do not include organic nitrogen exports to water. Finally, to avoid double-counting, dietary nitrogen intake reflects apparent consumption (including net trade), whereas nitrogen releases are reported on a territorial (production-based) basis.

### Statistical analysis

Multiple regression analysis. To assess the impact of nitrogen-related factors on the SDG index, we used multiple regression analysis. The four parameters considered are: per capita plant-based nitrogen intake, per capita animal-based nitrogen intake, per capita nitrogen release to air, and per capita nitrogen release to water. These variables were chosen based on their direct and indirect relationships to both environmental and socio-economic SDGs, and were analyzed using multiple regression with STATA/MP 18.0 software. The fixed-effects regression model is specified in Eq. (1).

1 $${{\rm{SDG\; index}}}=\alpha \cdot {N}_{{{\rm{plant}}}}+\beta \cdot {N}_{{{\rm{animal}}}}+\gamma \cdot {N}_{{{\rm{air}}}}+\delta \cdot {N}_{{{\rm{water}}}}+\varepsilon+{\theta }_{1}+{\theta }_{2}$$

where α, β, γ, and δ represent the coefficients calculated through regression analysis; ε is the constant term; ${\theta }_{1}$, ${\theta }_{2}$ are the error terms. ${N}_{{{\rm{plant}}}}$ represents the nitrogen content in per capita plant-based food intake, ${N}_{{{\rm{animal}}}}$ represents the nitrogen content in per capita animal-based food intake, ${N}_{{{\rm{air}}}}$ represents the nitrogen content in per capita atmospheric nitrogen emissions, and ${N}_{{{\rm{water}}}}$ represents the nitrogen content in per capita nitrogen loss to water.

The model uses panel data from 2000 to 2022, which includes nitrogen intake, nitrogen release, and the SDG index for 166 countries. All nitrogen-related factors are expressed in kg of nitrogen per capita per year. To control for time-invariant national factors (e.g., long-term political policies, governance measures) that could influence nitrogen levels, we included a national-level intercept term (${\theta }_{1}$). Additionally, to account for global temporal trends (e.g., economic crises, political events), we included a year intercept term (${\theta }_{2}$). The objective of this regression analysis is to evaluate the influence of nitrogen-related variables on SDG progress, specifically to quantify the strength and direction of their relationships with the SDG index. This analysis provides insight into how different forms of nitrogen consumption and release contribute to the advancement of the SDGs.

Structural equation modeling. SEM was used to explore the complex relationships between nitrogen-related factors, socio-economic factors, environmental conditions, and the SDG index. SEM is an effective statistical tool for analyzing the interdependencies between multiple variables, including both observed (explicit) and latent (unobserved) variables. It provides a comprehensive understanding of both direct and indirect effects among factors, offering a more nuanced and holistic view of the relationships involved. Standardized path coefficients are used to represent the values derived from the path analysis in SEM^91,92^. These coefficients indicate the strength and direction of relationships between variables in the model. They are standardized, meaning they are scaled within a range from −1 to 1, making it possible to compare different variables regardless of their original units. Path coefficients close to 1 or −1 indicate a strong relationship, while coefficients near 0 suggest a weak or no direct relationship. These coefficients help clearly identify the magnitude and direction of each relationship in the model, enabling comparisons of different paths within the model and providing insight into the overall impact of one variable on another. This makes them a key tool for assessing the contributions of various independent variables to the dependent variable and for understanding the structure of the correlation network.

While SEM can be used to test causal relationships, it is important to clarify that this study focuses on correlations rather than directly establishing causal pathways between variables. The goal of using SEM in this study was to model the multivariate relationships between nitrogen-related factors, social and environmental factors, and their collective impact on SDG progress. We aimed to understand both the direct and indirect associations of nitrogen on the SDGs, as well as the influence of socio-economic and environmental factors on nitrogen-related variables, to ultimately present their direct, indirect, and net effects, with a particular focus on how socio-economic and environmental factors mediate these relationships.

First, we identified potential interactions among the key variables based on the theoretical framework of nitrogen’s role in driving SDG outcomes, incorporating prior empirical knowledge on nitrogen’s impact on agricultural productivity, food security, and environmental sustainability. After selecting the key variables, we used statistical tests to evaluate the significance of each variable’s contribution to the model. Variables with insignificant contributions were progressively removed, and we compared the full model with the simplified version using goodness-of-fit statistics.

To assess the model fit, we employed three widely accepted indices: SRMR (Standardized Root Mean Square Residual), with a value less than 0.08 indicating a good fit; RMSEA (Root Mean Square Error of Approximation), with a value less than 0.1 indicating a good fit; and the Chi-square test, where a P-value greater than 0.05 indicates a good fit. We selected the optimal structural equation model based on the Comparative Fit Index (CFI), where a higher CFI value indicates a better fit between the observed data and the theoretical model. The data inputs for SEM included the SDG index, nitrogen-related factors, socio-economic variables (such as GDP and urbanization rates), and environmental factors (such as temperature and precipitation), all of which were standardized and temporally aligned to ensure consistency across the model. Finally, Fig. 2, Supplementary Tables 6 and 7 present the direct and indirect impacts of all factors in the model, along with the standardized path coefficients, providing a detailed explanation of the roles and interrelationships of the variables within the model.

The multiple regression and SEM analyses in this study provide a comprehensive approach to understanding the relationships between nitrogen-related factors and SDG progress. The methods used allow for the evaluation of both direct and indirect impacts, offering valuable insights into the roles of different factors in the SDGs.

### Nitrogen contribution quantification

We employed PCA to investigate the spatial differences in driving factors across various countries. Prior to conducting the driving factor analysis, a preliminary correlation analysis and least squares test were performed on all relevant variables in our database and the SDG data from the Sustainable Development Report. Subsequently, variables that exhibited significant correlations were selected for driving factor analysis. Based on their characteristics, the identified driving factors were classified consistently with the factors applied in the aforementioned SEM: nitrogen-related factors (per capita nitrogen intake from plant-based food and animal-based food, per capita nitrogen release to air and water), social factors (per capita GDP, urbanization rate), and natural factors (temperature, precipitation). To address the interactions between predictor variables, we used PCA and principal component regression (PCR) to assess the relative importance of each driving factor at the national level. Specifically, through PCA, we transformed the input variables (standardized to standard deviations) into a set of new uncorrelated variables, principal components, which still accounted for all the variability in the data. Each principal component was associated with its loading values, indicating its correlation with the input variables. We then performed multiple linear regression with the SDG index as the dependent variable and all principal components as independent variables. The score for each variable was defined as the sum of the absolute values of the PCR regression slope multiplied by the corresponding PCA loading values. Finally, we calculated the relative importance of the potential driving variables for the SDG index at the national scale. The PCA and PCR procedures used in this study are specified in Eqs. (2) and (3).

2 $${{{\rm{Comp}}}}_{i}=	{\alpha }_{i}\cdot {N}_{{{\rm{plant}}}}+{\beta }_{i}\cdot {N}_{{{\rm{animal}}}}+{\gamma }_{i}\cdot {N}_{{{\rm{air}}}}+{\delta }_{i}\cdot {N}_{{{\rm{water}}}}+{\varepsilon }_{i}\cdot {{\rm{PGDP}}}+{\theta }_{i}\cdot {{\rm{Urban}}} \\ 	+{\mu }_{i}\cdot {{\rm{MAT}}}+{\pi }_{i}\cdot {{\rm{MAP}}}+\rho$$

3 $${{\rm{SDG\; index}}}={\sum }_{i=1}^{5}{\sigma }_{i}\cdot {{{\rm{Comp}}}}_{i}+\tau$$

where ${{{\rm{Comp}}}}_{i}$ represents the ${{\rm{I}}}$ th principal component, with five principal components selected for analysis. α, β, γ, δ, ε, θ, μ, and π are the loading values of each principal component, obtained through PCA. ρ, σ, and τ are the coefficients to be estimated. ${{\rm{PGDP}}}$ represents per capita GDP (measured in 2021 US dollar equivalents), ${{\rm{Urban}}}$ represents the urbanization rate, and ${{\rm{MAT}}}$ and ${{\rm{MAP}}}$ represent the mean annual temperature and mean annual precipitation, respectively. We first calculated the score for each driving factor for the SDG index, and then computed the total scores for the three categories of driving factors. The relative contribution of each driving factor to the SDG index was calculated as the category score divided by the total score. The scores and relative contributions of nitrogen-related factors, for example, are defined in Eqs. (4) and (5).

4 $${{{\rm{Score}}}}_{{{\rm{nitrogen}}}}=	{{{\rm{Score}}}}_{{N}_{{{\rm{plant}}}}}+{{{\rm{Score}}}}_{{N}_{{{\rm{animal}}}}}+{{{\rm{Score}}}}_{{N}_{{{\rm{air}}}}}+{{{\rm{Score}}}}_{{N}_{{{\rm{water}}}}} \\=	{\sum }_{i=5}^{5}\left|{\sigma }_{i}\cdot {\alpha }_{i}\right|+{\sum }_{i=5}^{5}\left|{\sigma }_{i}\cdot {\beta }_{i}\right|+{\sum }_{i=5}^{5}\left|{\sigma }_{i}\cdot {\gamma }_{i}\right|+{\sum }_{i=5}^{5}\left|{\sigma }_{i}\cdot {\delta }_{i}\right|$$

5 $${{{\rm{Con}}}}_{{{\rm{nitrogen}}}}\left(\%\right)=\frac{{{{\rm{Score}}}}_{{{\rm{nitrogen}}}}}{{{{\rm{Score}}}}_{{{\rm{total}}}}}\times 100$$

where ${{{\rm{Score}}}}_{{N}_{{{\rm{plant}}}}}$, ${{{\rm{Score}}}}_{{N}_{{{\rm{animal}}}}}$, ${{{\rm{Score}}}}_{{N}_{{{\rm{air}}}}}$, and ${{{\rm{Score}}}}_{{N}_{{{\rm{water}}}}}$ represent the scores for per capita nitrogen intake from plant-based and animal-based food, per capita nitrogen release to air and water, respectively. ${{{\rm{Score}}}}_{{{\rm{nitrogen}}}}$ represents the score for nitrogen-related factors, and ${{{\rm{Score}}}}_{{{\rm{total}}}}$ represents the total score of all eight driving factors. ${{{\rm{Con}}}}_{{{\rm{nitrogen}}}}$ represents the contribution of nitrogen-related factors to the SDG index.

### Uncertainty and limitations

In our analysis, the primary sources of uncertainty stem from variations in nitrogen intake and releases. The nitrogen intake data are derived from official FAO statistics, which are widely considered to be the most reliable global data in this field. In contrast, nitrogen release data is based on activity data and emission factors from the global CHANS model. The data sources and calculation procedures for each CHANS subsystem are provided in the Supplementary Methods.

The limitations of our study primarily arise from several factors. First, the drivers considered in the analysis are selective in nature. The nitrogen intake and release parameters used in this study do not represent all possible forms of nitrogen presence, as the scope of inputs and outputs is limited. Additionally, the range of selected natural and social factors is also somewhat constrained. We selected GDP, urbanization, MAT, and MAP to ensure that the indicators are relatively independent yet complementary, thereby maintaining analytical parsimony, improving comparability, and minimizing interference from highly overlapping drivers. Alternative indicators (e.g., education attainment) could disproportionately inflate their apparent contributions to specific goals (e.g., SDG 4), whereas other dimensions, such as energy systems, natural resource use, and international trade, were not explicitly considered under the same selection principle. However, the primary objective of this study is to explore the role of key nitrogen parameters in driving SDG outcomes and to investigate the implications of the findings. This approach justifies the specific selection of factors.

Second, the study covers the period from 2000 to 2022, and does not account for the impacts of major global disruption events that occurred after this period. These events include the post-pandemic economic transformation, the intensification of geopolitical conflicts, and adjustments to environmental policies across various countries. All of these factors may have affected global nitrogen flow patterns, agricultural and industrial nitrogen management strategies, and pathways to achieving sustainability goals. Therefore, the conclusions drawn from historical data may have limited applicability in current and future contexts. However, this focus allows for a more focused examination of the role and contribution of nitrogen, excluding the complex influence of international politics. In the face of rapidly changing policy environments and global cooperation frameworks, the relevant conclusions and recommendations should be carefully reassessed and adjusted in light of emerging dynamics.

Moreover, this study does not systematically examine the interactions and cumulative effects (cocktail effect) between nitrogen and other key biogeochemical elements such as phosphorus and carbon. While this single-element perspective may not fully capture the complexity of system behaviors under multiple environmental pressures, the coupled effects of nitrogen with phosphorus and carbon have been partially reflected in nitrogen management, as seen in the close coupling of nitrogen and phosphorus in fertilizers. In evaluating SDG pathways, the selected natural and social factors reflect the climatic and economic development conditions of different regions, but they do not comprehensively account for the interdependencies and synergistic effects between factors such as climate change, economic inequality, and resource depletion. In the future, we would expand the study to include more nitrogen management strategies and their broader impacts on SDGs, constructing a multi-element coupling analysis framework to more accurately reveal the systemic and holistic characteristics of sustainability challenges. Additionally, we will focus on exploring the significant roles of other elements, such as carbon and phosphorus, in driving macro-level sustainability and more fully integrate cross-element interactions, global disruption factors, and their cumulative effects on sustainable transitions.

## Supplementary information

Supplementary Information

Transparent Peer Review file

## Source data

Source Data

## Data Availability

The processed country-level datasets generated in this study, including the data underlying the figures and tables, have been deposited in Zenodo at https://doi.org/10.5281/zenodo.17983565^93^. Source data are provided with this paper. The publicly available input datasets used in this study are described in the Methods and Supplementary Information and can be accessed through the original sources cited therein. Source data are provided with this paper.

## Code availability

No custom code or software was developed for this study; all analyses were performed using standard statistical software and publicly documented methods described in the Methods section.
