# Supplementary material for: Quantifying the role of nitrogen in achieving global Sustainable Development Goals
Source: Nat Commun. 2026 Aug 15;17:9818. doi: 10.1038/s41467-026-76777-w (PMC13575186; doi:10.1038/s41467-026-76777-w)
Supplement: Supplementary file 1 [file 41467_2026_76777_MOESM1_ESM.pdf]

Supplementary Information

Quantifying the role of nitrogen in achieving global Sustainable Development Goals

Yi Zhou[1,2,3], Xiuming Zhang[4], Yiyang Zou[1,2], Chen Wang[1,2,3], Xin Xu[1,2], Baojing Gu[1,2,5*]

[1]State Key Laboratory of Soil Pollution Control and Safety, Zhejiang University, Hangzhou, 310058, China.
[2]College of Environmental and Resource Sciences, Zhejiang University, Hangzhou, 310058, China.
[3]Policy Simulation Laboratory, Zhejiang University, Hangzhou, 310058, China.
[4]International Institute for Applied Systems Analysis, Schlossplatz 1, A-2361, Laxenburg, Austria.
[5]Institute of Fundamental and Transdisciplinary Research, Zhejiang University, Hangzhou, 310058, China.

[*]Corresponding Author: bjgu@zju.edu.cn (B.G.)

List of Supplementary Items

Supplementary Methods 3

Supplementary Method 1 | System boundary and internal nitrogen cascade in the CHANS framework. 3

Supplementary Method 2 | Functional groups in nitrogen cycling. 3

Supplementary Method 3 | Nitrogen budget on a global scale and data sources. 4

Supplementary Method 4 | Uncertainty analysis. 10

Supplementary Tables 12

Supplementary Table 1 | Region classifications and income definitions in this study. 12

Supplementary Table 2 | Impact of nitrogen-related factors on SDG index in different regions. 19

Supplementary Table 3 | Impact of nitrogen-related factors on Economy performance in different regions. 20

Supplementary Table 4 | Impact of nitrogen-related factors on Society performance in different regions. 21

Supplementary Table 5 | Impact of nitrogen-related factors on Environment performance in different regions. 22

Supplementary Table 6 | Standardized path coefficients and *P*-values in structural equation model. 23

Supplementary Table 7 | Structural equation model fitting index. 24

Supplementary Figures 25

Supplementary Fig. 1 | Trends of SDG index and performance in "Economy," "Society," and "Environment" from 2000 to 2022. 25

Supplementary Fig. 2 | Historical trends of nitrogen intake and release. 26

Supplementary Fig. 3 | Global patterns and changes in dietary nitrogen intake from plant- and animal-based foods from 2000 to 2022. 27

Supplementary Fig. 4 | Global patterns and changes in nitrogen release to air and water from 2000 to 2022. 28

Supplementary Fig. 5 | Spatial distribution of nitrogen-related, social, and natural factors' contributions to the SDG index in 2022. 29

Supplementary Fig. 6 | Comparison of nitrogen release to air and water from the CHANS model with other models and literature. 30

Supplementary Fig. 7 | Nitrogen dynamics in the Coupled Human and Natural Systems (CHANS) model. 31

Supplementary References: 32

Supplementary Methods

Supplementary Method 1 | System boundary and internal nitrogen cascade in the CHANS framework.

The Coupled Human and Natural Systems (CHANS) model provides a robust framework for examining nitrogen (N) dynamics across interconnected human and natural environments. In this study, we used the global extension of CHANS to quantify nitrogen cycling within a harmonized system boundary for 166 countries over 2000-2022. The framework applies a process-driven, mass-balance approach to track nitrogen inputs, internal transfers, transformations, and releases across eight policy-relevant subsystems: cropland, livestock, industry, household, wastewater treatment plants (WTP), garbage (solid waste), forest, and grassland. The model integrates multiple data streams, including global statistical datasets, experimental and meta-analytical evidence, pollutant inventories, and customized emission factors (EFs), to represent key nitrogen pathways and associated environmental pressures.

Within this boundary, internal nitrogen dynamics are explicitly represented as a cascade of transfers and transformations linking production, consumption, treatment, and life-support compartments. Nitrogen enters the system through inputs such as biological fixation and synthetic fertilizer, circulates through production subsystems (cropland, livestock, and industry), and is transferred to the consumption subsystem (households) via food and goods chains. Nitrogen subsequently moves through treatment subsystems (WTP and garbage), before being released to life-support systems (atmosphere and hydrosphere).

The model accounts for major reactive nitrogen flows, including (i) volatilization and deposition, whereby NHx and NOy emitted from agricultural activities, waste handling, and selected industrial uses are deposited back onto receiving land subsystems (cropland, grassland, and forest) using established country-level deposition datasets (Supplementary Method 3); (ii) soil nitrification–denitrification processes in cropland and grassland that transform reactive N into N_2O, N_2, and NO_3^-, with gaseous emissions and hydrological losses quantified and balances closed using NUE and N surplus; (iii) a hydrological cascade in which NO_3^- leaching and runoff from soils and manure-affected land enter surface- and groundwater pathways, while domestic and industrial wastewater N is routed through WTPs where nitrification/denitrification generate N_2O/N_2 and residual N is discharged to water or transferred via sludge to solid-waste systems; and (iv) recycling loops (e.g., straw return, manure application, and human excreta reuse) that return reactive N to croplands, alongside transfers of harvested crop N to livestock and households (dietary intake), with excretion partitioned between fields and WTPs depending on sanitation coverage. Terminal sinks and storage are also represented, with N_2 treated as an endpoint and biomass/soil accumulation tracked as temporary storage, particularly in forests.

To ensure internal consistency, we integrated multi-source datasets (e.g., FAOSTAT, the World Bank, region-specific emission factors) to inform the CHANS framework. Model outputs (nitrogen fluxes) were standardized and temporally aligned with the SDG dataset from the Sustainable Development Report. The resulting integrated dataset was assembled into a country-year matrix, which served as the core input for the SEM and PCA analyses described in the Methods.

Supplementary Method 2 | Functional groups in nitrogen cycling.

1) Nitrogen processors: These systems include cropland, forest, grassland, and industry. They are responsible for the conversion of nitrogen inputs into usable forms that are essential for biomass production and food generation. Their biogeochemical processes are pivotal to the nitrogen cycle and serve as a foundation for nitrogen transfer across various subsystems. 2) Nitrogen consumers: The household subsystem primarily embodies the nitrogen consumers. Through dietary consumption, nitrogen is integrated into human systems, reflecting the direct role of organisms in the nitrogen cycle and influencing nitrogen cycling dynamics. 3) Nitrogen removers: WTP and waste management systems play an essential role in reducing the environmental impact of excess nitrogen. By managing reactive nitrogen, these systems help mitigate pollution and recycle nitrogen in forms that can be safely handled or returned to the system. 4) Life support systems: The atmosphere, surface water, and groundwater form the core life support systems, facilitating processes such as atmospheric nitrogen fixation and the transformation of nitrogen in aquatic environments. These systems are vital for sustaining nitrogen cycling across the globe. N fluxes among these systems are derived from the input-output process of the above-mentioned eight subsystems.

Through the CHANS framework, this model enables a comprehensive nitrogen budget analysis and illuminates the mechanisms driving nitrogen dynamics across interlinked human and natural systems. By tracking nitrogen flows within different environmental sectors, the model identifies key points where interventions can reduce nitrogen pollution. This systems-based approach is indispensable for understanding the complex interactions within the nitrogen cycle and for developing strategies to manage nitrogen's environmental impacts.

Supplementary Method 3 | Nitrogen budget on a global scale and data sources.

The CHANS model relies on several key data sources to provide an accurate and thorough understanding of nitrogen dynamics. These include: 1) Global statistical databases: These databases supply vital socio-economic and agricultural data at both national and regional levels. Information such as human population statistics, gross domestic product (GDP), land use patterns, crop and livestock production rates, agricultural efficiency, and nitrogen fertilizer usage are included. 2) Recent field research, meta-analyses, and national pollution inventories: Published studies and meta-analyses, along with national inventories of nitrogen emissions, provide essential parameters. These datasets include nitrogen content in agricultural products, crop harvest indices, and manure recycling ratios, offering a detailed look at the nitrogen cycle in agricultural and industrial contexts. 3) Country-specific EFs: These factors are tailored to specific national conditions and are crucial for assessing nitrogen release from various sources. They help refine the model by providing more accurate estimates of nitrogen flows in different regions. The following sections provide a detailed explanation of the structure and computational methodologies of the CHANS model.

Cropland nitrogen dynamics

The nitrogen input in croplands of country i (denoted as $NC_{\text{input},i}$) consists of seven primary elements: nitrogen fertilizer ($NC_{\text{fer},i}$), manure nitrogen ($NC_{\text{man},i}$), biological nitrogen fixation ($NC_{\text{fix},i}$), nitrogen deposition from the atmosphere ($NC_{\text{dep},i}$), irrigation ($NC_{\text{irr},i}$), human excretion ($NC_{\text{hum},i}$), and straw recycling ($NC_{\text{recycle},i}$). On the other hand, nitrogen output in croplands ($NC_{\text{output},i}$) includes four main components: harvested crop nitrogen ($NC_{\text{harvest},i}$), nitrogen gas emissions ($NC_{\text{gas},i}$, covering ammonia (NH_3), nitrogen oxides (NO_x), and nitrous oxide (N_2O)), nitrogen leaching and runoff ($NC_{\text{leaching\&runoff},i}$), and straw recycling ($NC_{\text{recycle},i}$). The nitrogen use efficiency (NUE) in croplands of country i (denoted as

NUE_i) is computed as the ratio of nitrogen in the harvested crop ($NC_{\text{harvest},\,i}$) to the total nitrogen input ($NC_{\text{input},i}$). The nitrogen cycle in croplands can be expressed in supplementary equations (1)-(4).

Inputs:

$$NC_{\text{input},i} = NC_{\text{fer},i} + NC_{\text{man},i} + NC_{\text{fix},i} + NC_{\text{dep},i} + NC_{\text{irr},\,i} + NC_{\text{hum},i} + NC_{\text{recycle},i} \tag{1}$$

Outputs:

$$NC_{\text{output},i} = NC_{\text{harvest},i} + NC_{\text{gas},i} + NC_{\text{leaching\&runoff},i} + NC_{\text{recycle},i} \tag{2}$$

Surplus:

$$NC_{\text{surplus},\,i} = NC_{\text{input},\,i} - NC_{\text{harvest},i} = NC_{\text{gas},i} + NC_{\text{leaching\&runoff},i} + NC_{\text{acc},i} + NC_{\text{recycle},i} \tag{3}$$

NUE:

$$\mathrm{NUE}_i = NC_{\text{harvest},\,i}/NC_{\text{input},i} \tag{4}$$

The components that make up agricultural nitrogen are assessed using various data sources. Nitrogen from fertilizers is obtained from FAO datasets, adjusted to reflect field application rates based on research by Lassaletta[1] and the International Fertilizer Association[2]. Manure nitrogen is derived from livestock application data, including deposition figures available through FAO manure datasets. Biological nitrogen fixation (BNF) is estimated according to crop-specific BNF rates[3]. The nitrogen input from irrigation is calculated by multiplying the irrigation volume by nitrogen concentration. Atmospheric deposition data comes from FAO nitrogen deposition statistics. Nitrogen from human excretion refers to nitrogen that is directly excreted onto fields. Straw recycling represents the nitrogen contributed by crop residues returned to the soil. For nitrogen outputs, crop harvest nitrogen is determined based on crop yield and nitrogen content, using agricultural production data. Emissions of gases such as NH_3, N_2O, and NO_x are based on emission inventories like IMAGE[4] and MAgPIE[5]. Leaching and runoff are estimated using country-specific leaching factors that describe nitrogen loss through water runoff and leaching. Together, these data sources provide a comprehensive view of nitrogen dynamics in agricultural systems, facilitating an in-depth understanding of nitrogen efficiency and its environmental consequences.

Livestock nitrogen dynamics

The livestock subsystem encompasses a complex nitrogen cycle, consisting of various input and output components. Nitrogen inputs ($NL_{\text{input},i}$) in country i are categorized into feed types: protein-based feed ($NL_{\text{pro},i}$), human-edible feed ($NL_{\text{hum},i}$), and soybean-based feed ($NL_{\text{soy},i}$). The nitrogen outputs ($NL_{\text{output},i}$) in this subsystem are divided into three primary categories: animal products ($NL_{\text{ani},i}$), manure ($NL_{\text{man},i}$), and gas emissions ($NL_{\text{gas},i}$). Additional outputs include nitrogen leaching and runoff ($NL_{\text{leaching\&runoff},i}$). Nitrogen surplus ($NL_{\text{surplus},\,i}$), which is unaccounted for in the outputs, represents nitrogen that could potentially lead to environmental impact, and serves as a critical metric for evaluating NUE in livestock systems. The nitrogen cycle in livestock systems can be expressed in supplementary equations (5)-(7).

Inputs:

$$NL_{\text{input},i} = NL_{\text{pro},i} + NL_{\text{hum},i} + NL_{\text{soy},i} \tag{5}$$

Outputs:

$$NL_{\text{output},i} = NL_{\text{ani},i} + NL_{\text{man},i} + NL_{\text{gas},i} + NL_{\text{leaching\&runoff},i} \tag{6}$$

Surplus:

$$NL_{\text{surplus},\,i} = NL_{\text{input},\,i} - NL_{\text{ani},i} - NL_{\text{man},i} = NL_{\text{gas},i} + NL_{\text{leaching\&runoff},i} \tag{7}$$

The nitrogen input and output data for the livestock subsystem are derived from diverse and detailed sources. Feed nitrogen is estimated based on the output of livestock products, which are calculated using

production data for meat, milk, and eggs. The nitrogen content in animal products is referenced from agricultural statistics and scientific literature[6-8]. Manure nitrogen outputs are determined using livestock population data and manure production rates, which can be obtained from agricultural census data and manure management practices. Gas emissions, such as NH_3, N_2O, and NO_x, are sourced from various databases and studies[9-11], including emission factors for manure storage and field application. Leaching and runoff are calculated based on manure contributions to water systems, with leaching typically estimated to be 20% of the runoff, unless specific local data is available. Manure management practices—such as applying manure to cropland or leaving it in pastures—affect the nitrogen fluxes in both cropland and grassland systems, and these practices are informed by agricultural extension services and environmental monitoring initiatives. This comprehensive data collection allows for an accurate representation of the nitrogen cycle in livestock systems, contributing to a better understanding of nitrogen dynamics and environmental impacts.

Grassland nitrogen dynamics

Nitrogen inputs into grasslands in country i (represented by $NG_{\text{input},i}$) include both natural and anthropogenic sources. These inputs consist of fertilizer nitrogen ($NG_{\text{fer},i}$), manure nitrogen ($NG_{\text{man},i}$), nitrogen fixation by grasslands ($NG_{\text{fix},i}$), and atmospheric nitrogen deposition ($NG_{\text{dep},i}$). Irrigation nitrogen ($NG_{\text{irr},i}$) is typically minimal in natural grasslands and is excluded in most regions unless specific conditions indicate its importance. On the output side, nitrogen in grasslands ($NG_{\text{output},i}$) is involved in forage nitrogen ($NG_{\text{for},i}$), gas emissions ($NG_{\text{gas},i}$) including volatilization and denitrification of N_2, and nitrogen losses through leaching and runoff ($NG_{\text{leaching\&runoff},\,i}$). The nitrogen surplus, defined as the difference between inputs and outputs, is a crucial indicator of nitrogen inefficiencies or accumulation within grassland ecosystems. The nitrogen balance within grassland ecosystems can be expressed in supplementary equations (8)-(10).

Inputs:

$$NG_{\text{input},i} = NG_{\text{fer},i} + NG_{\text{man},i} + NG_{\text{fix},i} + NG_{\text{dep},i} + NG_{\text{irr},i} \tag{8}$$

Outputs:

$$NG_{\text{output},i} = NG_{\text{for},i} + NG_{\text{gas},i} + NG_{\text{leaching\&runoff},\,i} \tag{9}$$

Surplus:

$$NG_{\text{surplus},\,i} = NG_{\text{input},i} - NG_{\text{for},i} = NG_{\text{gas},i} + NG_{\text{leaching\&runoff},\,i} \tag{10}$$

To estimate nitrogen inputs and outputs in grasslands, various datasets and models are employed. Fertilizer nitrogen data is derived from national agricultural statistics, with specific allocations for grassland areas based on historical distribution patterns from studies by Lassaletta[12] and the International Fertilizer Association[2]. Manure nitrogen, which accounts for both pasture deposition and applied manure, is sourced from FAO manure datasets, assuming that the application rates on grasslands are similar to those used for croplands[13]. Nitrogen fixation in grasslands is calculated based on grassland-specific biological fixation rates from studies like Davies-Barnard's work[14], which considers both symbiotic and free-living nitrogen fixers. Atmospheric nitrogen deposition data is obtained from modeling efforts like MAgPIE[5], which are tailored to grassland ecosystems and adjusted for regional variability in climate and deposition levels. Irrigation nitrogen is generally considered insignificant unless regional data suggests otherwise. For outputs, forage nitrogen is computed by determining the nitrogen content of harvested forage or grazed materials using yield data and nutrient removal coefficients. Nitrogen gas emissions, encompassing NH_3, N_2O, NO_x,

and N_2, are estimated from emission inventories and IPCC guidelines[15-17], focusing on nitrogen released through volatilization and denitrification processes. Leaching and runoff nitrogen losses are modeled using regional emission factors, supplemented by hydrological and climatic data, to accurately estimate nitrogen fluxes into surrounding water systems[18]. These diverse data sources provide a comprehensive understanding of nitrogen dynamics in grassland systems, allowing for a detailed analysis of nitrogen efficiency and its environmental consequences.

Forest nitrogen dynamics

Nitrogen inputs to forests in country i (denoted as $NF_{\text{input},i}$) consist of three primary components: fertilizer nitrogen ($NF_{\text{fer},i}$), which is applied exclusively to plantation forests; forest biological nitrogen fixation ($NF_{\text{fix},i}$), reflecting nitrogen fixation specific to various forest types; and atmospheric nitrogen deposition ($NF_{\text{dep},i}$), which quantifies nitrogen deposited onto forested areas from the atmosphere. These inputs are essential in maintaining forest nitrogen cycling and contribute to forest nitrogen storage. On the output side, nitrogen from forests in country i ($NF_{\text{output},i}$) is released through several pathways: harvest nitrogen ($NF_{\text{harvest},i}$), which corresponds to the nitrogen content in harvested timber and other forest products; biomass and soil nitrogen accumulation ($NF_{\text{bio},i}$), capturing nitrogen stored in growing forests' biomass and soils; nitrogen gas emissions ($NF_{\text{gas},i}$), which include NH_3, N_2O, NO_x, and other gases released from processes like biomass burning and denitrification; and nitrogen leaching and runoff ($NF_{\text{leaching\&runoff},\,i}$), representing nitrogen loss through precipitation and surface runoff. The nitrogen surplus is calculated as the difference between nitrogen inputs and outputs, with nitrogen loss through gas emissions and leaching considered as the key contributors. The nitrogen cycle in forest ecosystems can be expressed in supplementary equations (11)-(13).

Inputs:

$$NF_{\text{input},i} = NF_{\text{fer},i} + NF_{\text{fix},i} + NF_{\text{dep},i} \tag{11}$$

Outputs:

$$NF_{\text{output},i} = NF_{\text{harvest},i} + NF_{\text{bio},i} + NF_{\text{gas},i} + NF_{\text{leaching\&runoff},\,i} \tag{12}$$

Surplus:

$$NF_{\text{surplus},\,i} = NF_{\text{gas},i} + NF_{\text{leaching\&runoff},\,i} \tag{13}$$

Data on fertilizer application rates for plantation forests are derived from national agricultural statistics, typically assuming an average rate of 150 kg/ha for plantation areas[19, 20]. Forest BNF calculations are based on nitrogen fixation rates specific to forest ecosystems, which can vary significantly depending on tree species and environmental conditions[21]. These rates are informed by studies on ecosystem-specific nitrogen fixation, such as those focusing on symbiotic relationships in forest understories and free-living nitrogen fixers. Atmospheric nitrogen deposition is determined using FAO database information and adjusted for regional forest cover and climate conditions[22], which influence the deposition of nitrogen compounds. The nitrogen content of timber and forest products is derived from forest production statistics, including data from the FAO and regional forest product reports[23]. Nitrogen stored in biomass and soils is estimated using carbon-nitrogen ratio studies and data on forest carbon sequestration, incorporating IPCC guidelines. Emission inventories provide estimates for nitrogen emissions associated with forest management, biomass burning, and soil denitrification processes[24-26]. For nitrogen leaching and runoff, regional hydrological models are applied, incorporating soil and precipitation data to determine nitrogen fluxes into adjacent water bodies. These varied data sources offer a robust framework for evaluating nitrogen cycling in forest ecosystems, helping assess nitrogen efficiency and environmental impact.

Household nitrogen dynamics

In the household subsystem in country i, nitrogen inputs ($NH_{\text{input},i}$) mainly come from three primary sources: food supply ($NH_{\text{food},i}$), industrial products ($NH_{\text{indu},i}$), and fuels ($NH_{\text{fuel},i}$). Nitrogen outputs in this subsystem ($NH_{\text{output},i}$) include human excretion ($NH_{\text{exc},i}$), waste generation ($NH_{\text{gar},i}$), and gaseous emissions ($NH_{\text{gas},i}$). The surplus nitrogen in households represents inefficiencies in nitrogen recycling and includes nitrogen that is not accounted for or retained within the household environment. The nitrogen cycle in the household subsystem can be expressed in supplementary equations (14)-(15).

Inputs:

$$NH_{\text{input},i} = NH_{\text{food},i} + NH_{\text{indu},i} + NH_{\text{fuel},i} \tag{14}$$

Outputs:

$$NH_{\text{output},i} = NH_{\text{exc},i} + NH_{\text{gar},i} + NH_{\text{gas},i} \tag{15}$$

Estimates for the nitrogen components in this subsystem are based on globally accepted data sources specific to various sectors. For food supply nitrogen, FAO's annual food protein supply data are utilized, and the nitrogen content is derived by applying standard protein-to-nitrogen conversion coefficients[27]. The nitrogen input from industrial products is based on output data from the industrial subsystem, with nitrogen estimates calculated from consumption patterns of materials used domestically. Fuel-related nitrogen inputs are determined by using data from the agricultural, forestry, and energy subsystems, which provide figures on fossil fuels, biomass, and other energy sources consumed within households. Nitrogen outputs such as human excretion are estimated through sanitation service data collected by the WHO, with distinctions made for urban and rural areas. Waste, including food and industrial product waste, is assessed based on information from the UNEP Food Waste Index Report (2021) and additional data on both structural and non-structural waste production[28]. Gas emissions are modeled using residential sector data from the CEDS database[11], which supplies emission factors for nitrogen oxides and other gaseous emissions resulting from household activities.

Industry nitrogen dynamics

In the industrial subsystem of country i, nitrogen inputs ($NI_{\text{input},i}$) are derived from several significant sources: Haber-Bosch nitrogen fixation ($NI_{\text{HBNF},i}$), materials from croplands ($NI_{\text{gra},i}$), livestock materials ($NI_{\text{ani},i}$), forest products ($NI_{\text{wood},i}$), and fuels ($NI_{\text{fuel},i}$). Outputs ($NI_{\text{output},i}$) from the industrial subsystem include nitrogen in fertilizers ($NI_{\text{fer},i}$), industrial products ($NI_{\text{ind},i}$), wastewater ($NI_{\text{was},i}$), runoff ($NI_{\text{runoff},i}$), waste ($NI_{\text{gar},i}$), and gaseous emissions ($NI_{\text{gas},i}$) that release nitrogen oxides such as NH_3, N_2O, and NO_x. Surplus nitrogen represents inefficiencies or nitrogen that is not utilized in industrial processes. The nitrogen cycle in the industrial subsystem can be expressed in supplementary equations (16)-(18).

Inputs:

$$NI_{\text{input},i} = NI_{\text{HBNF},i} + NI_{\text{gra},i} + NI_{\text{ani},i} + NI_{\text{wood},i} + NI_{\text{fuel},i} \tag{16}$$

Outputs:

$$NI_{\text{output},i} = NI_{\text{fer},i} + NI_{\text{ind},i} + NI_{\text{gar},i} + NI_{\text{was},i} + NI_{\text{gas},i} + NI_{\text{runoff},i} \tag{17}$$

Surplus:

$$NI_{\text{surplus},i} = NI_{\text{gas},i} + NI_{\text{runoff},i} \tag{18}$$

The nitrogen data for the industrial subsystem are derived from well-established global datasets and detailed methodological frameworks. Haber-Bosch nitrogen fixation is calculated by reverse engineering the nitrogen balance based on the total inputs and outputs, with corrections made using fertilizer production statistics for calibration. Contributions from cropland materials are based on FAO crop production data, with nitrogen content conversion coefficients specific to each crop like cotton or tobacco[6]. For livestock materials, nitrogen estimates are based on FAO statistics for livestock byproducts, with nitrogen conversion parameters applied to each type of byproduct[6]. Forest material contributions rely on FAO data on industrial roundwood production and related products[23]. Fuel inputs are modeled using energy system outputs, assuming a specific nitrogen content in the fuels used, based on available energy consumption data. The nitrogen in fertilizers is calculated using FAO's fertilizer production data[29], while industrial products are determined by assessing raw material efficiency, linked to income levels and industrial output scales. Nitrogen in wastewater is linked to data from the wastewater subsystem, with nitrogen losses from runoff estimated based on the difference between inputs and outputs in industrial processes[30, 31]. Waste and garbage nitrogen estimates come from industrial waste generation rates and demographic data[32]. Nitrogen gas emissions are estimated using comprehensive datasets such as CEDS and EDGAR[11, 33], which provide emission factors specific to different industrial and transport activities.

WTP nitrogen dynamics

In country i, the WTP subsystem manages nitrogen inputs primarily from domestic wastewater ($NW_{\text{dom},\,i}$) and industrial wastewater ($NW_{\text{ind},i}$). These nitrogen inputs undergo treatment processes aimed at reducing nitrogen loads before their final disposal or potential reuse. Nitrogen outputs ($NW_{\text{ind},i}$) from this subsystem include sludge nitrogen ($NW_{\text{sludge},i}$), gaseous emissions ($NW_{\text{gas},i}$), which comprise nitrogen compounds like NH_3, N_2O, and N_2 released during the treatment phases, and nitrogen losses through leaching and runoff ($NW_{\text{leaching\&runoff},\,i}$). Any discrepancies between nitrogen inputs and outputs are recorded as surplus nitrogen ($NW_{\text{surplus},i}$), reflecting inefficiencies, unmeasured losses, or nitrogen that accumulates within the system. The nitrogen cycle for the WTP subsystem can be expressed in supplementary equations (19)-(21).

Inputs:

$$NW_{\text{input},i} = NW_{\text{dom},\,i} + NW_{\text{ind},i} \tag{19}$$

Outputs:

$$NW_{\text{output},i} = NW_{\text{sludge},i} + NW_{\text{gas},i} + NW_{\text{leaching\&runoff},\,i} \tag{20}$$

Surplus:

$$NW_{\text{surplus},i} = NW_{\text{input},i} - NW_{\text{sludge},i} = NW_{\text{gas},i} + NW_{\text{leaching\&runoff},\,i} \tag{21}$$

The nitrogen inputs for the WTP subsystem are derived from reliable datasets and methodological frameworks. The volume of domestic wastewater is calculated using per capita wastewater generation rates, informed by studies such as those conducted by Jones et al[34]. These data are complemented by information on wastewater treatment coverage sourced from the UNEP's sustainable development goals (SDG) database[35]. For industrial wastewater inputs, national statistics on industrial water withdrawals are utilized[36], adjusted to account for recycling efficiencies and treatment coverage, which are categorized by income levels[37]. Outputs are clearly defined. The nitrogen content in sludge is estimated by considering the volume of treated wastewater and the sludge production efficiency, with an average nitrogen concentration of 18.84 g N/kg, as described in treatment studies[38]. Nitrogen-based gaseous emissions are calculated using emission data from the CEDS air pollution inventory and nitrogen balance methodologies tailored to N_2 emissions[39].

Leaching is approximated at 9% of nitrogen inputs using established empirical equations[40], while runoff nitrogen losses are estimated from the treated water's composition and national drainage standards[41]. This framework provides a comprehensive basis for understanding nitrogen flows within the WTP subsystem.

Garbage nitrogen dynamics

In country i, the garbage subsystem processes nitrogen inputs originating from several sources: municipal solid waste ($NG_{\text{msw},i}$), including household waste from urban areas, green waste, and commercial refuse; industrial waste ($NG_{\text{iw},i}$), which consists of solid nitrogen-containing byproducts from industrial activities; construction and demolition waste ($NG_{\text{cdw},i}$), which includes nitrogen-rich residues from building and infrastructure projects; and sludge ($NG_{\text{sludge},i}$), a solid byproduct transferred from the WTP subsystem. Nitrogen outputs in this subsystem ($NG_{\text{output},i}$) include recycled nitrogen ($NG_{\text{recyle},i}$), captured from waste recovery processes such as recycling and composting; composted nitrogen ($NG_{\text{compost},i}$), derived from organic waste treatment pathways; gaseous emissions ($NG_{\text{gas},i}$), which are released as NH_3, N_2O, and NO_x during treatment and degradation; and leached nitrogen ($NG_{\text{leaching},i}$), representing nitrogen losses into soil and groundwater. Any surplus nitrogen that is neither captured nor lost is retained within the subsystem, symbolizing inefficiencies or nitrogen that remains untracked. The nitrogen cycle in the garbage subsystem can be expressed in supplementary equations (22)-(23).

Inputs:

$$NG_{\text{input},i} = NG_{\text{msw},i} + NG_{\text{iw},i} + NG_{\text{cdw},i} + NG_{\text{sludge},i} \tag{22}$$

Outputs:

$$NG_{\text{output},i} = NG_{\text{recyle},i} + NG_{\text{compost},i} + NG_{\text{gas},i} + NG_{\text{leaching},i} \tag{23}$$

The nitrogen data for the garbage subsystem are gathered from several authoritative sources and robust methodologies. The estimation of municipal solid waste inputs follows IPCC 2019 classification standards and projections derived from the World Bank's "What a Waste 2.0" report[32, 42], which takes into account factors such as population growth, GDP trends, and regional waste generation patterns. Contributions from industrial waste are based on national and sector-specific data related to industrial activity. For construction and demolition waste, estimates rely on infrastructure growth metrics, with nitrogen content coefficients applied to building materials and byproducts. Sludge inputs are transferred directly from the WTP subsystem, incorporating data on nitrogen-rich solid residues. Outputs, such as recycled and composted nitrogen, are calculated based on regional recovery and treatment rates, as outlined in "What a Waste 2.0" and IPCC waste pathway statistics[32, 42]. Nitrogen gas emissions are modeled using emission factors for various waste treatment methods, including landfilling and incineration, adjusted for income-level distinctions. Leached nitrogen is determined using empirical soil infiltration models and nitrogen loss coefficients typical of landfill and unmanaged waste systems. This comprehensive framework ensures an accurate representation of nitrogen flows within the garbage subsystem.

Supplementary Method 4 | Uncertainty analysis.

The nitrogen intake data used in this study originate from the FAOSTAT Food Balance Sheets, a widely recognized and generally reliable source for global agricultural and food statistics. However, due to differences in reporting practices, data collection methods, and reliance on estimations between countries and regions, especially in countries with limited or inconsistent data, the quality and sources of the data still present variability and uncertainty. These uncertainties should be acknowledged when interpreting global nitrogen-related indicators. Our national-level nitrogen release inventory has been cross-validated using

multiple sources, including CEDS, EDGAR, IMAGE, and the CHANS model, although some uncertainties remain (Supplementary Fig. 6). One significant limitation is that nitrogen release from natural ecosystems, such as wetlands, oceans, and wildlife, is not included in our analysis. Accordingly, our estimates of nitrogen emissions to air and losses to water are limited to eight major sectors (Methods).

Aside from uncertainties related to data sources and models, this study uses per capita nitrogen intake and per capita nitrogen release to standardize the total nitrogen intakes and releases of each country, allowing for better comparability and analytical relevance. This approach has certain limitations, such as not using per hectare crop land nitrogen releases or comparing the absolute total nitrogen intakes and emissions. However, since the nitrogen releases in this study are based on a broader subsystem, including not only agricultural or forest/grassland releases but also nitrogen fluxes from industrial production and urban/rural residential sources, we chose the per capita standardization method to improve comparability between countries and regions.

To quantify the influence of nitrogen-related, social, and natural drivers on SDG progress, we employed Structural Equation Modeling (SEM) and Principal Component Analysis (PCA). The SEM results, based on data from 166 countries spanning 2000-2022, were derived from a series of tested specifications, with the final model demonstrating optimal fit (Supplementary Table 7). Key uncertainties in SEM, such as model specification and indicator definition, were carefully addressed through iterative model refinement. For PCA, five principal components were retained, preserving over 90% of the total variance and effectively reducing dimensionality without substantial information loss. Although PCA assumes linearity, this assumption was deemed acceptable when triangulated with multivariate regression results (Table 1).

In summary, while data and model uncertainties remain, they are within acceptable bounds. Continued efforts are needed to address these limitations and improve the precision and robustness of future analyses.

Supplementary Tables

Supplementary Table 1 | Region classifications and income definitions in this study.

Country name	ISO3	Region	Income group	Continent
Afghanistan	AFG	Other Asia	Low income	Asia
Albania	ALB	Europe	Upper middle income	Europe
Algeria	DZA	Middle East and North Africa	Lower middle income	Africa
Angola	AGO	Sub-Saharan Africa	Lower middle income	Africa
Antigua and Barbuda	ATG	Latin America	High income	Americas
Argentina	ARG	Latin America	Upper middle income	Americas
Armenia	ARM	Former Soviet Union	Upper middle income	Asia
Australia	AUS	Oceania	High income	Oceania
Austria	AUT	Europe	High income	Europe
Azerbaijan	AZE	Former Soviet Union	Upper middle income	Asia
Bahamas	BHS	Latin America	High income	Americas
Bahrain	BHR	Middle East and North Africa	High income	Asia
Bangladesh	BGD	Other Asia	Lower middle income	Asia
Barbados	BRB	Latin America	High income	Americas
Belarus	BLR	Former Soviet Union	Upper middle income	Europe
Belgium	BEL	Europe	High income	Europe
Belize	BLZ	Latin America	Lower middle income	Americas
Benin	BEN	Sub-Saharan Africa	Lower middle income	Africa
Bermuda	BMU	Latin America	High income	Americas
Bhutan	BTN	Other Asia	Lower middle income	Asia
Bolivia (Plurinational State of)	BOL	Latin America	Lower middle income	Americas
Bosnia and Herzegovina	BIH	Europe	Upper middle income	Europe
Botswana	BWA	Sub-Saharan Africa	Upper middle income	Africa
Brazil	BRA	Brazil	Upper middle income	Americas

Brunei Darussalam	BRN	Middle East and North Africa	High income	Asia
Bulgaria	BGR	Europe	Upper middle income	Europe
Burkina Faso	BFA	Sub-Saharan Africa	Low income	Africa
Burundi	BDI	Sub-Saharan Africa	Low income	Africa
Cambodia	KHM	Other Asia	Lower middle income	Asia
Cameroon	CMR	Sub-Saharan Africa	Lower middle income	Africa
Canada	CAN	North America	High income	Americas
Central African Republic	CAF	Sub-Saharan Africa	Low income	Africa
Chile	CHL	Latin America	High income	Americas
China	CHN	ChiNorth America	Upper middle income	Asia
Colombia	COL	Latin America	Upper middle income	Americas
Congo	COG	Sub-Saharan Africa	Lower middle income	Africa
Cook Islands	COK	Oceania	High income	Oceania
Costa Rica	CRI	Latin America	Upper middle income	Americas
Côte d'Ivoire	CIV	Sub-Saharan Africa	Lower middle income	Africa
Croatia	HRV	Europe	High income	Europe
Cuba	CUB	Latin America	Upper middle income	Americas
Cyprus	CYP	Europe	High income	Asia
Czechia	CZE	Europe	High income	Europe
Democratic Republic of the Congo	COD	Sub-Saharan Africa	Low income	Africa
Denmark	DNK	Europe	High income	Europe
Dominica	DMA	Latin America	Upper middle income	Americas
Dominican Republic	DOM	Latin America	Upper middle income	Americas
Ecuador	ECU	Latin America	Upper middle income	Americas
Egypt	EGY	Middle East and North Africa	Lower middle income	Africa
El Salvador	SLV	Latin America	Lower middle income	Americas
Eritrea	ERI	Sub-Saharan Africa	Low income	Africa
Estonia	EST	Europe	High income	Europe

Ethiopia	ETH	Sub-Saharan Africa	Low income	Africa
Fiji	FJI	Oceania	Upper middle income	Oceania
Finland	FIN	Europe	High income	Europe
France	FRA	Europe	High income	Europe
French Polynesia	PYF	Oceania	High income	Oceania
Gabon	GAB	Sub-Saharan Africa	Upper middle income	Africa
Gambia	GMB	Sub-Saharan Africa	Low income	Africa
Georgia	GEO	Former Soviet Union	Upper middle income	Asia
Germany	DEU	Europe	High income	Europe
Ghana	GHA	Sub-Saharan Africa	Lower middle income	Africa
Greece	GRC	Europe	High income	Europe
Guatemala	GTM	Latin America	Upper middle income	Americas
Guinea	GIN	Sub-Saharan Africa	Low income	Africa
Guyana	GUY	Latin America	Upper middle income	Americas
Honduras	HND	Latin America	Lower middle income	Americas
Hungary	HUN	Europe	High income	Europe
Iceland	ISL	Europe	High income	Europe
India	IND	India	Lower middle income	Asia
Indonesia	IDN	Other Asia	Lower middle income	Asia
Iran (Islamic Republic of)	IRN	Middle East and North Africa	Lower middle income	Asia
Iraq	IRQ	Middle East and North Africa	Upper middle income	Asia
Ireland	IRL	Europe	High income	Europe
Israel	ISR	Middle East and North Africa	High income	Asia
Italy	ITA	Europe	High income	Europe
Jamaica	JAM	Latin America	Upper middle income	Americas
Japan	JPN	Other Asia	High income	Asia
Jordan	JOR	Middle East and North Africa	Upper middle income	Asia
Kazakhstan	KAZ	Former Soviet Union	Upper middle income	Asia

Kenya	KEN	Sub-Saharan Africa	Lower middle income	Africa
Kuwait	KWT	Middle East and North Africa	High income	Asia
Kyrgyzstan	KGZ	Former Soviet Union	Lower middle income	Asia
Latvia	LVA	Europe	High income	Europe
Lebanon	LBN	Middle East and North Africa	Upper middle income	Asia
Libya	LBY	Middle East and North Africa	Upper middle income	Africa
Lithuania	LTU	Europe	High income	Europe
Luxembourg	LUX	Europe	High income	Europe
Madagascar	MDG	Sub-Saharan Africa	Low income	Africa
Malawi	MWI	Sub-Saharan Africa	Low income	Africa
Malaysia	MYS	Other Asia	Upper middle income	Asia
Maldives	MDV	Other Asia	Upper middle income	Asia
Mali	MLI	Sub-Saharan Africa	Low income	Africa
Malta	MLT	Middle East and North Africa	High income	Europe
Mauritius	MUS	Sub-Saharan Africa	Upper middle income	Africa
Mexico	MEX	Latin America	Upper middle income	Americas
Mongolia	MNG	Other Asia	Lower middle income	Asia
Montenegro	MNE	Europe	Upper middle income	Europe
Morocco	MAR	Middle East and North Africa	Lower middle income	Africa
Mozambique	MOZ	Sub-Saharan Africa	Low income	Africa
Myanmar	MMR	Other Asia	Lower middle income	Asia
Namibia	NAM	Sub-Saharan Africa	Upper middle income	Africa
Nauru	NRU	Oceania	High income	Oceania
Nepal	NPL	Other Asia	Lower middle income	Asia
Netherlands	NLD	Europe	High income	Europe
New Caledonia	NCL	Oceania	High income	Oceania
New Zealand	NZL	Oceania	High income	Oceania
Nicaragua	NIC	Latin America	Lower middle income	Americas

Niger	NER	Sub-Saharan Africa	Low income	Africa
Nigeria	NGA	Sub-Saharan Africa	Lower middle income	Africa
North Macedonia	MKD	Europe	Upper middle income	Europe
Norway	NOR	Europe	High income	Europe
Oman	OMN	Middle East and North Africa	High income	Asia
Pakistan	PAK	Other Asia	Lower middle income	Asia
Panama	PAN	Latin America	Upper middle income	Americas
Papua New Guinea	PNG	Oceania	Lower middle income	Oceania
Paraguay	PRY	Latin America	Upper middle income	Americas
Peru	PER	Latin America	Upper middle income	Americas
Philippines	PHL	Other Asia	Lower middle income	Asia
Poland	POL	Europe	High income	Europe
Portugal	PRT	Europe	High income	Europe
Qatar	QAT	Middle East and North Africa	High income	Asia
Republic of Korea	KOR	Other Asia	High income	Asia
Republic of Moldova	MDA	Former Soviet Union	Upper middle income	Europe
Romania	ROU	Europe	Upper middle income	Europe
Russian Federation	RUS	Former Soviet Union	Upper middle income	Europe
Rwanda	RWA	Sub-Saharan Africa	Low income	Africa
Saint Kitts and Nevis	KNA	Latin America	High income	Americas
Saint Lucia	LCA	Latin America	Upper middle income	Americas
Samoa	WSM	Oceania	Lower middle income	Oceania
Saudi Arabia	SAU	Middle East and North Africa	High income	Asia
Senegal	SEN	Sub-Saharan Africa	Lower middle income	Africa
Serbia	SRB	Europe	Upper middle income	Europe
Serbia and Montenegro	SCG			Europe
Seychelles	SYC	Sub-Saharan Africa	High income	Africa
Singapore	SGP	Other Asia	High income	Asia

Slovakia	SVK	Europe	High income	Europe
Slovenia	SVN	Europe	High income	Europe
South Africa	ZAF	Sub-Saharan Africa	Upper middle income	Africa
Spain	ESP	Europe	High income	Europe
Sri Lanka	LKA	Other Asia	Lower middle income	Asia
Sudan	SDN	Sub-Saharan Africa	Low income	Africa
Suriname	SUR	Latin America	Upper middle income	Americas
Sweden	SWE	Europe	High income	Europe
Switzerland	CHE	Europe	High income	Europe
Syrian Arab Republic	SYR	Middle East and North Africa	Low income	Asia
Tajikistan	TJK	Former Soviet Union	Lower middle income	Asia
Thailand	THA	Other Asia	Upper middle income	Asia
Togo	TGO	Sub-Saharan Africa	Low income	Africa
Tonga	TON	Oceania	Upper middle income	Oceania
Trinidad and Tobago	TTO	Latin America	High income	Americas
Tunisia	TUN	Middle East and North Africa	Lower middle income	Africa
Turkey	TUR	Middle East and North Africa	Upper middle income	Asia
Uganda	UGA	Sub-Saharan Africa	Low income	Africa
Ukraine	UKR	Former Soviet Union	Lower middle income	Europe
United Arab Emirates	ARE	Middle East and North Africa	High income	Asia
United Kingdom of Great Britain and Northern Ireland	GBR	Europe	High income	Europe
United Republic of Tanzania	TZA	Sub-Saharan Africa	Lower middle income	Africa
United States of America	USA	North America	High income	Americas
Uruguay	URY	Latin America	High income	Americas
Uzbekistan	UZB	Former Soviet Union	Lower middle income	Asia
Venezuela (Bolivarian Republic of)	VEN	Latin America	Lower middle income	Americas
Viet Nam	VNM	Other Asia	Lower middle income	Asia
Yemen	YEM	Middle East and North Africa	Low income	Asia

Zambia	ZMB	Sub-Saharan Africa	Lower middle income	Africa
Zimbabwe	ZWE	Sub-Saharan Africa	Lower middle income	Africa

Supplementary Table 2 | Impact of nitrogen-related factors on Sustainable Development Goal (SDG) index in different regions.

	Brazil	China	Europe	FSU	India	LA	MENA	NA	OA	Oceania	SSA
Plant-based nitrogen intake	-1.547	-0.148	0.084	-1.298**	5.583***	1.104**	1.096**	-2.463***	9.656***	0.826	5.247***
(kg nitrogen capita^{-1})	(1.113)	(2.334)	(0.257)	(0.553)	(1.912)	(0.428)	(0.505)	(0.632)	(0.447)	(0.811)	(0.333)
Animal-based nitrogen intake	1.750***	8.378***	3.880***	5.430***	17.312***	4.536***	5.778***	2.172***	1.600***	1.389***	5.010***
(kg nitrogen capita^{-1})	(0.479)	(2.443)	(0.149)	(0.334)	(2.224)	(0.230)	(0.339)	(0.359)	(0.338)	(0.362)	(0.430)
Nitrogen release to air	0.102	0.126	-0.282***	0.125	0.514	-0.051*	-0.402***	-0.117***	0.013	-0.027**	-0.043***
(kg nitrogen capita^{-1})	(0.158)	(0.580)	(0.023)	(0.088)	(1.290)	(0.030)	(0.058)	(0.019)	(0.067)	(0.013)	(0.014)
Nitrogen release to water	1.405**	-3.574*	0.219***	0.167	-0.575	-0.036	0.421***	-0.066	-0.159	-0.118	-0.429***
(kg nitrogen capita^{-1})	(0.493)	(1.736)	(0.041)	(0.181)	(2.153)	(0.071)	(0.068)	(0.112)	(0.100)	(0.109)	(0.152)
Region	Yes	Yes	Yes	Yes	Yes	Yes	Yes	Yes	Yes	Yes	Yes
Year	Yes	Yes	Yes	Yes	Yes	Yes	Yes	Yes	Yes	Yes	Yes
N	23	23	793	253	23	667	402	46	372	113	740
Adjusted R^2	0.946	0.970	0.582	0.618	0.969	0.498	0.457	0.925	0.666	0.340	0.355

Standard errors in parentheses

* $p < 0.1$, ** $p < 0.05$, *** $p < 0.01$

Note: Brazil, China and India are reported separately. FSU denotes the Former Soviet Union; LA denotes Latin America excluding Brazil; MENA denotes the Middle East and North Africa; NA denotes North America; OA denotes Other Asia excluding China and India; and SSA denotes Sub-Saharan Africa. Europe excludes countries included in the FSU, while Oceania includes Australia, New Zealand and Pacific island countries.

Supplementary Table 3 | Impact of nitrogen-related factors on Economy performance in different regions.

	Brazil	China	Europe	FSU	India	LA	MENA	NA	OA	Oceania	SSA
Plant-based nitrogen intake	-0.334	0.491	0.416*	-0.74	2.874*	2.155***	0.984*	-4.317***	7.549***	1.053	3.410***
(kg nitrogen capita^{-1})	(0.912)	(2.137)	(0.252)	(0.458)	(1.483)	(0.370)	(0.547)	(0.946)	(0.449)	(0.694)	(0.249)
Animal-based nitrogen intake	1.930***	7.311***	3.606***	4.311***	13.809***	3.307***	4.895***	2.646***	1.919***	1.085***	4.178***
(kg nitrogen capita^{-1})	(0.392)	(2.237)	(0.146)	(0.276)	(1.725)	(0.198)	(0.368)	(0.536)	(0.339)	(0.310)	(0.321)
Nitrogen release to air	0.09	-0.049	-0.298***	0.029	-0.739	-0.070***	-0.416***	-0.122***	-0.039	0.004	-0.049***
(kg nitrogen capita^{-1})	(0.130)	(0.531)	(0.023)	(0.073)	(1.000)	(0.026)	(0.062)	(0.028)	(0.067)	(0.011)	(0.010)
Nitrogen release to water	0.782*	-3.459**	0.270***	-0.216	0.11	-0.05	0.426***	-0.111	-0.153	-0.255***	-0.580***
(kg nitrogen capita^{-1})	(0.404)	(1.589)	(0.040)	(0.150)	(1.670)	(0.062)	(0.074)	(0.167)	(0.100)	(0.093)	(0.113)
Region	Yes	Yes	Yes	Yes	Yes	Yes	Yes	Yes	Yes	Yes	Yes
Year	Yes	Yes	Yes	Yes	Yes	Yes	Yes	Yes	Yes	Yes	Yes
N	23	23	793	253	23	667	402	46	372	113	740
Adjusted R^2	0.948	0.972	0.571	0.556	0.960	0.474	0.352	0.880	0.578	0.333	0.359

Standard errors in parentheses
$* p < 0.1, ** p < 0.05, *** p < 0.01$

Note: Brazil, China and India are reported separately. FSU denotes the Former Soviet Union; LA denotes Latin America excluding Brazil; MENA denotes the Middle East and North Africa; NA denotes North America; OA denotes Other Asia excluding China and India; and SSA denotes Sub-Saharan Africa. Europe excludes countries included in the FSU, while Oceania includes Australia, New Zealand and Pacific island countries.

Supplementary Table 4 | Impact of nitrogen-related factors on Society performance in different regions.

	Brazil	China	Europe	FSU	India	LA	MENA	NA	OA	Oceania	SSA
Plant-based nitrogen intake	-2.49	-0.888	0.01	-2.633***	11.991**	0.379	1.687**	0.161	16.753***	2.146	9.157***
(kg nitrogen capita^{-1})	(1.941)	(4.788)	(0.270)	(0.967)	(4.173)	(0.757)	(0.756)	(0.567)	(0.747)	(1.389)	(0.603)
Animal-based nitrogen intake	2.213**	15.383***	3.864***	8.759***	29.012***	7.228***	9.385***	0.396	2.415***	2.352***	8.164***
(kg nitrogen capita^{-1})	(0.834)	(5.013)	(0.157)	(0.584)	(4.853)	(0.406)	(0.508)	(0.321)	(0.564)	(0.621)	(0.778)
Nitrogen release to air	0.173	0.346	-0.174***	0.264*	4.384	0.05	-0.107	-0.090***	0.116	-0.03	-0.045*
(kg nitrogen capita^{-1})	(0.276)	(1.190)	(0.025)	(0.155)	(2.814)	(0.053)	(0.086)	(0.017)	(0.112)	(0.023)	(0.025)
Nitrogen release to water	2.417**	-5.39	0.086**	0.844***	-4.043	0.04	0.079	0.022	-0.207	-0.056	-0.401
(kg nitrogen capita^{-1})	(0.860)	(3.561)	(0.043)	(0.318)	(4.697)	(0.126)	(0.102)	(0.100)	(0.167)	(0.186)	(0.275)
Region	Yes	Yes	Yes	Yes	Yes	Yes	Yes	Yes	Yes	Yes	Yes
Year	Yes	Yes	Yes	Yes	Yes	Yes	Yes	Yes	Yes	Yes	Yes
N	23	23	793	253	23	667	402	46	372	113	740
Adjusted R^2	0.928	0.958	0.517	0.614	0.961	0.432	0.480	0.794	0.681	0.313	0.322

Standard errors in parentheses

* $p < 0.1$, ** $p < 0.05$, *** $p < 0.01$

Note: Brazil, China and India are reported separately. FSU denotes the Former Soviet Union; LA denotes Latin America excluding Brazil; MENA denotes the Middle East and North Africa; NA denotes North America; OA denotes Other Asia excluding China and India; and SSA denotes Sub-Saharan Africa. Europe excludes countries included in the FSU, while Oceania includes Australia, New Zealand and Pacific island countries.

Supplementary Table 5 | Impact of nitrogen-related factors on Environment performance in different regions.

	Brazil	China	Europe	FSU	India	LA	MENA	NA	OA	Oceania	SSA
Plant-based nitrogen intake	-3.356***	0.347	-0.799	0.134	-0.077	-0.532	0.479	-3.353**	0.435	-1.834**	1.878***
(kg nitrogen capita^{-1})	(0.720)	(1.152)	(0.538)	(0.323)	(1.509)	(0.559)	(0.645)	(1.564)	(0.424)	(0.919)	(0.302)
Animal-based nitrogen intake	0.415	-3.397**	4.540***	0.570***	2.659	2.218***	1.248***	4.521***	-0.885***	0.153	0.492
(kg nitrogen capita^{-1})	(0.309)	(1.206)	(0.311)	(0.195)	(1.755)	(0.300)	(0.433)	(0.887)	(0.320)	(0.410)	(0.390)
Nitrogen release to air	0.045	0.139	-0.477***	0.158***	-3.913***	-0.149***	-0.814***	-0.149***	-0.015	-0.102***	-0.014
(kg nitrogen capita^{-1})	(0.102)	(0.286)	(0.049)	(0.052)	(1.018)	(0.039)	(0.074)	(0.047)	(0.064)	(0.015)	(0.013)
Nitrogen release to water	0.856**	0.094	0.354***	-0.473***	4.936***	-0.098	0.929***	-0.139	-0.299***	0.179	-0.048
(kg nitrogen capita^{-1})	(0.319)	(0.857)	(0.085)	(0.106)	(1.699)	(0.093)	(0.087)	(0.276)	(0.095)	(0.123)	(0.138)
Region	Yes	Yes	Yes	Yes	Yes	Yes	Yes	Yes	Yes	Yes	Yes
Year	Yes	Yes	Yes	Yes	Yes	Yes	Yes	Yes	Yes	Yes	Yes
N	23	23	793	253	23	667	402	46	372	113	740
Adjusted R^2	0.902	0.937	0.357	0.096	0.705	0.107	0.251	0.845	0.114	0.323	0.056

Standard errors in parentheses

* $p < 0.1$, ** $p < 0.05$, *** $p < 0.01$

Note: Brazil, China and India are reported separately. FSU denotes the Former Soviet Union; LA denotes Latin America excluding Brazil; MENA denotes the Middle East and North Africa; NA denotes North America; OA denotes Other Asia excluding China and India; and SSA denotes Sub-Saharan Africa. Europe excludes countries included in the FSU, while Oceania includes Australia, New Zealand and Pacific island countries.

Supplementary Table 6 | Standardized path coefficients and *P*-values in structural equation model.

Dependent variable	Independent variable	Coefficients	*P*-values
SDG index	Nitrogen intake (kg N $capita^{-1}$)	0.460	0.000
	Nitrogen release (kg N $capita^{-1}$)	-0.096	0.000
	PGDP ($)	0.115	0.000
	Urbanization (%)	0.227	0.000
	Mean annual temperature (°C)	-0.341	0.000
	Mean annual precipitation (mm)	0.220	0.000
Nitrogen intake (kg N $capita^{-1}$)	PGDP ($)	0.502	0.000
	Urbanization (%)	0.208	0.000
	Mean annual temperature (°C)	-0.273	0.000
	Mean annual precipitation (mm)	-0.073	0.000
Nitrogen release (kg N $capita^{-1}$)	PGDP ($)	0.257	0.000
	Urbanization (%)	0.151	0.000
	Mean annual temperature (°C)	-0.097	0.000
	Mean annual precipitation (mm)	0.021	0.169

Supplementary Table 7 | Structural equation model fitting index.

Model	*P*-values	CFI	RMSEA	SRMR
Fig. 2a	0.638	1.000	0.000	0.001

Supplementary Figures

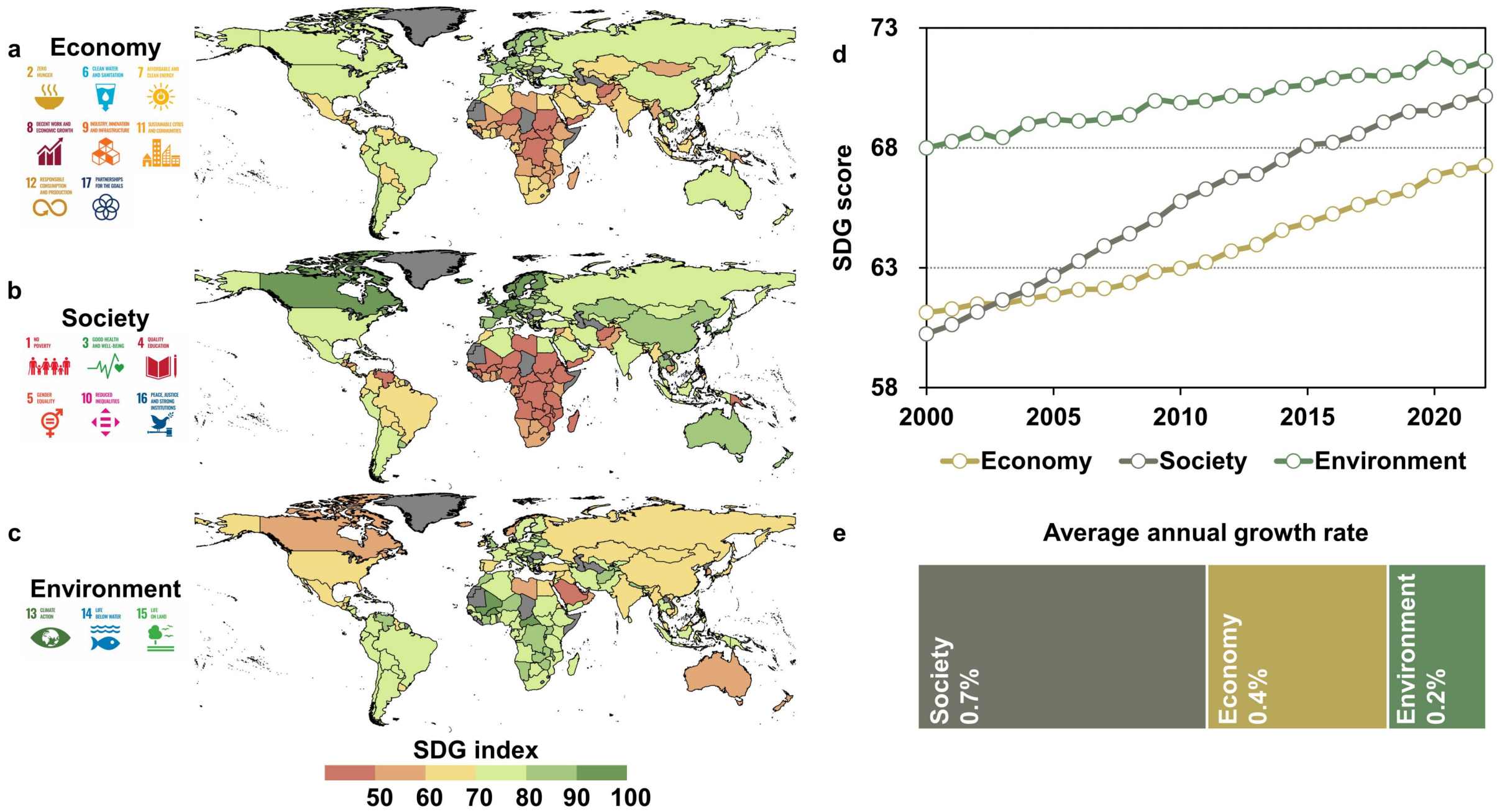

Supplementary Fig. 1 | Trends of Sustainable Development Goal (SDG) index and performance in "Economy," "Society," and "Environment" from 2000 to 2022. (a-c) Scores of the three SDG categories: "Economy" (a), "Society" (b), and "Environment" (c) for each country in 2022. (d) Trends of score changes in the three SDG categories. (e) Annual average growth rate of the three SDG categories from 2000 to 2022. Grey area in maps (a), (b), and (c) indicate missing data. The global SDG index data is sourced from the United Nations 2025 Sustainable Development Report (https://dashboards.sdgindex.org/). The base map with administrative boundaries was created using data from the GADM (https://gadm.org/data.html), without endorsement. The SDG icons were obtained from the United Nations Sustainable Development Goals website (https://www.un.org/sustainabledevelopment). The content of this publication has not been approved by the United Nations and does not reflect the views of the United Nations or its officials or Member States.

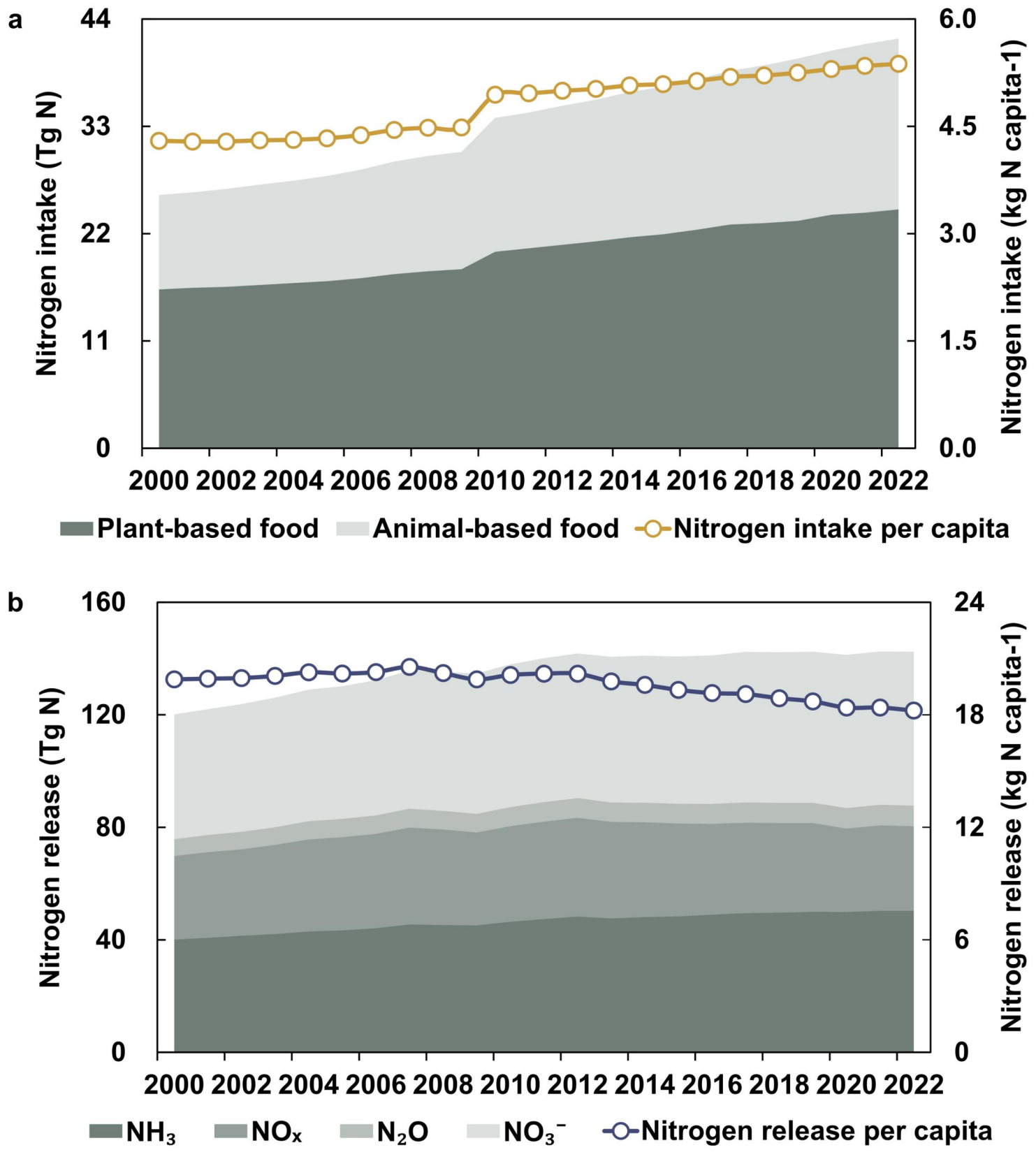

Supplementary Fig. 2 | Historical trends of nitrogen intake and release. (a) Total nitrogen intake from plant-based and animal-based food, as well as per capita nitrogen intake from 2000 to 2022. Nitrogen intake data was calculated based on the FAO Food Balances database (https://doi.org/10.4060/cd7162en), covering plant-based and animal-based food categories. An apparent step change around 2010 in dietary nitrogen intake may reflect updates to the FAO Food Balances series (e.g., a transition between database versions and/or revisions in balancing procedures, commodity coverage, and conversion factors), changes in country reporting and data completeness, and associated recalibration or imputation in the underlying food supply statistics. We therefore interpret this discontinuity as a potential data artifact rather than an abrupt real-world shift. (b) Total nitrogen release to air and to water, as well as per capita nitrogen release from 2000 to 2022. Nitrogen release data is based on the CHANS model outputs.

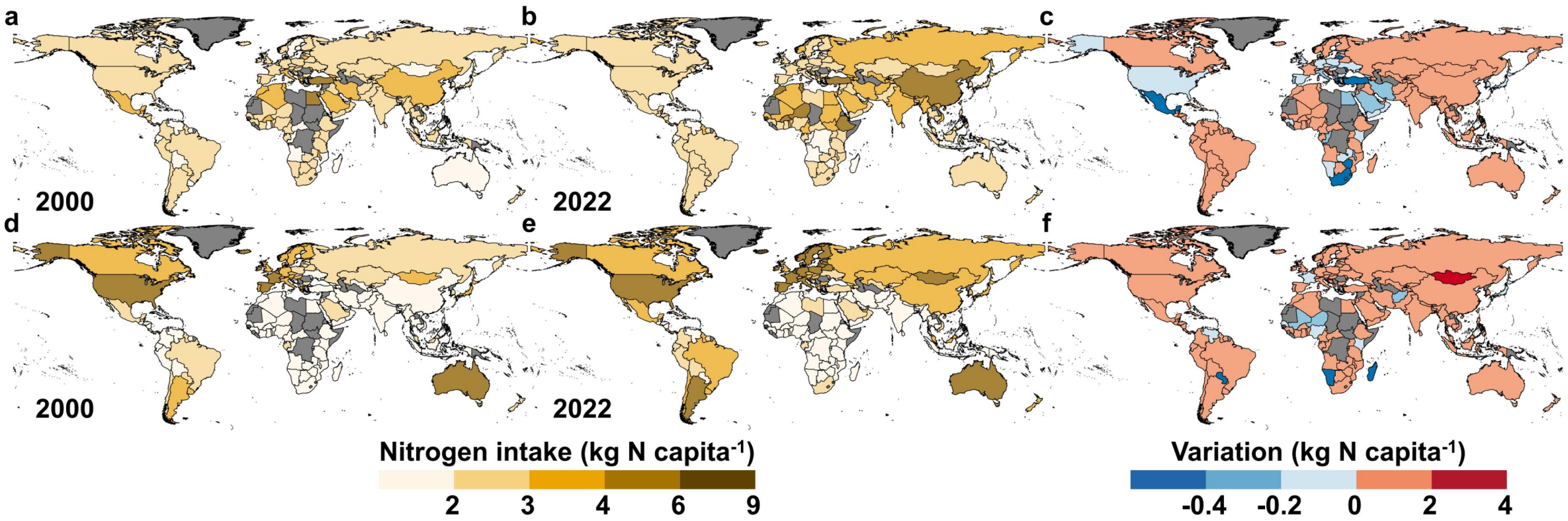

Supplementary Fig. 3 | Global patterns and changes in dietary nitrogen intake from plant- and animal-based foods from 2000 to 2022. (a, b) Spatial distribution of nitrogen intake from plant-based food in 2000 and 2022, respectively. (c) Change in nitrogen intake from plant-based food from 2000 to 2022. (d, e) Spatial distribution of nitrogen intake from animal-based food in 2000 and 2022, respectively. (f) Change in nitrogen intake from animal-based food from 2000 to 2022. Dietary nitrogen intake was calculated from the FAO Food Balances database (https://doi.org/10.4060/cd7162en). All values are expressed as kg N capita^{-1}; changes represent values in 2022 minus values in 2000. Grey areas indicate missing data. The base map with administrative boundaries was created using data from GADM (https://gadm.org/data.html), without endorsement.

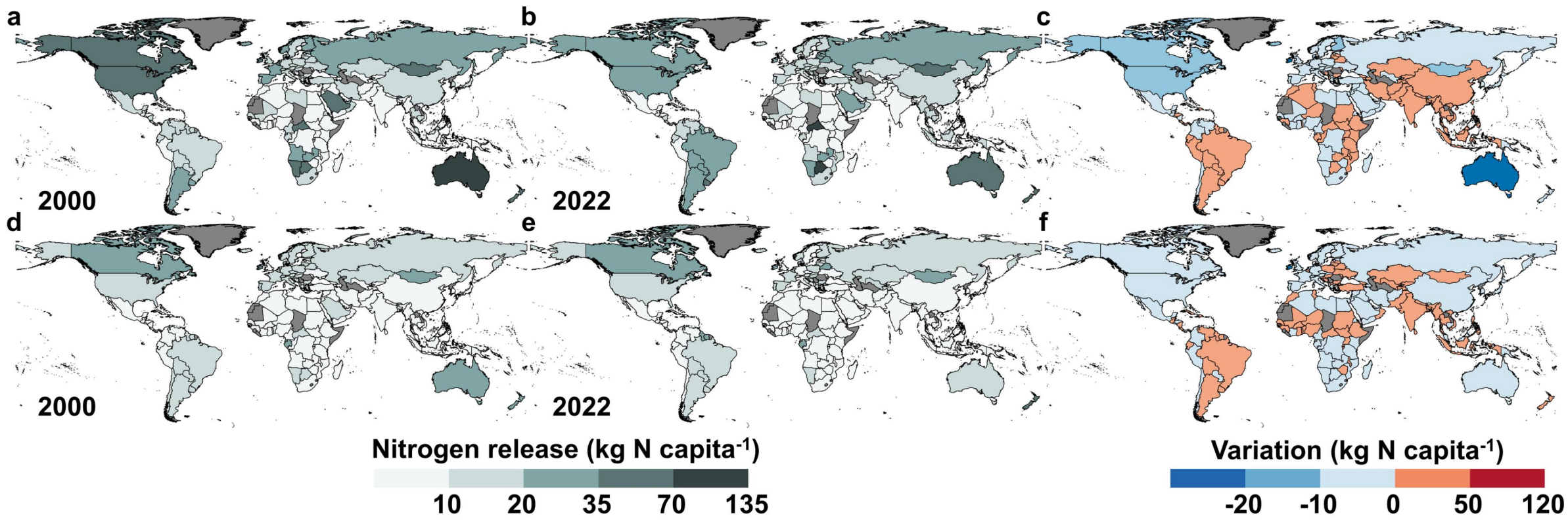

Supplementary Fig. 4 | Global patterns and changes in nitrogen release to air and water from 2000 to 2022. (a, b) Spatial distribution of nitrogen release to air in 2000 and 2022, respectively. (c) Change in nitrogen release to air from 2000 to 2022. (d, e) Spatial distribution of nitrogen release to water in 2000 and 2022, respectively. (f) Change in nitrogen release to water from 2000 to 2022. Nitrogen release to air includes NH_3, NO_x, and N_2O emissions, and nitrogen release to water includes NO_3^- losses through leaching and runoff, estimated using the CHANS model. All values are expressed as kg N capita^{-1}; changes represent values in 2022 minus values in 2000. Grey areas indicate missing data. The base map with administrative boundaries was created using data from GADM (https://gadm.org/data.html), without endorsement.

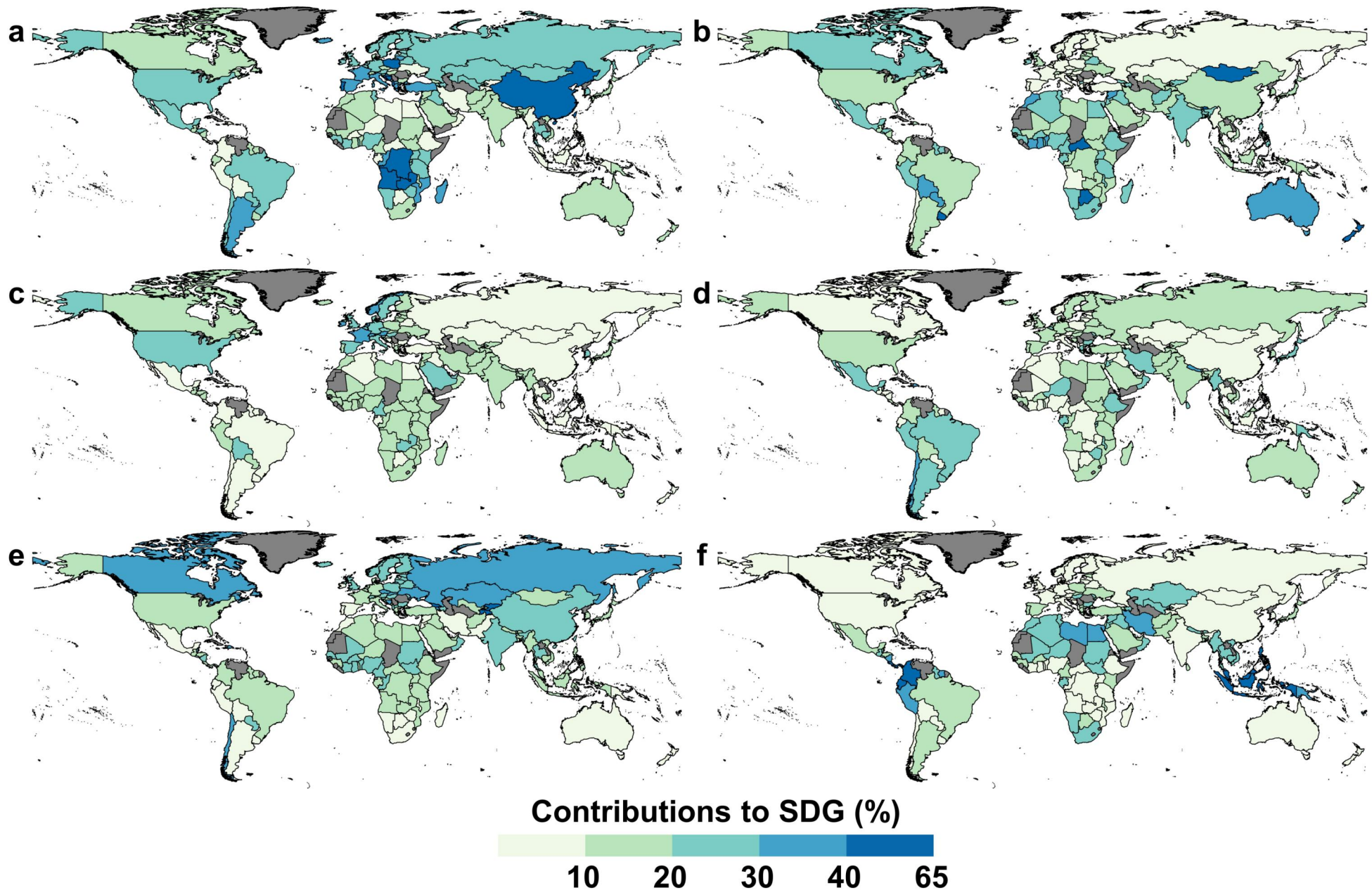

Supplementary Fig. 5 | Spatial distribution of nitrogen-related, social, and natural factors' contributions to the Sustainable Development Goal (SDG) index in 2022. Spatial distribution of the contributions of nitrogen intake (a), nitrogen release (b), per capita GDP (c), urbanization rate (d), mean annual temperature (e), and mean annual precipitation (f) to the SDG index in 2022. Grey area in maps indicate missing data. The base map with administrative boundaries was created using data from the GADM (https://gadm.org/data.html), without endorsement.

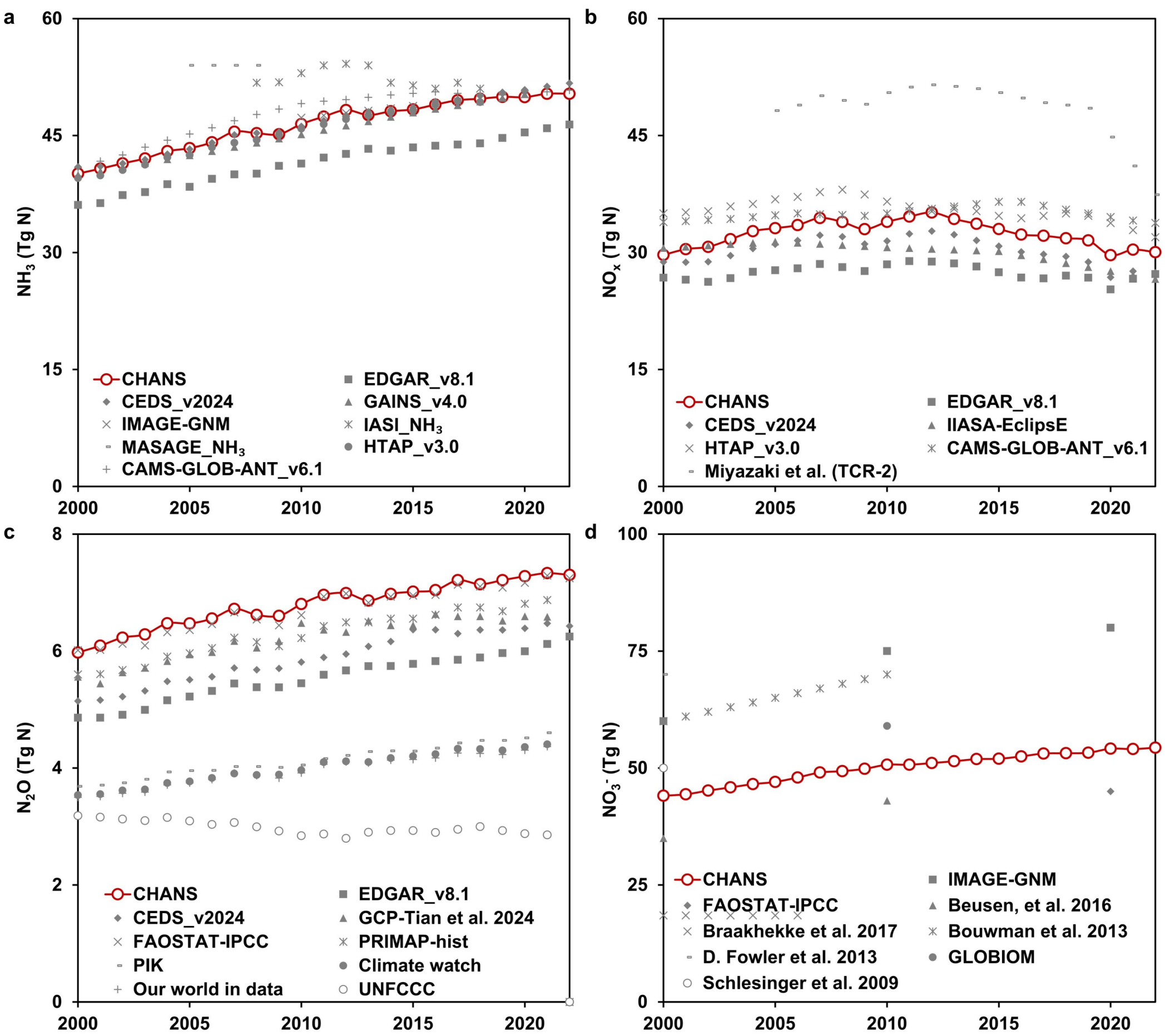

Supplementary Fig. 6 | Comparison of nitrogen release to air and water from the Coupled Human And Natural Systems (CHANS) model with other models and literature. This figure presents a comparison of global nitrogen emissions to air (NH_3 (a), NO_x (b), N_2O (c)) and nitrogen losses to water (NO_3^- (d)) as estimated by the CHANS model, alongside estimates from other models and relevant literature[43-64]. "CHANS" refers to the Coupled Human and Natural Systems model used in this study; "EDGAR_v8.1" refers to The Emissions Database for Global Atmospheric Research version 8.1; "CEDS_v2024" represents the Community Earth-atmosphere Data System version 2024; "GAINS_v4.0" refers to the Greenhouse Gas and Air Pollution Interactions and Synergies model version 4.0; "IMAGE-GNM" stands for the Integrated Model to Assess the Global Environment - Global Nutrient Model; "IASI_NH3" refers to the Infrared Atmospheric Sounding Interferometer retrieval of NH_3; "MASAGE_NH3" represents the Magnitude And Seasonality of Agricultural Emissions model for NH_3; "HTAP_v3.0" refers to the Hemispheric Transport of Air Pollution experiment version 3.0; "CAMS-GLOB-ANT_v6.1" represents the Global anthropogenic emissions for the Copernicus Atmosphere Monitoring Service simulations version 6.1; "IIASA-EclipsE" refers to the Evaluating the Climate and Air Quality Impacts of Short-Lived Pollutants Project by the International Institute for Applied Systems Analysis; "FAOSTAT-IPCC" refers to the FAOSTAT Emissions Totals Database developed by the Food and Agriculture Organization (FAO), aligned with the Intergovernmental Panel on Climate Change (IPCC); "PRIMAP-hist" stands for the Potsdam Realtime Integrated Model for probabilistic Assessment of emissions Paths dataset; "PIK" refers to the Potsdam Institute for Climate Impact Research; "UNFCCC" represents the United Nations Framework Convention on Climate Change; "GLOBIOM" refers to the Global Biosphere Management Model.

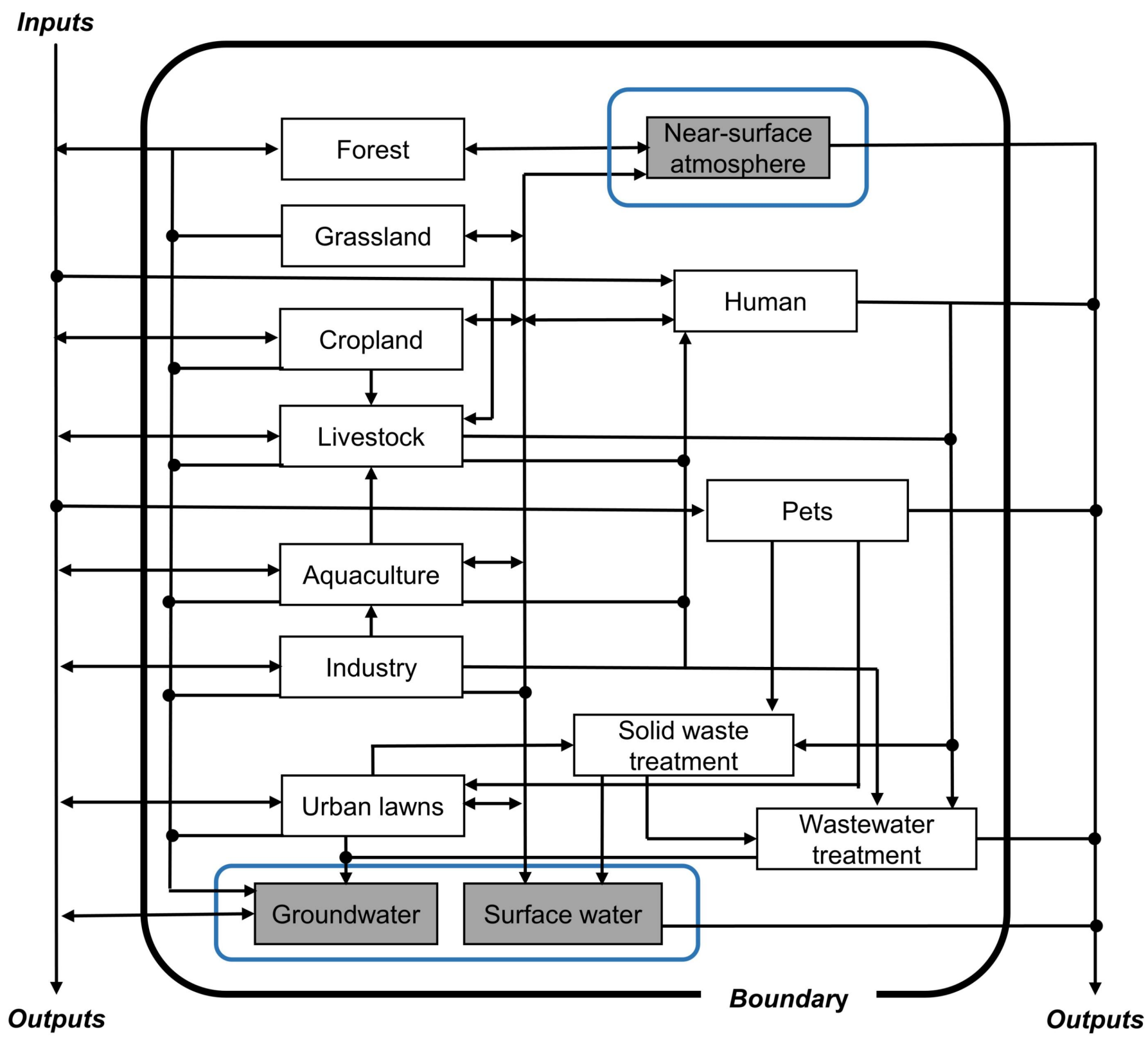

Supplementary Fig. 7 | Nitrogen dynamics in the Coupled Human and Natural Systems (CHANS) model. Arrows indicate nitrogen fluxes; solid rectangular boxes represent the subsystems and the system; dashed lines delineate the boundaries of functional groups. Modified from Gu et al[65].

Supplementary References:

1. Lassaletta, L., *et al*. Nitrogen use in the global food system: past trends and future trajectories of agronomic performance, pollution, trade, and dietary demand. *Environ Res Lett* **11**, 95007 (2016). 10.1088/1748-9326/11/9/095007
2. IFA. International Fertilizer Association (2024). https://www.fertilizer.org/
3. Gu, B., *et al*. Cost-effective mitigation of nitrogen pollution from global croplands. *Nature* **613**, 77-84 (2023). 10.1038/s41586-022-05481-8
4. PBL. Integrated Model to Assess the Global Environment (IMAGE) model Documentation (2020). https://models.pbl.nl/image/index.php/Welcome_to_IMAGE_3.2_Documentation
5. Dietrich, J.P., *et al*. MAgPIE - An Open Source land-use modeling framework - Version 4.0 (2018). https://rse.pik-potsdam.de/doc/magpie/4.0/
6. FAO. Production / Crops and livestock products - Metadata (2025). https://www.fao.org/faostat/en/#data/QCL/metadata
7. Cheng, L., *et al*. A 12% switch from monogastric to ruminant livestock production can reduce emissions and boost crop production for 525 million people. *Nat Food* **3**, 1040-1051 (2022). 10.1038/s43016-022-00661-1
8. Cheng, L., Zhang, X., Wang, C., Deng, O. & Gu, B. Whole-chain intensification of pig and chicken farming could lower emissions with economic and food production benefits. *Nat Food* **5**, 939-950 (2024). 10.1038/s43016-024-01067-x
9. FAO. Climate Change: Agrifood systems emissions / Emissions from Livestock - Metadata (2025). https://www.fao.org/faostat/en/#data/GLE/metadata
10. Wang, H., *et al*. Strategies to reduce ammonia emissions from livestock and their cost-benefit analysis: A case study of Sheyang county. *Environ Pollut* **290**, 118045 (2021). 10.1016/j.envpol.2021.118045
11. Hoesly, R.M., *et al*. Historical (1750–2014) anthropogenic emissions of reactive gases and aerosols from the Community Emissions Data System (CEDS). *Geosci Model Dev* **11**, 369-408 (2018). 10.5194/gmd-11-369-2018
12. Lassaletta, L., Billen, G., Grizzetti, B., Anglade, J. & Garnier, J. 50 year trends in nitrogen use efficiency of world cropping systems: the relationship between yield and nitrogen input to cropland. *Environ Res Lett* **9**, 105011-105019 (2014). 10.1088/1748-9326/9/10/105011
13. Xu, R., *et al*. Increased nitrogen enrichment and shifted patterns in the world's grassland: 1860–2016. *Earth Syst Sci Data* **11**, 175-187 (2019). 10.5194/essd-11-175-2019
14. Davies Barnard, T. & Friedlingstein, P. The Global Distribution of Biological Nitrogen Fixation in Terrestrial Natural Ecosystems. *Global Biogeochem Cycles* **34** (2020). 10.1029/2019GB006387
15. Bouwman, A.F., Boumans, L.J.M. & Batjes, N.H. Estimation of global NH_3 volatilization loss from synthetic fertilizers and animal manure applied to arable lands and grasslands. *Global Biogeochem Cycles* **16** (2002). 10.1029/2000GB001389
16. Pan, B., Lam, S.K., Mosier, A., Luo, Y. & Chen, D. Ammonia volatilization from synthetic fertilizers and its mitigation strategies: A global synthesis. *Agriculture, Ecosystems & Environment* **232**, 283-289 (2016). 10.1016/j.agee.2016.08.019
17. Dangal, S.R.S., *et al*. Global Nitrous Oxide Emissions From Pasturelands and Rangelands: Magnitude, Spatiotemporal Patterns, and Attribution. *Global Biogeochem Cycles* **33**, 200-222 (2019). 10.1029/2018GB006091
18. Wei, Z., Hoffland, E., Zhuang, M., Hellegers, P. & Cui, Z. Organic inputs to reduce nitrogen export via leaching and runoff: A global meta-analysis. *Environ Pollut* **291**, 118176 (2021).

10.1016/j.envpol.2021.118176
19. Smethurst, P.J. Forest fertilization: Trends in knowledge and practice compared to agriculture. *Plant Soil* **335**, 83-100 (2010). 10.1007/s11104-010-0316-3
20. Pukkala, T. Optimal nitrogen fertilization of boreal conifer forest. *For Ecosyst* **4** (2017). 10.1186/s40663-017-0090-2
21. Sullivan, B.W.*, et al*. Spatially robust estimates of biological nitrogen (N) fixation imply substantial human alteration of the tropical N cycle. *Proceedings of the National Academy of Sciences* **111**, 8101-8106 (2014). 10.1073/pnas.1320646111
22. Liu, L.*, et al*. Modeling global oceanic nitrogen deposition from food systems and its mitigation potential by reducing overuse of fertilizers. *Proceedings of the National Academy of Sciences* **120** (2023). 10.1073/pnas.2221459120
23. FAO. Forestry Production and Trade (2025). https://www.fao.org/faostat/en/#data/FO
24. GFW. Global forest watch-umd_tree_cover_loss_from_fires (2024). https://www.globalforestwatch.org/dashboards/global/?category=fires
25. Thompson, R.L.*, et al*. Acceleration of global N2O emissions seen from two decades of atmospheric inversion. *Nat Clim Chang* **9**, 993-998 (2019). 10.1038/s41558-019-0613-7
26. Bray, C.D., Battye, W.H., Aneja, V.P. & Schlesinger, W.H. Global emissions of NH(3), NO(x), and N(2)O from biomass burning and the impact of climate change. *J Air Waste Manag Assoc* **71**, 102-114 (2021). 10.1080/10962247.2020.1842822
27. FAOSTAT. Food Balances (2010-) (2025). https://www.fao.org/faostat/
28. UNEP. Food Waste Index Report. (2021).
29. FAO. Fertilizers by Product (2025). https://www.fao.org/faostat/en/#data/RFB
30. Statista. https://www.statista.com/ (2025).
31. Gu, B.*, et al*. The role of industrial nitrogen in the global nitrogen biogeochemical cycle. *Sci Rep* **3** (2013). 10.1038/srep02579
32. Kaza, S., Yao, L.C., Bhada-Tata, P. & Van Woerden, F. *What a waste 2.0: a global snapshot of solid waste management to 2050* (World Bank, 2018).
33. Crippa, M.*, et al*. Insights into the spatial distribution of global, national, and subnational greenhouse gas emissions in the Emissions Database for Global Atmospheric Research (EDGAR v8.0). *Earth Syst Sci Data* **16**, 2811-2830 (2024). 10.5194/essd-16-2811-2024
34. Jones, E.R., van Vliet, M.T.H., Qadir, M. & Bierkens, M.F.P. Country-level and gridded estimates of wastewater production, collection, treatment and reuse. *Earth Syst Sci Data* **13**, 237-254 (2021). 10.5194/essd-13-237-2021
35. UNEP. World Health Organization and United Nations Human Settlements Programme (UN-Habitat) (2025). https://sdg6data.org/en
36. FAO. AQUASTAT - FAO's Global Information System on Water and Agriculture (2025). https://www.fao.org/aquastat/en/databases/
37. Flörke, M.*, et al*. Domestic and industrial water uses of the past 60 years as a mirror of socio-economic development: A global simulation study. *Global Environmental Change* **23**, 144-156 (2013). 10.1016/j.gloenvcha.2012.10.018
38. Mateo-Sagasta, J., Raschid-Sally, L. & Thebo, A. Global Wastewater and Sludge Production, Treatment and Use. *Wastewater: Economic asset in an urbanizing world*, 15-38 (2015). 10.1007/978-94-017-9545-6_2
39. McDuffie, E.E.*, et al*. A global anthropogenic emission inventory of atmospheric pollutants from

sector- and fuel-specific sources (1970–2017): an application of the Community Emissions Data System (CEDS). *Earth Syst Sci Data* **12**, 3413-3442 (2020). 10.5194/essd-12-3413-2020
40. Gu, B., Ju, X., Chang, J., Ge, Y. & Vitousek, P.M. Integrated reactive nitrogen budgets and future trends in China. *Proceedings of the National Academy of Sciences - PNAS* **112**, 8792-8797 (2015). 10.1073/pnas.1510211112
41. Van Drecht, G., Bouwman, A.F., Harrison, J. & Knoop, J.M. Global nitrogen and phosphate in urban wastewater for the period 1970 to 2050. *Global Biogeochem Cycles* **23** (2009). 10.1029/2009GB003458
42. IPCC. 2019 Refinement to the 2006 IPCC Guidelines for National Greenhouse Gas Inventories. (2019).
43. Beusen, A.H.W., Dekkers, A.L.M., Bouwman, A.F., Ludwig, W. & Harrison, J. Estimation of global river transport of sediments and associated particulate C, N, and P. *Global Biogeochem Cycles* **19**, 2005GB002453 (2005). 10.1029/2005GB002453
44. Bouwman, A.F.*, et al*. Global trends and uncertainties in terrestrial denitrification and N_2O emissions. *Philosophical Transactions of the Royal Society B: Biological Sciences* **368**, 20130112 (2013). 10.1098/rstb.2013.0112
45. Fowler, D.*, et al*. The global nitrogen cycle in the twenty-first century. *Philosophical Transactions of the Royal Society B: Biological Sciences* **368**, 20130164 (2013). 10.1098/rstb.2013.0164
46. Tubiello, F.N.*, et al*. The FAOSTAT database of greenhouse gas emissions from agriculture. *Environ Res Lett* **8**, 15009 (2013). 10.1088/1748-9326/8/1/015009
47. Paulot, F.*, et al*. Ammonia emissions in the United States, European Union, and China derived by high-resolution inversion of ammonium wet deposition data: Interpretation with a new agricultural emissions inventory (MASAGE_NH3). *Journal of Geophysical Research: Atmospheres* **119**, 4343-4364 (2014). 10.1002/2013JD021130
48. Beusen, A.H.W., Van Beek, L.P.H., Bouwman, A.F., Mogollón, J.M. & Middelburg, J.J. Coupling global models for hydrology and nutrient loading to simulate nitrogen and phosphorus retention in surface water – description of IMAGE–GNM and analysis of performance. *Geosci Model Dev* **8**, 4045-4067 (2015). 10.5194/gmd-8-4045-2015
49. Stohl, A.*, et al*. Evaluating the climate and air quality impacts of short-lived pollutants. *Atmos Chem Phys* **15**, 10529-10566 (2015). 10.5194/acp-15-10529-2015
50. Gütschow, J.*, et al*. The PRIMAP-hist national historical emissions time series. *Earth Syst Sci Data* **8**, 571-603 (2016). 10.5194/essd-8-571-2016
51. Hoesly, R.M.*, et al*. Historical (1750–2014) anthropogenic emissions of reactive gases and aerosols from the Community Emissions Data System (CEDS). *Geosci Model Dev* **11**, 369-408 (2018). 10.5194/gmd-11-369-2018
52. Miyazaki, K.*, et al*. Updated tropospheric chemistry reanalysis and emission estimates, TCR-2, for 2005–2018. *Earth Syst Sci Data* **12**, 2223-2259 (2020). 10.5194/essd-12-2223-2020
53. Chang, J.*, et al*. Reconciling regional nitrogen boundaries with global food security. *Nat Food* **2**, 700-711 (2021). 10.1038/s43016-021-00366-x
54. Minx, J.C.*, et al*. A comprehensive and synthetic dataset for global, regional, and national greenhouse gas emissions by sector 1970–2018 with an extension to 2019. *Earth Syst Sci Data* **13**, 5213-5252 (2021). 10.5194/essd-13-5213-2021
55. Luo, Z.*, et al*. Estimating global ammonia (NH_3) emissions based on IASI observations from 2008 to 2018. *Atmos Chem Phys* **22**, 10375-10388 (2022). 10.5194/acp-22-10375-2022
56. Crippa, M.*, et al*. The HTAP_v3 emission mosaic: merging regional and global monthly emissions

(2000–2018) to support air quality modelling and policies. *Earth Syst Sci Data* **15**, 2667-2694 (2023). 10.5194/essd-15-2667-2023
57. Soulie, A.*, et al*. Global anthropogenic emissions (CAMS-GLOB-ANT) for the Copernicus Atmosphere Monitoring Service simulations of air quality forecasts and reanalyses. *Earth Syst Sci Data* **16**, 2261-2279 (2024). 10.5194/essd-16-2261-2024
58. Climate Watch (2024). https://www.climatewatchdata.org/
59. Tian, H.*, et al*. Global nitrous oxide budget (1980–2020). *Earth Syst Sci Data* **16**, 2543-2604 (2024). 10.5194/essd-16-2543-2024
60. Potsdam Institute for Climate Impact Research (2024). https://www.pik-potsdam.de/en
61. Our World in Data
(2024). https://ourworldindata.org
62. UNFCCC. National Greenhouse Gas Inventory Data (2024). https://unfccc.int/
63. IEA. GHG emissions of all world countries. (Publications Office of the European Union, 2024). https://doi.org/10.2760/4002897
64. Schlesinger, W.H. On the fate of anthropogenic nitrogen. *Proceedings of the National Academy of Sciences* **106**, 203-208 (2009). 10.1073/pnas.0810193105
65. Gu, B., Ju, X., Chang, J., Ge, Y. & Vitousek, P.M. Integrated reactive nitrogen budgets and future trends in China. *Proceedings of the National Academy of Sciences* **112**, 8792-8797 (2015). 10.1073/pnas.1510211112
