# Supplementary material for: Quantifying the role of nitrogen in achieving global Sustainable Development Goals
Source: Nat Commun. 2026 Aug 15;17:9818. doi: 10.1038/s41467-026-76777-w (PMC13575186; doi:10.1038/s41467-026-76777-w)
Supplement: Supplementary file 2 [file 41467_2026_76777_MOESM2_ESM.pdf]

nature portfolio

Peer Review File

Quantifying the role of nitrogen in achieving global sustainable development goals

Corresponding Author: Professor Baojing Gu

This file contains all reviewer reports in order by version, followed by all author rebuttals in order by version.
Parts of this Peer Review File have been redacted as indicated to remove third-party material.

Version 0:

Reviewer comments:

Reviewer #1

(Remarks to the Author)
This study makes a valuable and original contribution to the field by demonstrating that effective nitrogen management, as balancing intake for socio-economic benefits with emission control for environmental protection, is crucial for achieving the SDGs, considering that climate change, nitrogen pressures, and economic growth interact to shape progress. The methodology is sound and there is enough information in the Supplementary Material for the work to be reproduced. The strengths of the work include its comprehensive assessment of nitrogen intake and release dynamics, the clear identification of trade-offs between socio-economic benefits and environmental costs, and the rigorous use of structural equation modeling (SEM) and principal component analysis (PCA) to quantify drivers of SDG progress.

The manuscript is fundamentally suitable for publication following minor revisions. With appropriate clarifications and refinements (as outlined in specific comments below), this work will make a strong contribution to the literature on sustainable nitrogen management.

1. The introduction is somewhat superficial and could present the impact of nitrogen in various contexts related to the SDGs, as well as the challenges of nitrogen governance, in more detail. The references are adequate, but some recent studies are missing. Thus, several papers are being suggested throughout this review, which do not necessarily all need to be cited in the manuscript but may provide additional insights, particularly to contextualize the global nitrogen problem and to better support the discussion of the results.

2. Nitrogen is also important in productive activities related to energy and housing. However, if the authors choose to focus only on its agricultural use (lines 47-48), it is suggested to expand the discussion based on FAO´s Report, 2025 (https://doi.org/10.4060/cd3388en) and the article by SCHULTE-UEBBING et al, 2022 (https://doi.org/10.1038/s41586-022-05158-2).

3. The manuscript could benefit from including an additional discussion on the dual role of nitrogen (lines 51-52) and its direct or indirect relationship with all SDGs, as observed by SUTTON et al., 2019 (https://www.inms.international/nitrogen-grasping-challenge) and CUNHA-ZERI et al., 2022 (https://doi.org/10.1016/j.jenvman.2022.115330).

4. The study's main purpose could be articulated more clearly. Please, explicitly stating the research objectives and their significance in both the abstract and introduction. Additionally, highlighting how the study's findings advance sustainable nitrogen management and inform integrated policy development would strengthen the manuscript. For relevant insights into these aspects, see SUTTON et al., 2022 (https://www.inms.international/nitrogen-opportunities-agriculture-food-environment-unece-guidance-document-integrated-sustainable).

5. Considering lines 70-71, that briefly mention nitrogen governance, it is suggested that the authors develop more about the nitrogen policies and governance. For relevant information about these approaches, see MORSELETTO et al., 2019 (https://doi.org/10.1016/j.gloenvcha.2018.10.010), KANTER et al., 2019, 2020 (https://doi.org/10.1038/s43016-019-0001-5, https://doi.org/10.1038/s41893-020-0577-7), and SANMARTIN, 2024 (https://niche-canada.org/2024/10/21/nitrous-oxide-the-forgotten-super-pollutant/).

6. Line 90 mentions '(...) NOx emissions saw the steepest reduction.' However, was this decline global or regional? This

needs clarification, as data from EDGAR and other sources show that many countries have seen rising NOx and other species of reactive nitrogen emissions over time.

7. The literature cited for lines 118–120 appears inadequate, as the topic is nitrogen reduction, while the referenced paper discusses carbon neutrality in the US. Please provide references that better support this statement on reducing nitrogen emissions.

8. Please provide specific references on nitrogen emissions drivers in Brazil, rather than relying solely on a study about Amazon deforestation and extrapolating to nitrogen (lines 126-127). See, for example, PIRES et al., 2015 (https://doi.org/10.1371/journal.pone.0135234) and CUNHA-ZERI and OMETTO, 2021 (https://doi.org/10.1016/j.envdev.2020.100605).

9. Regarding the claim in lines 221-222 about nitrogen-related constraints being more dominant in low-income countries, please provide supporting literature references to validate this statement and avoid overgeneralization.

10. Regarding the section “Promoting the SDGs from a nitrogen perspective” (lines 236-267), please consider the following questions to strengthen the discussion: (i) The text highlights the need to balance food security, economic growth, and environmental protection but does not explicitly address potential conflicts. Could you briefly discuss key trade-offs (e.g., increased fertilizer use vs. pollution) and how policies might reconcile them? (ii) The recommendation to promote plant-based diets in low-income countries is intriguing but may require further justification. Is there empirical evidence supporting the feasibility and socio-cultural acceptability of such dietary changes in these regions? About dietary changes, see MARTINEZ et al., 2019 (https://doi.org/10.1016/j.envsci.2019.08.022) and LASSALETA et al., 2015 (https://iopscience.iop.org/article/10.1088/1748-9326/11/9/095007).

11. The study's findings, based on 2000-2021 data, predate significant global disruptions including post-pandemic economic shifts, geopolitical conflicts, changing environmental policies – all of which may substantially impact nitrogen-related sustainability and potential integrated policies. Please expand the “Uncertainty and Limitations” section beyond methodological constraints to address how these contextual factors could influence the real-world applicability of the findings to current challenges.

12. Overall, the manuscript is clear and well-written, though a careful revision by a professional language editor is needed (beginning with the title: there should be a 'the' before 'role'...).

Reviewer #2

(Remarks to the Author)
Reviewer Comments
The article was interesting to read and provides a clear overall picture of the role of nitrogen in the Sustainable Development Goals (SDGs). The structure is well-organised and helps readers follow the arguments effectively. However, there are several logical gaps and unaddressed areas that merit further attention. In particular, the discussion would be strengthened by incorporating more relevant citations to support key claims and provide a stronger evidence base. Addressing these points would enhance both the clarity and credibility of the work.

Abstract
• L22: N intake and release by humans or plants?

Introduction
• Emission is used for gases, but other reactive species, like nitrate, can’t be called emissions. Please rephrase this word.
• L52: need a citation for this, suggestion is: https://www.mdpi.com/2504-3129/4/4/28
• L58; give the reason for this
• L58: Discuss the relevant studies available in the literature

Results and discussion
• L78: Mention the period in the text
• L82: When globalisation of the food supply chain start?
• L89: Why it is only aquatic systems?
• L95: No reference is directly related to agriculture and therefore, please use this reference to strengthen this claim: https://www.mdpi.com/2504-3129/4/4/28
• L98-113: The discussion on African countries is lacking. Further, analysis based on the continent is also beneficial to give more information for readers.
• L120: After rigorous restrictions on N emissions, these countries have decreased their emission. However, the emissions of these countries are still above most of the developing countries by many times. This statement has to be included with evidence (citations) to give a correct idea and avoid misunderstanding.
• L121: Include more citations to directly emphasise this claim. Suggestion is here: https://www.sciencedirect.com/science/article/pii/S0048969722080524
• L123: Why are similar countries not included?
• L135: Does this imply that plant-based N ensure food security than animal-based?
• L135-138: Please elaborate on this with suitable reasons

• L145: Not only this, the N input per kg of output is also very high. Please include this with references.
• L147: What kind of unsuitable practices? Discuss with evidence. However, I believe apart from these reasons, other factors are also contributing. Please search for other possible reasons and include them.
• L170: give a citation
• L175 and 181: The authors recommend using meta-analysis to substantiate such claims, as warming and rainfall can exert both positive and negative effects on NUE and nitrogen losses. Therefore, instead of merely describing these effects, it is important to reinforce them with supporting evidence.
• L188: Please change the title to Factors Contributing to the SDGs
• L200: Under this section, please discuss which country or region is mainly influenced by all these three factors
• L221: This N reflects the N used for agriculture or N consumption?
• L217: In Figure 4d, how is temperature a leading factor for high-income countries? Please discuss this in the relevant text.
• L229: Natural resource exploitation is not only due to economic pressure, but the invasion of developed countries is also a main reason for this
• L243: Apart from these, strong policy and strict implementation of policies are essential for countries to have ample access to N fertilisers
• L246: This reactive approach is not advisable. It is more sensible to focus on minimising nitrogen pollution rather than strengthening public health measures targeting it.
• L247: This is already in practice in developing countries, but this suggestion should be given to the developed countries
• L257: What are these N recycling systems?

Method
• L291: reference for this model?
• L320: Give proper citation for the equations used in this study

Supplementary document
• What is the source for the supplementary Figure 2?
• Supplementary Figure 4: The Figure caption does not fully describe the legend of the figure.

Reviewer #3

(Remarks to the Author)
Key results
The key message covers the overarching patterns to N trends and how these interact with SDG and overall social, economic, and environmental trends. Here, there are some synergies but often trade-offs between these goals, especially the social and economic goals with the environment. The trends vary according to country and regional contexts. It was interesting to see that there were positive and negative implications of plant-based and meat-based diet trends across different regions.

Validity
Noting at the outset that I am not fully familiar with these models or the details of the analysis. That said, most of the analysis appears valid. The only area I find somewhat uncertain is the section on nitrogen contribution quantification, particularly the conclusions in lines 175–186.
It may be worth unpacking further how nitrogen also affects climate change, as the patterns of influence are not straightforward. Certain nitrogen compounds can have either warming or cooling effects, which complicates interpretation. If Nr waste influences climate change, and this study also aims to assess how climate change affects nitrogen intake and release, then it would be helpful to provide a clearer explanation of these reciprocal relationships.

Significance
This feels like a significant paper, and whilst some of the patterns and categories are fairly crude, that is often the case given the data available at this scale. There are still important trends and drawing on the SDG links to N and quantifying is important to evidence how N has implications for the all the SDGs in both positive and negative ways. This study provides a unique approach and findings in that regard.

Data and methodology
The data, from what I understand, feels robust. However, it feels the paper could be more explicit about some of the caveats of the different data sets available beyond what is already there. Such as the variations in quality and data sources across certain countries and regions.
Also, this may be the journal style but for the Figures 1 and others their source data was not explicitly stated in the figrues. Then also is not clear what the base map was applied without endorsement using data from the GADM (https://gadm.org/)was applied? Was this to access data or create the map with admin boundaries? When you follow the link, it feels like it takes you to a slightly spurious webpage. So, suggest there is more explanation there of what GADM was used for and with further explanation of what datasets were applied in each figure.
Also, would it be a requirement to for to share the raw database? Current tables include the figures and summary tables but not the datasets used to make the calculations, recognising that the data comes from secondary sources.
Likewise, the fact that per capita was only used to standardise across the countries could be worth acknowledging that there would be different patterns observed if you were to look at crop land per ha, or absolute Nr values.

Analytical approach
The analytical approach appears to be strong with a number of comprehensive analyses applied. However, the overall approach is outside the scope of my expertise.

Suggested improvements
All the figures include the figures in the supplementary data were fairly difficult to read and too small, so recommend enlarging these. Also see above for other suggestions

Clarity and context
Recommend that intake and release are explained more clearly in the introduction. Also, the SDG index is not really well explained, nor what this data includes, so recommend addressing this in the text.

References
The literature appear to have been referenced appropriately.

Reviewer #4

(Remarks to the Author)
General comments
The paper addresses an important question, that of the dual role of nitrogen availability in agriculture as a key resource for food production and a source of environmental degradation through environmental loss. The paper states that few studies have addressed this issue as a cross-cutting theme of SDG. However, there is a vast literature on nitrogen use in agriculture that simultaneously assesses nitrogen-related food security and environmental impact. It is unclear what the new insights of the paper are on this theme. To this regard, a substantial discussion of the results obtained with the literature is missing. Moreover, the system boundary considered in the paper is also unclear as well as the internal system flows that are addressed. In addition, how the different models used are integrated in a consistent modelling framework? Although some description is provided on the CHANS balance model, the other models used are not described, nor the integration between them.
The paper does not state neither the research hypothesis and assumptions on model parameters. More generally, the way the method and results are presented makes understanding the study very difficult. Authors should explain more thoroughly the methods: be more explicit about which data are used for which part of the model / analysis; be more explicit about what type of results are expected at each step of the method; in order for the reader to more easily identify how the results were obtained. The evidence used to calibrate the model and validate the modelling results is unclear. Furthermore, the paper employs path coefficients—a statistical measure used in structural equation modelling to quantify the strength and direction of relationships between variables. However, as far as I understand, it does not precisely define and justify their application in this context.
In addition, the way the nitrogen cascade (the sequential transformation of different reactive nitrogen compounds) is taken into account (if it is) in the modelling framework is not explained neither. The paper does not at all mention the nitrogen cascade although it targets linking specific reactive nitrogen compounds into a negative impact SDG framework.
Also, the paper should be clearer in the results. Are the relations between the exposed variables correlations or actual causalities. Does the method allow to demonstrate causality and how?
Another concern arises from the paper's presentation of percentages reflecting the contribution of various factors to the advancement of SDG. It is unclear how different SDGs, which encompass diverse and sometimes incommensurable objectives, are rendered comparable or hierarchized. I did not find any robust comparability framework for assessing the relative contributions of factors to SDG. Additionally, the paper does not elucidate how these percentages correlate with macroeconomic features such as urbanization or infrastructure development, leaving a critical gap in understanding the systemic drivers of SDG outcomes. Another weakness is the absence of considerations on the interactions and combined impacts from Nitrogen and other biogeochemical elements, like phosphorus, carbon etc which together generate combined and cumulated impacts (cocktail effect). Consequently, the paper does not address the complexity and interdependence of global challenges in analysis of SDG scores. Moreover, it does not address how interlinked factors—such as climate change, economic inequality, and resource depletion—are integrated into the analysis.
The section to promote the SDGs seems insufficient. Of course, policy recommendations should depend on the regional and economic context, which is mentioned in the article. But this section should explain why the analysis allows to conclude on specific policy recommendations, compare them to usual recommendations from the literature, and discuss differences.
The structure and clarity of the paper is also further compromised by the proliferation of numerical values within the text instead of results analysis. Moreover, the paper does not position its findings within the broader literature on food production capacity, nitrogen loss, impacts and SDG accounting.

Paragraph-specific comments
Abstract
l.52: SDG 2 is justified above, but "poverty alleviation" is not justified, do you have a reference for this?
Introduction

The early citation of Supplementary Figure 1 in the introduction (line 5 of the introduction!) lacks contextual explanation, leaving readers to infer its relevance without guidance.

Results and discussion

Nitrogen Intake and Release: this section could almost be part of the introduction, as it is a primary data analysis from FAOStat, illustrated straightforward in a world map. This is a good introductory image to the topic, but is not the result of the study, which focuses of the impact of these numbers on the SDG achievement. You should be more explicit which data are directly drawn from FAOSTAT and which data were obtained after using CHANS model. Even if it was explained in the Methods section, it should be more explicit when presenting the results.

l.77: even if it is said in the method section afterwards that data is from FAOStat, it would be helpful to remind quickly here which data allows to dray supplementary figure 2a. Strange data break in 2010, could be interesting to try to explain it.

l.87: same here, what data is used for figure 2b ? Indicate it shortly on the figure or in the text. (probably data output from the CHANS model)

l.116: maybe a comment on the absolute value of nitrogen pollution in high income countries could be relevant ? Even if some reductions were achieved, the levels of nitrogen release are still high.

Driving Pathways:

l.158-186: several concepts are confusing in the analysis:

- First, you present SDG index as determined by a combination of independent factors (Nitrogen, social and natural). However Nitrogen variables are then presented as dependent on social and natural variables – which is understandable – but this is in contradiction with these variables being independent in the first place.
- Second, you present the relations between all variables as causality (e.g. l.160: “exerts a strong positive direct effect”), but does the study highlight correlations or actual causality effects ? (earlier l.139: “correlates positively”). Some clarification would be welcome on that point.

Contribution to the SDGs:

l.195: same as above, it should be very clear if the PCA analysis highlights correlations between the studied variables, or if you really speak of causality here.

l.217: a suggestion here, fig. 4d might be easier to read if the bar chart was regrouped per income class instead of per drivers.

In the limits of the approach, the following questions could be treated more deeply:

- Is the SDG index entirely captured by the three dimensions Nitrogen/Social/Natural, and which critical dimensions could better explain the observed trends ? (e.g. energy, natural resources, national policies and international trades/geopolitical relationships)
- Are the Social/Natural dimensions correctly captured by the selected indicators (GDP, urbanization, MAT, MAP) and would the results vary significantly when selecting different indicators ? (e.g. education, employment, or extreme weather events occurrence)

Promoting the SDGs from a nitrogen perspective:

The policy recommendations derived from the study seem overly simple in this paragraph and miss some primordial policy recommendations. Also, there are always risks associated with certain policies, which should be mentioned as well. Of course, these recommendations should be tailored to the economic and regional context of the area as done in the paper. But from current literature, the following recommendations are relevant (see examples below), and it could be interesting to explain whether your analysis justifies similar conclusions or not. For example, why does the analysis not mention management of organic fertilizer sources ?

Examples of policy recommendations:

Low-Income Countries:

Increase access to affordable fertilizers and improve nitrogen use efficiency (NUE) through education and extension services. Promote site-specific nutrient management and recycling of organic sources (e.g., manure). Support smallholder farmers with knowledge-based management and locally adapted practices.

Risks: Over-application as economies grow can quickly lead to pollution if not managed.

Middle-Income Countries

Implement integrated nutrient management, combining synthetic and organic sources. Remove blanket fertilizer subsidies and instead incentivize efficiency improvements and recycling. Invest in technologies like enhanced-efficiency fertilizers and precision application. Develop compensation mechanisms for smallholder farmers affected by policy changes (e.g., fertilizer taxes).

Risks: Policy changes can impact food prices and farmer livelihoods if not carefully designed.

High-Income Countries

Tighten regulations on fertilizer use, promote extensification, and internalize environmental costs in food prices. Encourage dietary shifts (less animal protein), food waste reduction, and circular nutrient flows. Support agroecological and organic practices, and invest in advanced technologies for NUE.

Risks: Overly strict policies may reduce yields or farm income if not balanced with market incentives.

(references: Zhang X. – Nature – 2015 (528[7580]) ; Wang X. – Sustainability Science – 2023 (18[3]) ; You L. – Nature Communications – 2023 (14[1]) ; Stevens C.J. – Science – 2019 (363[6427]) ; Guo Y. – Nature Food – 2020 (1[10]) ; Billen G. – One Earth – 2021 (4[6]) ; Cui Z. – Nature – 2018 (555[7696]) ; Xia L. – Global Change Biology – 2017 (23[5]) ; Maaz T.M. – Global Change Biology – 2021 (27[8]) ; Leip A. – Global Food Security – 2020 (28) ; van Grinsven H.J.M. – Environmental Research Letters – 2015 (10[2]) ; Gu B. – Environmental Science & Technology – 2019 (53[15])

L.246-247: maybe the first step to mitigate the impact of nitrogen pollution is to mitigate the pollution itself with nitrogen management strategies for better NUE, before health caretaking (which is of course important too).

Methods
l.271: what do you mean by “indicator” ? unclear formulation
l.276: Supplementary tables 6 and 7 are not necessarily needed as plant-based and animal-based food categories are already presented in the FAOStat database.

Data sources : it would be good to have precise links or DOIs to have easy and direct access to the data used for this study.

National Scale Nitrogen Budget: the 3d paragraph about data sources could be combined with the previous “Data Sources” section, in a clearer comprehensive section. It is unclear overall which data sources are used to feed which section of the CHANS model. It would be appreciated to have a clear DOI for each data source, associated with the specific use made of these data sources.
l.314-318: can you add the references to these “Recently published field experiments, […] inventories” ?

Statistical analysis: overall, it is very hard to understand the work described in this section: many analysis and models are described, but it is not completely explicit what data intake is used, what is the exact goal of the model. There is a clear gap in the definition of the terms and a lack of clarity in the explanation of the approach and goal for each step of this study.
l.322: I find no definition of the SDG index, what does it relate to ? I imagine it relates to data presented here: SDG Index, but it should be more explicit in the paper.
l.325: which exact panel data are you referring to ?
l.349: which background and knowledge are you referring to ?

References
Include DOI or links for easier recovery of references

Reviewer #5

(Remarks to the Author)
I co-reviewed this manuscript with one of the reviewers who provided the listed reports. This is part of the Nature Communications initiative to facilitate training in peer review and to provide appropriate recognition for Early Career Researchers who co-review manuscripts.

Version 1:

Reviewer comments:

Reviewer #1

(Remarks to the Author)
I have reviewed the revised manuscript and the authors' responses to the reviewers' comments. All my previous concerns and suggestions have been satisfactorily addressed, leading to a significantly improved manuscript. I have no further comments and recommend the manuscript for publication.

Reviewer #2

(Remarks to the Author)
Most of the comments are addressed sufficiently.

Reviewer #3

(Remarks to the Author)
I am satisfied that all my comments have been adequately addressed and would recommend acceptance of this manuscript.

Reviewer #6

(Remarks to the Author)
The manuscript “Quantifying the role of nitrogen in achieving global sustainable development goals” addresses a highly ambitious and interdisciplinary question. Despite the inherent complexity of linking nitrogen dynamics with broad SDG performance, including social dimensions that are not commonly integrated into nitrogen-related studies, I consider this work to be potentially valuable and conceptually interesting. In particular, the manuscript provides a broad systems-level perspective that could stimulate future research on the role of nitrogen management in sustainable development pathways.

Overall, I agree with most of the concerns raised by reviewer #4. In my opinion, reviewer #4 identified several important methodological and conceptual issues that required clarification, particularly regarding the definition of system boundaries,

the integration of the CHANS framework with the statistical analyses, the distinction between causality and correlation, and the transparency of the modelling approach. I also believe that the authors have substantially improved the manuscript in response to these comments. The revised version is considerably clearer in terms of methodological traceability, model description, and interpretation of the statistical analyses.

There are only a few points raised by reviewer #4 with which I partially disagree. First, regarding the concern about whether the "Social" and "Natural" dimensions are adequately represented by the selected indicators (GDP, urbanization, MAT, and MAP), I consider the authors' response reasonable within the scope of the study. Although alternative indicators could certainly have been included, the authors adequately justify their selection criteria based on analytical parsimony, comparability, and reduction of collinearity among explanatory variables.

Second, reviewer #4 questioned the novelty of the manuscript, arguing that there is already extensive literature linking nitrogen use, food security, and environmental degradation. While I agree that this literature is indeed extensive, I believe the novelty of the present study lies in its attempt to integrate nitrogen intake and release dynamics within the broader framework of all 17 SDGs, rather than focusing only on SDGs directly associated with food production or environmental pollution (e.g., SDGs 2, 6, 12, 13, 14, and 15). In this regard, I believe the revised manuscript now better contextualizes its contribution relative to previous studies.

I also agree that reviewer #4 raised an important conceptual limitation regarding the exclusion of interactions with other biogeochemical elements such as phosphorus and carbon. This is indeed one of the first questions that arises when reading the manuscript. However, although this limitation remains relevant, I consider the authors' decision to maintain a nitrogen-centered framework acceptable within the scope of the study, particularly after the expanded discussion included in the revised limitations section.

Importantly, the comments raised by reviewer #4 did not reduce my perception of the conceptual relevance or novelty of the manuscript. On the contrary, I believe the revised version is substantially stronger because the review process improved the transparency of the data sources, clarified the modelling framework, and moderated several interpretations that were initially presented too strongly. Overall, I consider reviewer #4's comments constructive, scientifically justified, and highly valuable for improving the manuscript.

I also do not consider the additional analyses and revisions requested by reviewer #4 to be unfeasible or beyond the scope of the study. In fact, if I had reviewed the original version of the manuscript, I would likely have raised several similar concerns.

Beyond these points, I would like to add a few additional comments regarding the current structure and interpretation of the manuscript.

1. First, I agree with reviewer #4 that Figures 1 and 2 remain primarily descriptive. Although the data are normalized on a per-capita basis, these figures still appear to represent a direct visualization of underlying database trends rather than central analytical outcomes derived from the modelling framework itself. In my opinion, they function more as contextual information introducing the topic than as core results of the study. More specifically, Figures 1 and 2 appear closely connected to the information already presented in Supplementary Figure 2a and 2b, essentially reflecting a comparison between the years 2000 and 2022, although Supplementary Figure 2b additionally provides information regarding the forms of nitrogen losses. For this reason, I feel that the main figures do not substantially extend the analytical interpretation beyond the temporal trends already shown in the Supplementary Information. I believe these figures could potentially be condensed, incorporated more explicitly as introductory/background material, or partially transferred to the Supplementary Information in order to improve the balance between descriptive context and analytical results in the main manuscript.

2. Second, I found the transition between the "Driving pathways" and "Factors contributing to the SDGs" sections somewhat inconsistent in terms of the grouping strategy used for countries. In the "Driving pathways" section, the analyses and discussion are primarily organized by world regions, whereas in Figure 4d and in the policy-oriented section "Promoting the SDGs from a nitrogen perspective", the interpretation shifts toward country classification by income group. In my opinion, this creates a degree of conceptual inconsistency throughout the manuscript.

I believe this issue is particularly important because several results presented in the manuscript seem to support the idea that socio-economic development level may ultimately explain SDG-related nitrogen dynamics more consistently than broad geographical classification alone. For example, in the SEM presented in Figure 3, the relationships between socio-economic variables (particularly GDP and urbanization) and nitrogen intake/release appear substantially stronger than those associated with natural variables such as MAT and MAP. Although climatic differences clearly remain important, this pattern may suggest that the economic development level of a country exerts a stronger influence on how nitrogen dynamics interact with SDG performance.

This interpretation also appears to be consistent with the contribution analysis shown in Figure 4d. While the contribution of nitrogen intake remains relatively similar across the four income groups, the contribution of nitrogen release progressively decreases with increasing income level. This is, in my opinion, one of the most interesting results of the manuscript, because it may indicate that as countries improve the control and management of reactive nitrogen losses, the relative influence of nitrogen release on SDG performance diminishes, while other drivers, particularly climate-related factors, become proportionally more important. Interestingly, this interpretation is also consistent with the discussion already included in the revised manuscript regarding high-income countries (lines 275 to 280 in the revised version).

Furthermore, the regions showing the highest contribution percentages of nitrogen-related drivers in Figure 4a largely overlap with countries classified as low- or lower-middle-income. Similarly, Figures 2g,h,i show that the strongest positive trends in nitrogen release variation are concentrated in many of these same countries. Therefore, the income-level perspective already appears implicitly throughout multiple sections of the manuscript, including the final policy recommendations section, which is itself structured according to income categories.

For these reasons, I believe the manuscript could benefit from a more consistent analytical framing throughout the different sections, either by maintaining the regional approach more systematically or, alternatively, by strengthening the interpretation based on income-group classification across the manuscript.

3. Finally, regarding the policy recommendations section, I think the revised version has improved substantially after reviewer #4's comments. However, I believe it would still be valuable to emphasize more strongly the importance of farmer education and technical support in low- and lower-middle-income countries, particularly regarding nitrogen loss mitigation and fertilizer overapplication practices. In many agricultural systems, excessive fertilizer application is still used as a strategy to "ensure yield security," despite its environmental consequences.

I would also suggest some caution regarding the promotion of organic alternatives without discussing their potential trade-offs (line 322 in the revised version). Depending on their C:N ratio and management conditions, some organic amendments may stimulate denitrification processes and increase greenhouse gas emissions. Therefore, the manuscript could perhaps mention that improvements in nitrogen use efficiency may also involve enhanced-efficiency fertilizers or the use of crops with biological nitrification or denitrification inhibition capacity (BNI/BDI systems), which represent promising complementary approaches for reducing reactive nitrogen losses.

Reviewer #7

(Remarks to the Author)

Version 2:

Reviewer comments:

Reviewer #6

(Remarks to the Author)
After reviewing the manuscript, I believe that all my comments have been adequately addressed and I recommend the acceptance of this work.

Open Access This Peer Review File is licensed under a Creative Commons Attribution 4.0 International License, which permits use, sharing, adaptation, distribution and reproduction in any medium or format, as long as you give appropriate

credit to the original author(s) and the source, provide a link to the Creative Commons license, and indicate if changes were made.
In cases where reviewers are anonymous, credit should be given to 'Anonymous Referee' and the source.
The images or other third party material in this Peer Review File are included in the article's Creative Commons license, unless indicated otherwise in a credit line to the material. If material is not included in the article's Creative Commons license and your intended use is not permitted by statutory regulation or exceeds the permitted use, you will need to obtain permission directly from the copyright holder.
To view a copy of this license, visit https://creativecommons.org/licenses/by/4.0/

Point-to-point response to reviewers

Reviewer #1:

**Reviewer comment**	**Number**, Response and Change
This study makes a valuable and original contribution to the field by demonstrating that effective nitrogen management, as balancing intake for socio-economic benefits with emission control for environmental protection, is crucial for achieving the SDGs, considering that climate change, nitrogen pressures, and economic growth interact to shape progress. The methodology is sound and there is enough information in the Supplementary Material for the work to be reproduced. The strengths of the work include its comprehensive assessment of nitrogen intake and release dynamics, the clear identification of trade-offs between socio-economic benefits and environmental costs, and the rigorous use of structural equation modeling (SEM) and principal component analysis (PCA) to quantify drivers of SDG progress.<br><br>The manuscript is fundamentally suitable for publication following minor revisions. With appropriate clarifications and refinements (as outlined in specific comments below), this work will make a strong contribution to the literature on sustainable nitrogen management.	**R1-1:** Thank you for your recognition of our study. We have carefully reviewed your comments and have made clarifications and improvements in response, hoping to address your concerns.
1. The introduction is somewhat superficial and could present the impact of nitrogen in various contexts related to the SDGs, as well as the challenges of nitrogen governance, in more detail. The references are adequate, but	**R1-2:** Thank you for your suggestions. We have optimized the discussion of nitrogen's impact and challenges in various SDG contexts in the Introduction. The revised section is as follows:<br><br>*"Nitrogen is a critical driver of global sustainability, influencing a broad range of SDGs through both direct and indirect pathways. As an essential nutrient for food production, nitrogen supports the diets of*

some recent studies are missing. Thus, several papers are being suggested throughout this review, which do not necessarily all need to be cited in the manuscript but may provide additional insights, particularly to contextualize the global nitrogen problem and to better support the discussion of the results.	*nearly half of the global population, playing a pivotal role in alleviating poverty (SDG 1) and ensuring food security (SDG 2)*[1, 2]. *However, its losses in agriculture and industrial production generates multiple environmental externalities*[3]. *In 2022, global nitrogen losses reached 142 million tonnes (Tg), more than three times the safe planetary boundary of 43 Tg*[4], *exacerbating threats to ecosystem stability (SDGs 14 and 15) and human health (SDGs 3 and 6). Furthermore, emission of nitrous oxide (N_2O), with a global warming potential approximate 300 times that of carbon dioxide (CO_2), also leads to global climate change (SDG 13)*[5, 6]. *Thus, sustainable production and consumption (SDG 12) are intrinsically linked to effective nitrogen management to promote its production function while mitigate its environmental and climate impacts*[7]. *Considering the varying sensitivity of ecosystems and the differing levels of development across countries, regional equity and international cooperation justice are also impacted by nitrogen pollution (SDGs 10, 16, and 17)*[8]*.”*<br><br>In addition, based on the recent literature you provided, we have added further background discussion and clarified the research objectives and significance in the introduction, as detailed in **R1-3 to R1-6**.
2. Nitrogen is also important in productive activities related to energy and housing. However, if the authors choose to focus only on its agricultural use (lines 47-48), it is suggested to expand the discussion based on FAO´s Report, 2025 (https://doi.org/10.4060/cd3388en) and the article by SCHULTE-UEBBING et al, 2022 (https://doi.org/10.1038/s41586-022-05158-2).	**R1-3:** Thank you for the suggestion. We have included the role of nitrogen in industrial production and have also added the FAO report you provided as a citation. The revision is as follows:<br><br>*Line 47: “…However, its losses in agriculture and industrial production generates multiple environmental externalities*[3]*…”*<br><br>*Line 60: “To meet the nutritional needs of the global population without exceeding its safe planetary boundary, it is essential to consider the diversity of agricultural systems and the broader context of non-agricultural nitrogen releases. Agricultural food systems remain the primary drivers of nitrogen cycle disruptions at national, regional, and global scales*[9]. *Therefore, ensuring the sustainable use of nitrogen necessitates measures that improve nitrogen use efficiency (NUE) while minimizing environmental impacts…”*
3. The manuscript could benefit from including an additional discussion on the dual role of nitrogen (lines 51-52) and its direct or indirect relationship with all SDGs, as observed by SUTTON et al., 2019 (https://www.inms.international/nitrogen-grasping-challenge) and CUNHA-ZERI et al., 2022 (https://doi.org/10.1016/j.jenvman.2022.115330).	**R1-4:** Thank you for your suggestion. We have elaborated on the dual role of nitrogen in more detail and discussed its connections with as many SDGs as possible. We have also incorporated the references you suggested. The revision is as detailed in **R1-2**.
4. The study's main purpose could be articulated more clearly. Please, explicitly stating the research objectives and their	**R1-5:** Thank you for your suggestion. We have clarified the research objectives and significance in the *Introduction* and emphasized that the findings of this study can provide reliable evidence for promoting sustainable nitrogen management. We have also added the references

significance in both the abstract and introduction. Additionally, highlighting how the study's findings advance sustainable nitrogen management and inform integrated policy development would strengthen the manuscript. For relevant insights into these aspects, see SUTTON et al., 2022 (https://www.inms.international/nitrogen-opportunities-agriculture-food-environment-unece-guidance-document-integrated-sustainable).	you recommended. The revision is as follows:<br><br>*"This study addresses this knowledge gap by integrating a global Coupled Human And Natural Systems (CHANS) model with structural equation modeling (SEM) and principal component analysis (PCA). We assess the influence of nitrogen on SDG progress across 166 countries from 2000 to 2022. Specifically, the study (1) examines temporal trends in per capita nitrogen intake from food systems, including both plant based and animal based consumption, and total nitrogen releases, including emissions to air and losses to water, (2) quantifies the effects of nitrogen related drivers on the SDG index, a composite indicator of national performance across the 17 SDGs calculated as the average of individual SDG scores, (3) explores interactions among nitrogen dynamics, socioeconomic factors, and environmental conditions, and (4) evaluates the relative contributions of these drivers to SDG performance. By embedding nitrogen flows within broader socioeconomic and climatic contexts, this study provides a comprehensive assessment of nitrogen's role in shaping global sustainability. The findings offer actionable insights for improving nitrogen management and for supporting integrated policies aligned with the 2030 sustainable development agenda."*
5. Considering lines 70-71, that briefly mention nitrogen governance, it is suggested that the authors develop more about the nitrogen policies and governance. For relevant information about these approaches, see MORSELETTO et al., 2019 (https://doi.org/10.1016/j.gloenvcha.2018.10.010), KANTER et al., 2019, 2020 (https://doi.org/10.1038/s43016-019-0001-5, https://doi.org/10.1038/s41893-020-0577-7), and SANMARTIN, 2024 (https://niche-canada.org/2024/10/21/nitrous-oxide-the-forgotten-super-pollutant/).	**R1-6:** Thank you for your suggestion. Based on the literature you recommended, we have expanded the discussion on nitrogen policies and governance mechanisms. The revision is as follows:<br><br>*Line 65: "...Despite origin-based solutions may offer more specific and impactful results, policymakers still have a wide range of options available to address nitrogen pollution across the whole food production and consumption chain[10, 11]. Integrated management across the whole chain and aligning policies across different environmental domains are critical to avoiding the risk of substituting one form of nitrogen pollution for another[12]..."*
6. Line 90 mentions '(...) NOx emissions saw the steepest reduction.' However, was this decline global or regional? This needs clarification, as data from EDGAR and other sources show that many countries have seen rising NOx and other species of reactive nitrogen emissions over time.	**R1-7:** Thank you for your suggestion. We have refined the expression in this section by including the scope of NOx emissions reduction and the concept of per capita releases, hoping to address your concerns. The revision is as follows:<br><br>*Line 112: "...Among these, global average per capita NO_x emissions saw the steepest reduction, falling from 4.9 to 3.8 kg nitrogen, representing a 22% decrease..."*
7. The literature cited for lines	**R1-8:** Thank you for your suggestion. We have replaced the reference

118–120 appears inadequate, as the topic is nitrogen reduction, while the referenced paper discusses carbon neutrality in the US. Please provide references that better support this statement on reducing nitrogen emissions.	at *Line 147* with the new one. The updated reference is as follows:<br><br>*"Sobota, D.J., Compton, J.E., McCrackin, M.L. & Singh, S. Cost of reactive nitrogen release from human activities to the environment in the United States. Environ Res Lett **10**, 25006 (2015). 10.1088/1748-9326/10/2/025006"*
8. Please provide specific references on nitrogen emissions drivers in Brazil, rather than relying solely on a study about Amazon deforestation and extrapolating to nitrogen (lines 126-127). See, for example, PIRES et al., 2015 (https://doi.org/10.1371/journal.pone.0135234) and CUNHA-ZERI and OMETTO, 2021 (https://doi.org/10.1016/j.envdev.2020.100605).	**R1-9:** Thank you for your suggestion. We have optimized the citation at *Line 157* based on the reference you provided. The updated citation is as follows:<br><br>*"Cunha-Zeri, G. & Ometto, J. Nitrogen emissions in Latin America: A conceptual framework of drivers, impacts, and policy responses. Environ Dev **38**, 100605 (2021). 10.1016/j.envdev.2020.100605"*
9. Regarding the claim in lines 221-222 about nitrogen-related constraints being more dominant in low-income countries, please provide supporting literature references to validate this statement and avoid overgeneralization.	**R1-10:** Thank you for your suggestion. We have added the reference at Line 285 as recommended. The added citation is as follows:<br><br>*"Turner, C., et al. Food Environment Research in Low- and Middle-Income Countries: A Systematic Scoping Review. Adv Nutr **11**, 387-397 (2020). 10.1093/advances/nmz031"*
10. Regarding the section "Promoting the SDGs from a nitrogen perspective" (lines 236-267), please consider the following questions to strengthen the discussion: (i) The text highlights the need to balance food security, economic growth, and environmental protection but does not explicitly address potential conflicts. Could you briefly discuss key trade-offs (e.g., increased fertilizer use vs. pollution) and how policies might reconcile them? (ii) The recommendation to promote plant-based diets in low-income countries is intriguing but may require further justification. Is there empirical evidence supporting the feasibility and socio-cultural acceptability of such dietary changes in these regions? About dietary changes, see MARTINEZ et al., 2019	**R1-11:** Thank you for your suggestion. We have systematically revised the discussion in the section *"Promoting the SDGs from a Nitrogen Perspective."* The revisions mainly include: 1) A discussion of the potential conflict risks faced by regions with different income levels and the trade-offs and reconciliations associated with nitrogen management; and 2) Based on the reference you provided, we have shifted the discussion on promoting plant-based diets to the recommendations for high-income countries. The revised section is as follows:<br><br>*"Achieving the SDGs, particularly with regard to nitrogen management, requires tailored strategies that consider the specific environmental conditions and developmental stages of each region. Given the varying contributions of nitrogen, socioeconomic factors, and climate to SDG progress across different areas, it is essential to adopt region-specific approaches. A one-size-fits-all policy is inadequate to address the diverse challenges associated with nitrogen use in different contexts.*<br><br>*In low-income countries, where nitrogen intake and release have the most significant impact on SDG progress, improving access to fertilizers is crucial, as nitrogen management plays a key role in enhancing agricultural productivity. Expanding fertilizer availability and promoting NUE through precision agriculture and education are essential for improving food security and reducing malnutrition[13]. Additionally, by gaining insights into farmers' needs and preferences, governments can implement systematic policy interventions to improve*

(https://doi.org/10.1016/j.envsci.2019.08.022) and LASSALETA et al., 2015 (https://iopscience.iop.org/article/10.1088/1748-9326/11/9/095007).	*fertilizer accessibility[14]. This is a key strategy for enhancing agricultural productivity, increasing farmers' incomes, and ultimately reducing poverty levels. International support is also vital for addressing infrastructure gaps, while sustainable agricultural practices, such as the recycling of organic resources, can foster both environmental sustainability and poverty alleviation[15]. Although economic growth may present challenges, careful monitoring and regulation of fertilizer use are necessary to prevent unintended environmental damage.*<br><br>*In middle-income countries, where the contributions of various factors to SDG progress are relatively balanced, the impact of nitrogen release is more prominent in lower-middle-income countries. The primary challenge lies in balancing economic growth with environmental sustainability, particularly in regions dependent on intensive agriculture. These countries should focus on modernizing agricultural systems by integrating nutrient management strategies that combine synthetic fertilizers with organic alternatives[16]. Implementing pollution control frameworks, such as the European Union's National Emissions Ceilings Directive[17], can mitigate nitrogen-related environmental harm. When designing policies, it is critical to ensure that smallholder farmers are not adversely affected. Providing compensation mechanisms and incentives to enhance fertilizer use efficiency is key to protecting vulnerable communities from the negative consequences of nitrogen pollution[18].*<br><br>*In high-income countries, where nitrogen intake from animal-based sources plays a more prominent role, there is an opportunity to internalize agricultural externalities into food prices. This approach can encourage sustainable farming practices and dietary changes, such as reducing animal protein consumption[19, 20]. With access to advanced technologies and financial resources, these regions are well-positioned to enforce stringent nitrogen emission regulations. Investments in advancing NUE technologies, organic farming, and ecological practices can further reduce nitrogen's environmental footprint[20]. However, it is crucial that policies also support agricultural productivity and farmers' incomes, ensuring a balance between environmental goals and the economic realities of agriculture.*<br><br>*Effective nitrogen management is central to advancing the SDGs. By improving NUE across the whole chain, mitigating emissions, and addressing the environmental and economic impacts of nitrogen pollution, countries can make significant progress toward achieving these goals[21]. However, managing potential risks and ensuring that policies benefit all sectors, particularly vulnerable communities, is essential. International cooperation and knowledge sharing are crucial to supporting these efforts, making nitrogen management a key component of global sustainability. A balanced approach that addresses environmental, social, and economic needs will contribute to a more sustainable and equitable future for all."*
11. The study's findings, based on 2000-2021 data, predate	**R1-12:** Thank you for pointing out the limitations regarding the selection of the study period. Based on your suggestion, we have

significant global disruptions including post-pandemic economic shifts, geopolitical conflicts, changing environmental policies – all of which may substantially impact nitrogen-related sustainability and potential integrated policies. Please expand the "Uncertainty and Limitations" section beyond methodological constraints to address how these contextual factors could influence the real-world applicability of the findings to current challenges.	enhanced the discussion in the *"Uncertainty and limitations"* section. The revised section is as follows:<br><br>*"Second, the study covers the period from 2000 to 2022, and does not account for the impacts of major global disruption events that occurred after this period. These events include the post-pandemic economic transformation, the intensification of geopolitical conflicts, and adjustments to environmental policies across various countries. All of these factors may have affected global nitrogen flow patterns, agricultural and industrial nitrogen management strategies, and pathways to achieving sustainability goals. Therefore, the conclusions drawn from historical data may have limited applicability in current and future contexts. However, this focus allows for a more focused examination of the role and contribution of nitrogen, excluding the complex influence of international politics. In the face of rapidly changing policy environments and global cooperation frameworks, the relevant conclusions and recommendations should be carefully reassessed and adjusted in light of emerging dynamics."*
12. Overall, the manuscript is clear and well-written, though a careful revision by a professional language editor is needed (beginning with the title: there should be a 'the' before 'role'...).	**R1-13:** Thank you for your suggestion. Throughout the revision process, we have paid special attention to enhancing the language professionalism and structural rigor of the manuscript, with the aim of improving its overall academic readability. Additionally, we have revised the title, as per your suggestion, to: *"Quantifying the role of nitrogen in achieving global sustainable development goals."*

Reviewer #2:

Reviewer comment	**Number**, Response and Change
The article was interesting to read and provides a clear overall picture of the role of nitrogen in the Sustainable Development Goals (SDGs). The structure is well-organized and helps readers follow the arguments effectively. However, there are several logical gaps and unaddressed areas that merit further attention. In particular, the discussion would be strengthened by incorporating more relevant citations to support key claims and provide a stronger evidence base. Addressing these points would enhance both the clarity and credibility of the work.	**R2-1:** Thank you for your positive evaluation of our study. Following your valuable suggestions, we have revised the manuscript substantially, with a particular focus on addressing logical gaps and expanding the discussion on previously underexplored areas. We have also added supporting references where necessary to strengthen key arguments. We hope that the revised manuscript improves in clarity and credibility.
(Abstract)<br>• L22: N intake and release by humans or plants?	**R2-2:** Thank you for your suggestion. We have added the following clarification to the abstract at *Line 22*: *"…nitrogen intake and release by humans have influenced…"*
(Introduction)<br>• Emission is used for gases, but other reactive species, like nitrate, can't be called emissions. Please rephrase this word.	**R2-3:** Thank you for your suggestion. We have carefully reviewed all instances of the term "emission" throughout the manuscript and, depending on the specific context, replaced it with more precise expressions such as *"nitrogen releases"* and *"nitrogen losses"*.
(Introduction)<br>• L52: need a citation for this, suggestion is: https://www.mdpi.com/2504-3129/4/4/28	**R2-4:** Thank you. In line with your suggestion, we have added the corresponding reference at *Line 118*.
(Introduction)<br>• L58; give the reason for this	**R2-5:** Thank you for your suggestion. We have adjusted the overall structure of the introduction, and, in particular, we have added the following explanation in *Lines 62-65* to clarify the relationship between dietary demand and nitrogen emissions:<br><br>*"…Agricultural food systems remain the primary drivers of nitrogen cycle disruptions at national, regional, and global scales*[9]*. Therefore, ensuring the sustainable use of nitrogen necessitates measures that improve nitrogen use efficiency (NUE) while minimizing environmental impacts…"*
(Introduction)<br>• L58: Discuss the relevant studies available in the literature	**R2-6:** Thank you for your suggestion. As mentioned in **R2-5**, we have revised the overall structure of the introduction and, following your advice, we have further strengthened the discussion of existing studies on the relationship between dietary demand and nitrogen emissions. The revised text is as follows:

	Line 60-70: "To meet the nutritional needs of the global population without exceeding its safe planetary boundary, it is essential to consider the diversity of agricultural systems and the broader context of non-agricultural nitrogen releases. Agricultural food systems remain the primary drivers of nitrogen cycle disruptions at national, regional, and global scales[9]. Therefore, ensuring the sustainable use of nitrogen necessitates measures that improve nitrogen use efficiency (NUE) while minimizing environmental impacts. Despite origin-based solutions may offer more specific and impactful results, policymakers still have a wide range of options available to address nitrogen pollution across the whole food production and consumption chain[10, 11]. Integrated management across the whole chain and aligning policies across different environmental domains are critical to avoiding the risk of substituting one form of nitrogen pollution for another[12]…"
(Results and discussion)<br>• L78: Mention the period in the text	**R2-7:** Thank you for your suggestion. We now explicitly refer to this time period at *Line 100*. The revised text is as follows:<br><br>*"…Over the period 2000-2022, per capita nitrogen intake from plant-based foods rose by 16%, from 2.7 to 3.1 kg, whereas intake from animal-based foods increased by 40%, from 1.6 to 2.2 kg…"*
(Results and discussion)<br>• L82: When globalization of the food supply chain start?	**R2-8:** Thank you for your suggestion. As the starting point of the globalization of the food supply chain cannot be precisely defined, we have added contextual information at *Line 104* indicating that this process accelerated in the early 21st century. The revised text is as follows:<br><br>*"…Since the late 20th century and especially during the early 21st century, the globalization of food supply chains and urbanization have further enhanced access to animal products through improved distribution and affordability[22]."*
(Results and discussion)<br>• L89: Why it is only aquatic systems?	**R2-9:** Thank you for raising this point. We did not intend to focus solely on aquatic systems; this was an inappropriate wording choice. Our original intention was to refer to NO_3^- released into water bodies. To avoid potential misunderstanding, we have *removed the term "aquatic systems"* at *Line 112*, which makes the expression more accurate and fluent.
(Results and discussion)<br>• L95: No reference is directly related to agriculture and therefore, please use this reference to strengthen this claim: https://www.mdpi.com/2504-3129/4/4/28	**R2-10:** Thank you for your suggestion. We have replaced the original reference at *Line 118* with the one you recommended.
(Results and discussion)<br>• L98-113: The discussion on African countries is lacking.	**R2-11:** Thank you for your suggestion. We have added a more detailed discussion of Africa in this paragraph (*Lines 128-132*). The revised text is as follows:

Further, analysis based on the continent is also beneficial to give more information for readers.	*"…In 2022, per capita dietary nitrogen intake in Sub-Saharan Africa was 3.5 kg. Of this total, nitrogen from animal-based foods accounted for only 0.7 kg per capita, the lowest among all regions, indicating that food access and nutritional status in this region remain far below the global average…"*
(Results and discussion)<br>• L120: After rigorous restrictions on N emissions, these countries have decreased their emission. However, the emissions of these countries are still above most of the developing countries by many times. This statement has to be included with evidence (citations) to give a correct idea and avoid misunderstanding.	**R2-12:** Thank you for your suggestion. At *Line 147*, we have added a discussion noting that, despite substantial mitigation efforts, nitrogen emissions in high-income countries remain high, together with the relevant reference. The revised text is as follows:<br><br>*"…Yet, nitrogen releases in many high-income nations remain substantial due to persistently high resource demand and production intensity[23]…"*
(Results and discussion)<br>• L121: Include more citations to directly emphasise this claim. Suggestion is here: https://www.sciencedirect.com/science/article/pii/S0048969722080524	**R2-13:** Thank you for your suggestion. We carefully considered the reference you recommended; however, in light of the surrounding arguments and contextual focus, we chose to cite an alternative reference at *Line 153* instead. The added citation is as follows:<br><br>*"Liu, L., et al. Challenges for Global Sustainable Nitrogen Management in Agricultural Systems. J Agric Food Chem 68, 3354-3361 (2020). https://doi.org/10.1021/acs.jafc.0c00273"*
(Results and discussion)<br>• L123: Why are similar countries not included?	**R2-14:** Thank you for your comment. In this part of the manuscript, we use Brazil, a major agricultural producer, as a representative example to illustrate how agricultural intensification and the expansion of livestock production have contributed to increased nitrogen losses. The data presented for Brazil are intended to support the argument made in the preceding sentence, which is why we did not extend the discussion to additional countries at this point. We hope this clarification addresses your concern.
(Results and discussion)<br>• L135: Does this imply that plant-based N ensure food security than animal-based?	**R2-15:** Thank you for your comment. We have revised the discussion in *Lines 167-170*. The cited literature demonstrates that plant-based nitrogen sources contribute more effectively to food security than animal-based sources, and we now state this point more explicitly in the text. We have also further refined and expanded the discussion of plant-based nitrogen. The revised text is as follows:<br><br>*"…Compared to animal-based foods, dietary nitrogen from plant-based sources is generally associated with lower environmental burdens and is regarded as one of the most influential strategies for feeding a projected global population of 10 billion by 2050[24]. Diets rich in plant-based foods are also linked to reduced diet-related morbidity[25]."*
(Results and discussion)<br>• L135-138: Please elaborate on	**R2-16:** Thank you for your suggestion. We have revised the wording in this section accordingly. The changes are consistent with those

this with suitable reasons	described in **R2-15**.
(Results and discussion)<br>• L145: Not only this, the N input per kg of output is also very high. Please include this with references.	**R2-17:** Thank you for your suggestion. We have added further discussion in *Lines 176-178*, together with the relevant reference. The revised text is as follows:<br><br>*"…While livestock production contributes to economic and social development goals, it accounts for one-third of current anthropogenic nitrogen releases, 68% of which are associated with feed production, thereby posing a major sustainability challenge*[26]*…"*
(Results and discussion)<br>• L147: What kind of unsuitable practices? Discuss with evidence. However, I believe apart from these reasons, other factors are also contributing. Please search for other possible reasons and include them.	**R2-18:** Thank you for your suggestion. We have expanded the description in *Lines 178-181* to provide a more detailed discussion of inappropriate livestock production practices currently observed in China, and we have also added potential additional explanations. The revised text is as follows:<br><br>*"…In China, the correlation between animal-based nitrogen intake and environmental SDGs is negative (Supplementary Table 5), highlighting unsustainable practices in the livestock sector, such as low manure recycling rates and inadequate manure management*[27]*."*
(Results and discussion)<br>• L170: give a citation	**R2-19:** Thank you for your suggestion. We have added an additional reference to support this part of the discussion. The new citation is as follows:<br><br>*Line 211: "Loiseau, E., et al. Green economy and related concepts: An overview. J Clean Prod* ***139****, 361-371 (2016). 10.1016/j.jclepro.2016.08.024"*
(Results and discussion)<br>• L175 and 181: The authors recommend using meta-analysis to substantiate such claims, as warming and rainfall can exert both positive and negative effects on NUE and nitrogen losses. Therefore, instead of merely describing these effects, it is important to reinforce them with supporting evidence.	**R2-20:** Thank you for your suggestion. We have strengthened this part of the discussion by adding quantitative climate-related evidence at *Line 223 and Line 236*. The revised text is as follows:<br><br>*"…It also affects nitrogen intake, resulting in a reduction of -0.12, consistent with evidence that global warming alone could reduce crop yields by 21%*[28]*…"*<br><br>*"…Studies have found that heavy precipitation events would increase nitrogen loads in U.S. water bodies by about 20% by the end of the century*[29]*, with future hotspots of agricultural nitrogen loss expected in China, Southeast Asia, East Africa, and Brazil*[30]*…"*
(Results and discussion)<br>• L188: Please change the title to Factors Contributing to the SDGs	**R2-21:** Thank you for your suggestion. We have revised the section title, as recommended, to *"Factors contributing to the SDGs."*
(Results and discussion)<br>• L200: Under this section, please discuss which country or region is mainly influenced by all these three factors	**R2-22:** Thank you for your suggestion. All regions are influenced by the combined effect of the three factors, and the main argument of this section is to discuss the differences in their contributions. To enhance the completeness of the discussion, we have added the following at *Line 253*, as per your suggestion. The revised text is as follows:

	"...In developing countries such as Brazil and India, the combined impact of economic, natural, and nitrogen-related factors is greater and more pronounced compared to developed nations, due to their economic conditions and environmental resilience. Notably, the impact of socio-economic factors exceeds the global average in many regions..."
(Results and discussion)<br>• L221: This N reflects the N used for agriculture or N consumption?	**R2-23:** Thank you for your suggestion. We have added the following clarification at *Line 282*. The revised text is as follows:<br><br>*"...In low-income countries, nitrogen-related constraints dominate, contributing 46%, with both agricultural nitrogen use and nitrogen consumption playing crucial roles, as basic survival and nutrition concerns often take precedence over climate adaptation strategies[31]."*
(Results and discussion)<br>• L217: In Figure 4d, how is temperature a leading factor for high-income countries? Please discuss this in the relevant text.	**R2-24:** Thank you for your suggestion. We have added a discussion at *Line 273* to explain why temperature has become a major influencing factor in high-income countries. The revised text is as follows:<br><br>*"High-income and upper middle-income countries, having made significant strides toward the SDGs, are now experiencing more stable economic development. With their economies stabilized at a high level, and with the Zero Hunger goal achieved and nitrogen losses managed[32], the potential for further improvements in traditional socio-economic factors is relatively limited. However, due to its widespread, long-term, and cross-border impacts, global warming has gradually become a crucial factor limiting sustainable development in these countries, particularly in the face of climate risks, energy transition pressures, and international responsibilities[33]."*
(Results and discussion)<br>• L229: Natural resource exploitation is not only due to economic pressure, but the invasion of developed countries is also a main reason for this	**R2-25:** Thank you for your suggestion. We have added a discussion and citation at *Line 289*. The revised text is as follows:<br><br>*"...Conversely, lower-income countries may overexploit natural resources due to economic pressures and the invasion of developed nations[34]..."*
(Results and discussion)<br>• L243: Apart from these, strong policy and strict implementation of policies are essential for countries to have ample access to N fertilizers	**R2-26:** Thank you for your suggestion. We have enhanced the policy recommendations for low-income countries at *Line 308*. The revised text is as follows:<br><br>*"...Additionally, by gaining insights into farmers' needs and preferences, governments can implement systematic policy interventions to improve fertilizer accessibility[14]. This is a key strategy for enhancing agricultural productivity, increasing farmers' incomes, and ultimately reducing poverty levels..."*
(Results and discussion)<br>• L246: This reactive approach is not advisable. It is more sensible to focus on minimizing nitrogen pollution rather than strengthening public health	**R2-27:** Thank you for pointing out the issue. We have revised the discussion at *Line 311*, removing the description related to public health measures and focusing more on fertilizer issues that are particularly relevant to low-income countries. The revised text is as follows:<br><br>*"...International support is also vital for addressing infrastructure*

measures targeting it.	*gaps, while sustainable agricultural practices, such as the recycling of organic resources, can foster both environmental sustainability and poverty alleviation*[15]*. Although economic growth may present challenges, careful monitoring and regulation of fertilizer use are necessary to prevent unintended environmental damage."*
(Results and discussion)<br>• L247: This is already in practice in developing countries, but this suggestion should be given to the developed countries	**R2-28:** Thank you for your suggestion. We have moved the practical recommendations regarding plant-based dietary structures into the policy discussion for developed countries at *Line 331*. The revised text is as follows:<br><br>*"…This approach can encourage sustainable farming practices and dietary changes, such as reducing animal protein consumption*[19, 20]*…"*
(Results and discussion)<br>• L257: What are these N recycling systems?	**R2-29:** Thank you for raising this issue. After reviewing it, we concluded that the term "nitrogen cycle" was not used appropriately in this context. As a result, we have revised the discussion on nitrogen-related issues for high-income countries at *Line 334*. The revised text is as follows:<br><br>*"…Investments in advancing NUE technologies, organic farming, and ecological practices can further reduce nitrogen's environmental footprint*[20]*. However, it is crucial that policies also support agricultural productivity and farmers' incomes, ensuring a balance between environmental goals and the economic realities of agriculture."*
(Method)<br>• L291: reference for this model?	**R2-30:** Thank you for your suggestion. We have added the reference for the model at *Line 393*. The citation is as follows:<br><br>*"Gu, B. & Zhang, X. The Coupled Human And Natural Systems (CHANS) Nitrogen Cycling Model (2020). https://person.zju.edu.cn/bjgu#928507"*
(Method)<br>• L320: Give proper citation for the equations used in this study	**R2-31:** Thank you for your suggestion. We have added references for the formulas used in the statistical analysis section. The citations are as follows:<br><br>*Equations (2) and (3): "Building on the methodologies of previous studies*[35]*, the details of the PCA and PCR employed in this research are as follows…*<br><br>*Xu, J., et al. Soil health contributes to variations in crop production and nitrogen use efficiency. Nat Food* ***6****, 597-609 (2025). 10.1038/s43016-025-01155-6"*<br><br>*Equations (4) and (5): "Building on previous studies*[35, 36]*, the scores and contributions of nitrogen-related factors, for example, were calculated as follows…*<br><br>*Xu, J., et al. Soil health contributes to variations in crop production and nitrogen use efficiency. Nat Food* ***6****, 597-609 (2025). 10.1038/s43016-025-01155-6*

	*Abel, C., et al. The human–environment nexus and vegetation–rainfall sensitivity in tropical drylands. Nat Sustain **4**, 25-32 (2021). 10.1038/s41893-020-00597-z"*
(Supplementary document)<br>• What is the source for the supplementary Figure 2?	**R2-32:** Thank you for your comment. We have indicated the data sources in the caption of *Supplementary Figure 2*. The revised caption is as follows:<br><br>***"Supplementary Figure 2 | Historical trends of nitrogen intake and release.***<br>*(a) Total nitrogen intake from plant-based and animal-based food, as well as per capita nitrogen intake from 2000 to 2022. Nitrogen intake data was calculated based on the FAO Food Balances database (https://doi.org/10.4060/cd7162en), covering plant-based and animal-based food categories. An apparent step change around 2010 in dietary nitrogen intake may reflect updates to the FAO Food Balances series (e.g., a transition between database versions and/or revisions in balancing procedures, commodity coverage, and conversion factors), changes in country reporting and data completeness, and associated recalibration or imputation in the underlying food supply statistics. We therefore interpret this discontinuity as a potential data artifact rather than an abrupt real-world shift. (b) Total nitrogen release to air and to water, as well as per capita nitrogen release from 2000 to 2022. Nitrogen release data is based on the CHANS model outputs."*
(Supplementary document)<br>• Supplementary Figure 4: The Figure caption does not fully describe the legend of the figure.	**R2-33:** Thank you for pointing out this issue. We have enhanced the caption of *Supplementary Figure 4* to include complete explanations for all the legends. The revised caption is as follows:<br><br>***"Supplementary Figure 4 | Comparison of nitrogen release to air and water from the CHANS model with other models and literature.***<br>*This figure presents a comparison of global nitrogen emissions to air (NH_3 (a), NO_x (b), N_2O (c)) and nitrogen losses to water (NO_3^- (d)) as estimated by the CHANS model, alongside estimates from other models and relevant literature[43-64]. "CHANS" refers to the Coupled Human and Natural Systems model used in this study; "EDGAR_v8.1" refers to The Emissions Database for Global Atmospheric Research version 8.1; "CEDS_v2024" represents the Community Earth-atmosphere Data System version 2024; "GAINS_v4.0" refers to the Greenhouse Gas and Air Pollution Interactions and Synergies model version 4.0; "IMAGE-GNM" stands for the Integrated Model to Assess the Global Environment - Global Nutrient Model; "IASI_NH3" refers to the Infrared Atmospheric Sounding Interferometer retrieval of NH_3; "MASAGE_NH3" represents the Magnitude And Seasonality of Agricultural Emissions model for NH_3; "HTAP_v3.0" refers to the Hemispheric Transport of Air Pollution experiment version 3.0; "CAMS-GLOB-ANT_v6.1" represents the Global anthropogenic emissions for the Copernicus Atmosphere Monitoring Service*

	simulations version 6.1; "IIASA-EclipsE" refers to the Evaluating the Climate and Air Quality Impacts of Short-Lived Pollutants Project by the International Institute for Applied Systems Analysis; "FAOSTAT-IPCC" refers to the FAOSTAT Emissions Totals Database developed by the Food and Agriculture Organization (FAO), aligned with the Intergovernmental Panel on Climate Change (IPCC); "PRIMAP-hist" stands for the Potsdam Realtime Integrated Model for probabilistic Assessment of emissions Paths dataset; "PIK" refers to the Potsdam Institute for Climate Impact Research; "UNFCCC" represents the United Nations Framework Convention on Climate Change; "GLOBIOM" refers to the Global Biosphere Management Model."

Reviewer #3:

Reviewer comment	**Number**, Response and Change
(Key results)<br>The key message covers the overarching patterns to N trends and how these interact with SDG and overall social, economic, and environmental trends. Here, there are some synergies but often trade-offs between these goals, especially the social and economic goals with the environment. The trends vary according to country and regional contexts. It was interesting to see that there were positive and negative implications of plant-based and meat-based diet trends across different regions.	**R3-1:** Thank you for your recognition of the key findings of the study.
(Validity)<br>Noting at the outset that I am not fully familiar with these models or the details of the analysis. That said, most of the analysis appears valid. The only area I find somewhat uncertain is the section on nitrogen contribution quantification, particularly the conclusions in lines 175–186.<br><br>It may be worth unpacking further how nitrogen also affects climate change, as the patterns of influence are not straightforward. Certain nitrogen compounds can have either warming or cooling effects, which complicates interpretation. If Nr waste influences climate change, and this study also aims to assess how climate change affects nitrogen intake and release, then it would be helpful to provide a clearer explanation of these reciprocal relationships.	**R3-2:** Thank you for your recognition of the validity of most of the analysis in our study. We have carefully revised the section on the quantification of the contribution of climate factors (*Lines 225-228*), further elaborating on how nitrogen influences climate change and its bidirectional relationship, in order to reduce uncertainty. The revised text is as follows:<br><br>*"...Additionally, the bidirectional influences of reactive nitrogen on climate cannot be overlooked, with both warming effects form N_2O emission and cooling effects form NO_x and NH_3 emission through aerosol formation[37], and the warming influence of nitrogen emission significantly outweighs the regional cooling effect on a global scale[38]."*
(Significance)<br>This feels like a significant paper, and whilst some of the patterns and categories are fairly crude, that is often the case given the	**R3-3:** Thank you for acknowledging the academic significance and innovation of our study.

data available at this scale. There are still important trends and drawing on the SDG links to N and quantifying is important to evidence how N has implications for the all the SDGs in both positive and negative ways. This study provides a unique approach and findings in that regard.	
(Data and methodology)<br>The data, from what I understand, feels robust. However, it feels the paper could be more explicit about some of the caveats of the different data sets available beyond what is already there. Such as the variations in quality and data sources across certain countries and regions.	**R3-4:** Thank you for your suggestion. We have addressed the uncertainty regarding data sources in the uncertainty discussion section of the Supplementary Methods. Following your recommendation, we have further emphasized the discussion on source uncertainty in *"Supplementary Method 4 | Uncertainty analysis"* of the SI. The revised text is as follows:<br><br>*"...However, due to differences in reporting practices, data collection methods, and reliance on estimations between countries and regions, especially in countries with limited or inconsistent data, the quality and sources of the data still present variability and uncertainty..."*
(Data and methodology)<br>Also, this may be the journal style but for the Figures 1 and others their source data was not explicitly stated in the figures. Then also is not clear what the base map was applied without endorsement using data from the GADM (https://gadm.org/)was applied? Was this to access data or create the map with admin boundaries? When you follow the link, it feels like it takes you to a slightly spurious webpage. So, suggest there is more explanation there of what GADM was used for and with further explanation of what datasets were applied in each figure.	**R3-5:** Thank you for your suggestion. We have added the original data sources in the captions of *Figure 1, Supplementary Figure 1, and Supplementary Figure 2*. For all other figures, the data is derived from this study. Additionally, we have clarified the use of GADM data in all relevant figures and updated the associated links. The revised captions are as follows:<br><br>***"Fig. 1. Global patterns and changes in dietary nitrogen intake from 2000 to 2022.***<br><br>*...The nitrogen intake data was calculated based on the FAO Food Balances database (https://doi.org/10.4060/cd7162en). The base map with administrative boundaries was created using data from the GADM (https://gadm.org/data.html), without endorsement."*<br><br>***"Supplementary Figure 1 | Trends of SDG index and performance in "Economy," "Society," and "Environment" from 2000 to 2022.***<br><br>*...The global SDG index data is sourced from the United Nations 2025 Sustainable Development Report (https://dashboards.sdgindex.org/). The base map with administrative boundaries was created using data from the GADM (https://gadm.org/data.html), without endorsement."*<br><br>***"Supplementary Figure 2 | Historical trends of nitrogen intake and release.***<br><br>*...Nitrogen intake data was calculated based on the FAO Food Balances database (https://doi.org/10.4060/cd7162en), covering plant-based and animal-based food categories...*

	...Nitrogen release data is based on the CHANS model outputs."
(Data and methodology)<br>Also, would it be a requirement to for to share the raw database? Current tables include the figures and summary tables but not the datasets used to make the calculations, recognizing that the data comes from secondary sources.	**R3-6:** Thank you for your suggestion. We have added a data sharing link in the "Data availability" section of the manuscript, providing access to the raw data on nitrogen intakes and releases. The revised text is as follows:<br><br>*Data availability: "Data supporting the findings of this study are available in its supplementary information files and archived in Zenodo (https://doi.org/10.5281/zenodo.17983565)."*
(Data and methodology)<br>Likewise, the fact that per capita was only used to standardize across the countries could be worth acknowledging that there would be different patterns observed if you were to look at crop land per ha, or absolute Nr values.	**R3-7:** Thank you for your suggestion. We have added a discussion in *"Supplementary Method 4 | Uncertainty analysis"* regarding the choice of using per capita standardization rather than per hectare crop land nitrogen releases or absolute Nr values. The primary reason for this choice is that our nitrogen losses encompass a broader scope, including industrial and residential emissions, rather than just agricultural emissions. Using per capita as a standardization method enhances comparability between countries. The revised text is as follows:<br><br>*"Aside from uncertainties related to data sources and models, this study uses per capita nitrogen intake and per capita nitrogen release to standardize the total nitrogen intakes and releases of each country, allowing for better comparability and analytical relevance. This approach has certain limitations, such as not using per hectare crop land nitrogen releases or comparing the absolute total nitrogen intakes and emissions. However, since the nitrogen releases in this study are based on a broader subsystem, including not only agricultural or forest/grassland releases but also nitrogen fluxes from industrial production and urban/rural residential sources, we chose the per capita standardization method to improve comparability between countries and regions."*
(Analytical approach)<br>The analytical approach appears to be strong with a number of comprehensive analyses applied. However, the overall approach is outside the scope of my expertise.	**R3-8:** Thank you for your recognition of the research methods and analytical approaches used in the study.
(Suggested improvements)<br>All the figures include the figures in the supplementary data were fairly difficult to read and too small, so recommend enlarging these. Also see above for other suggestions	**R3-9:** Thank you for your suggestion. We have increased the font size in all the figures in the manuscript and SI to enhance readability. The modified figures are shown below:<br><br>*"Fig. 1:*

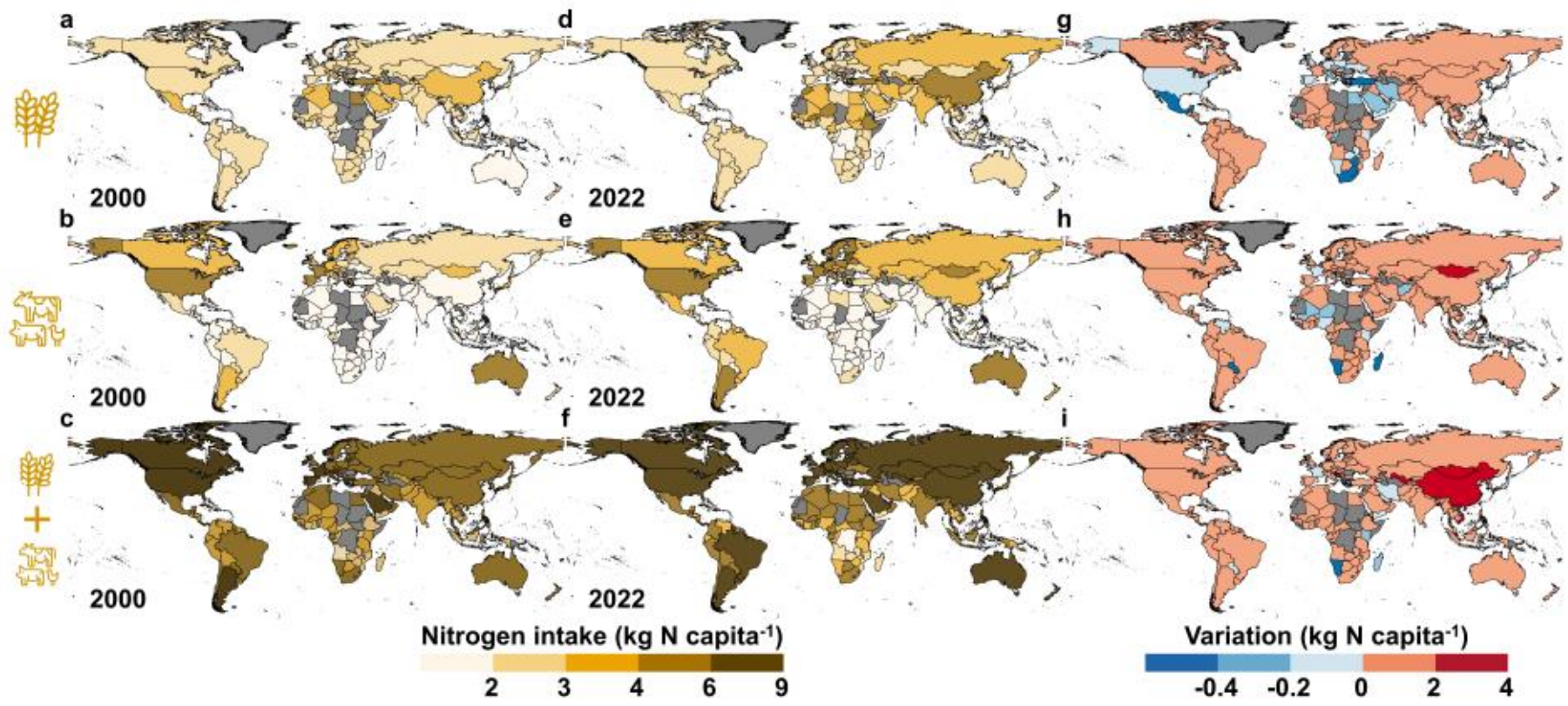

Fig. 2:

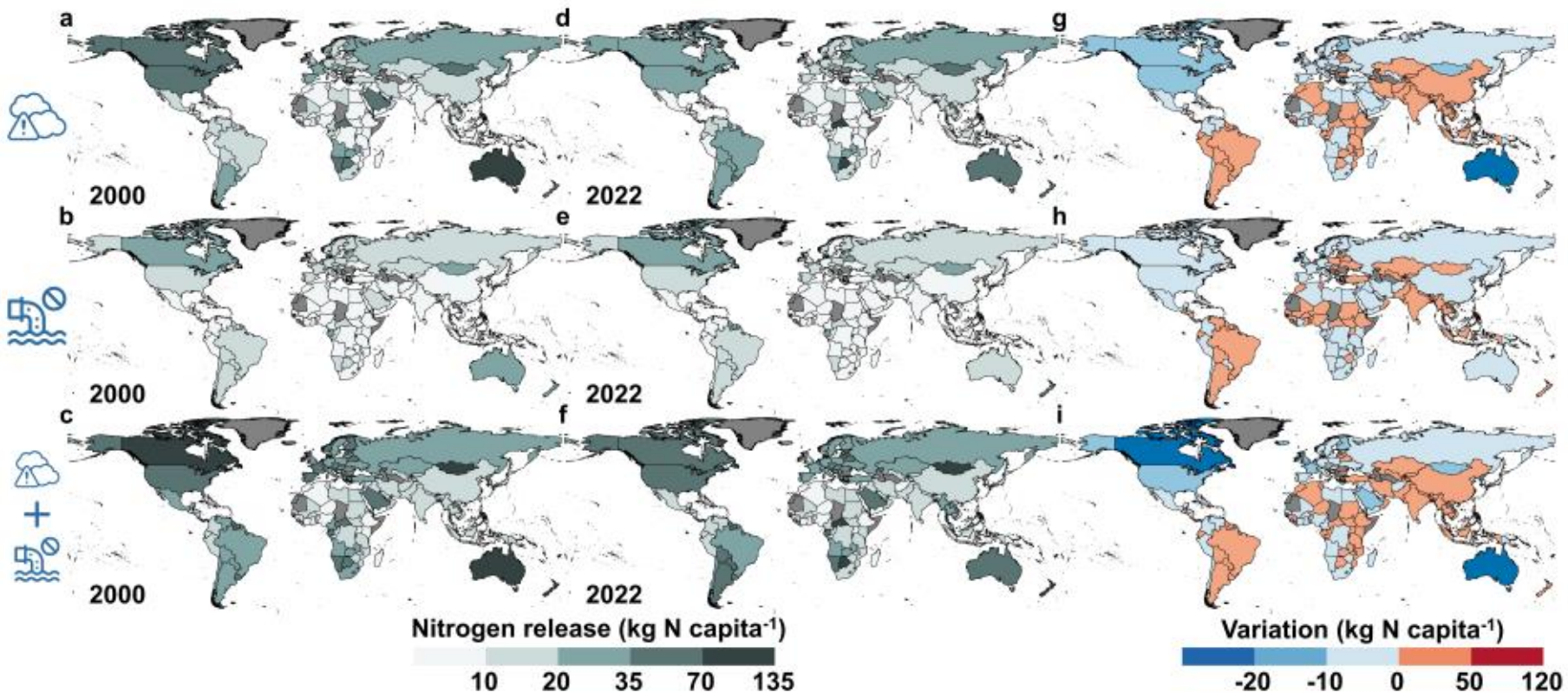

Fig. 3:

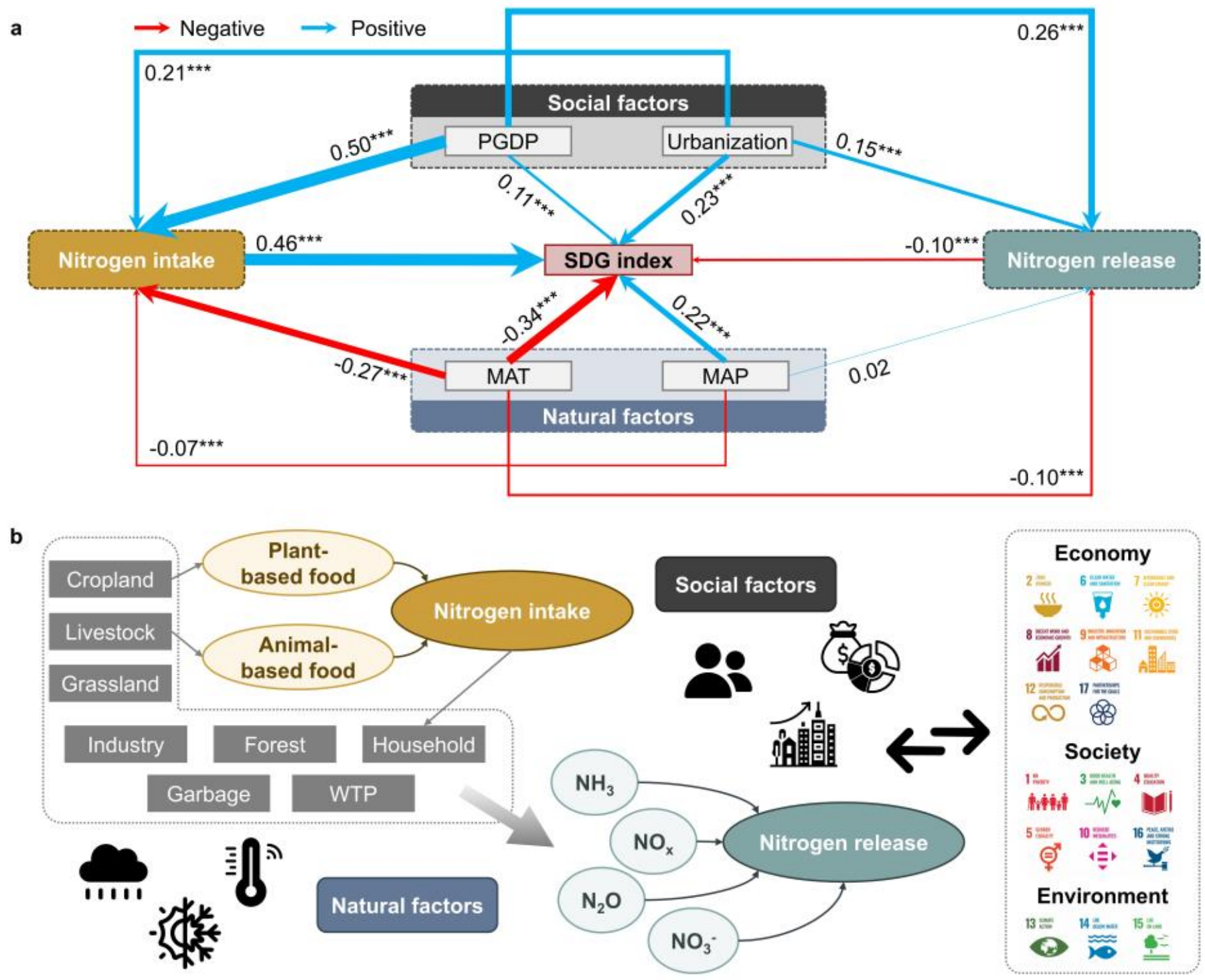

Fig. 4:

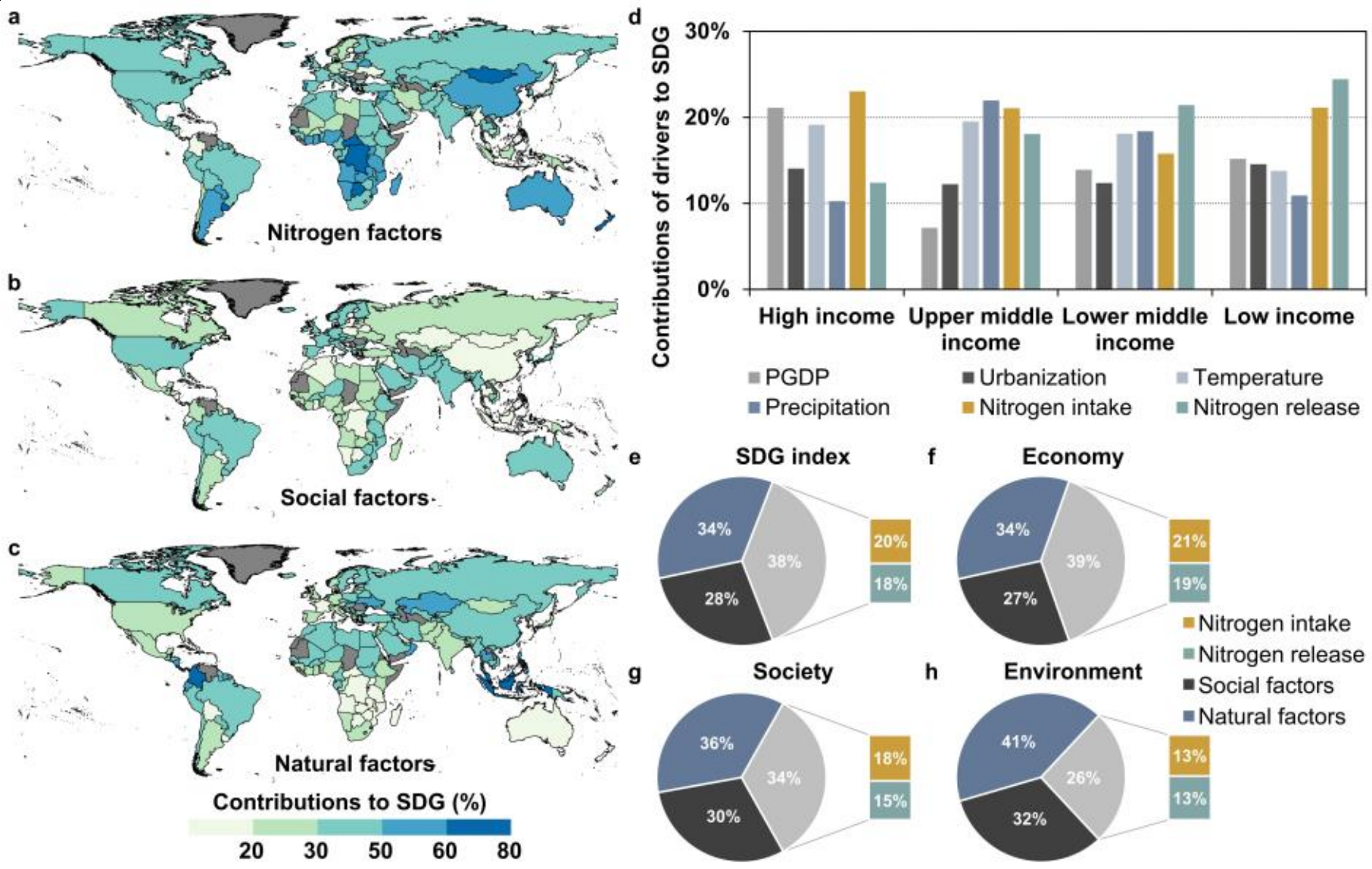

Supplementary Figure 1:

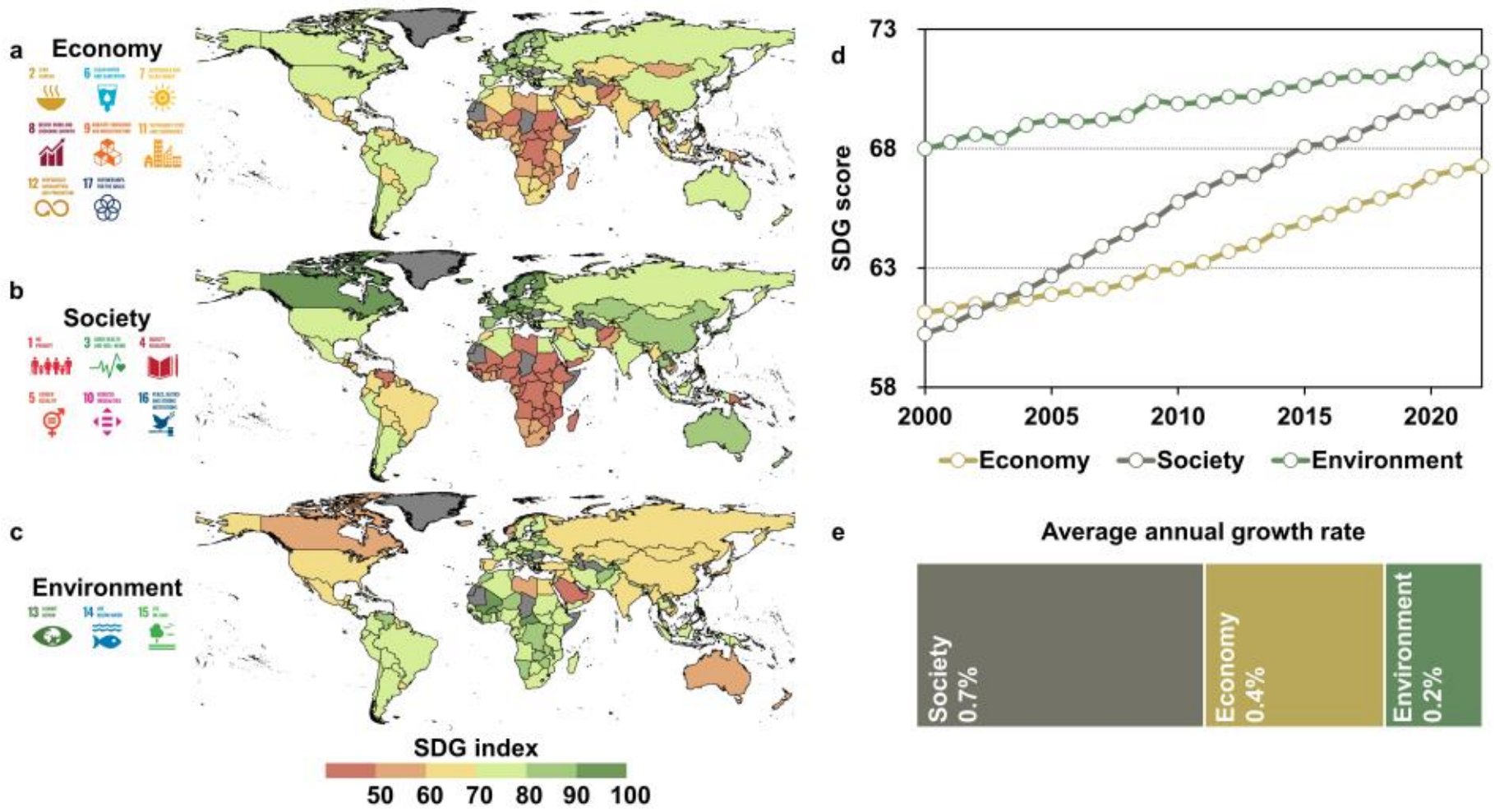

Supplementary Figure 2:

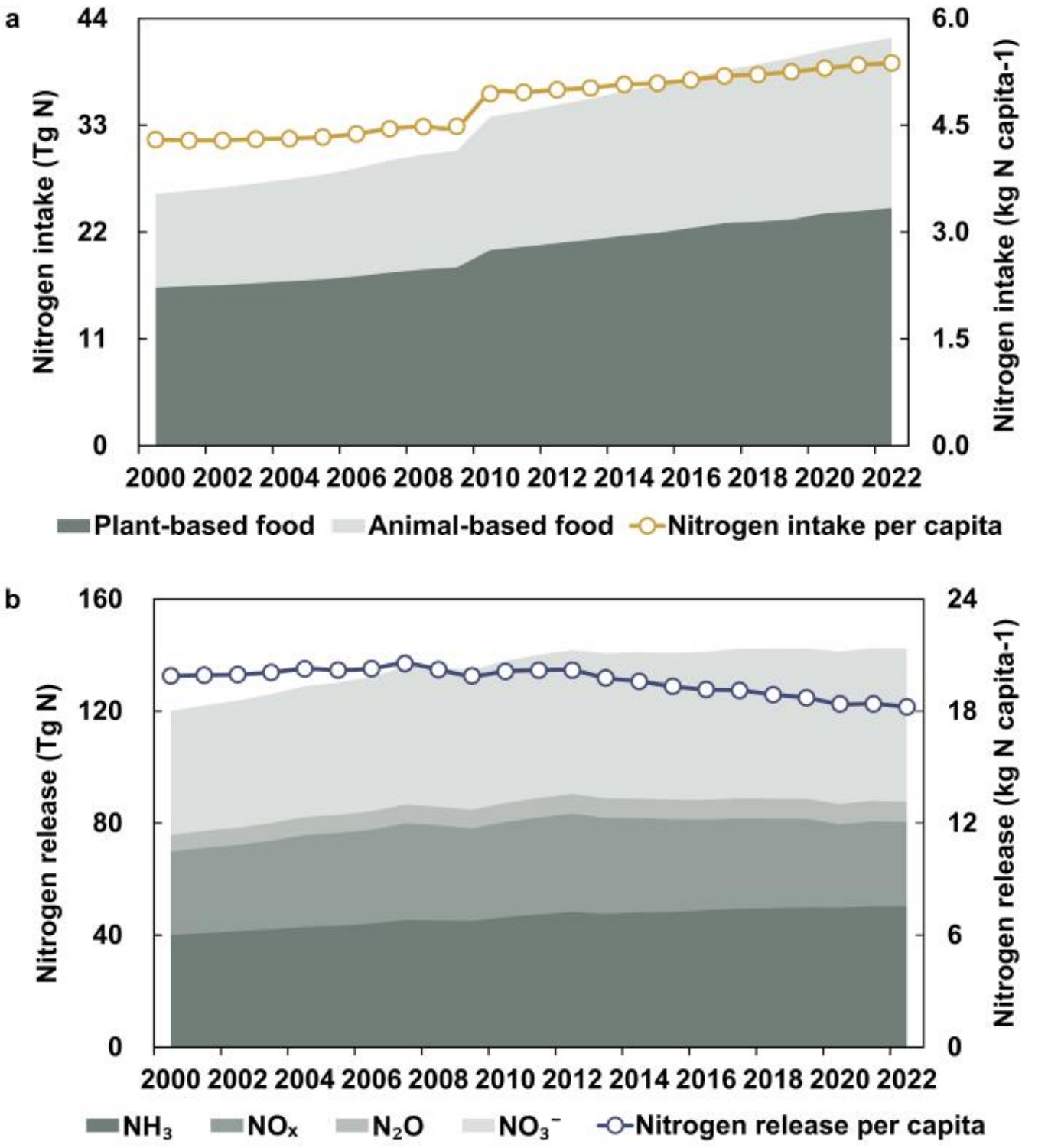

Supplementary Figure 3:

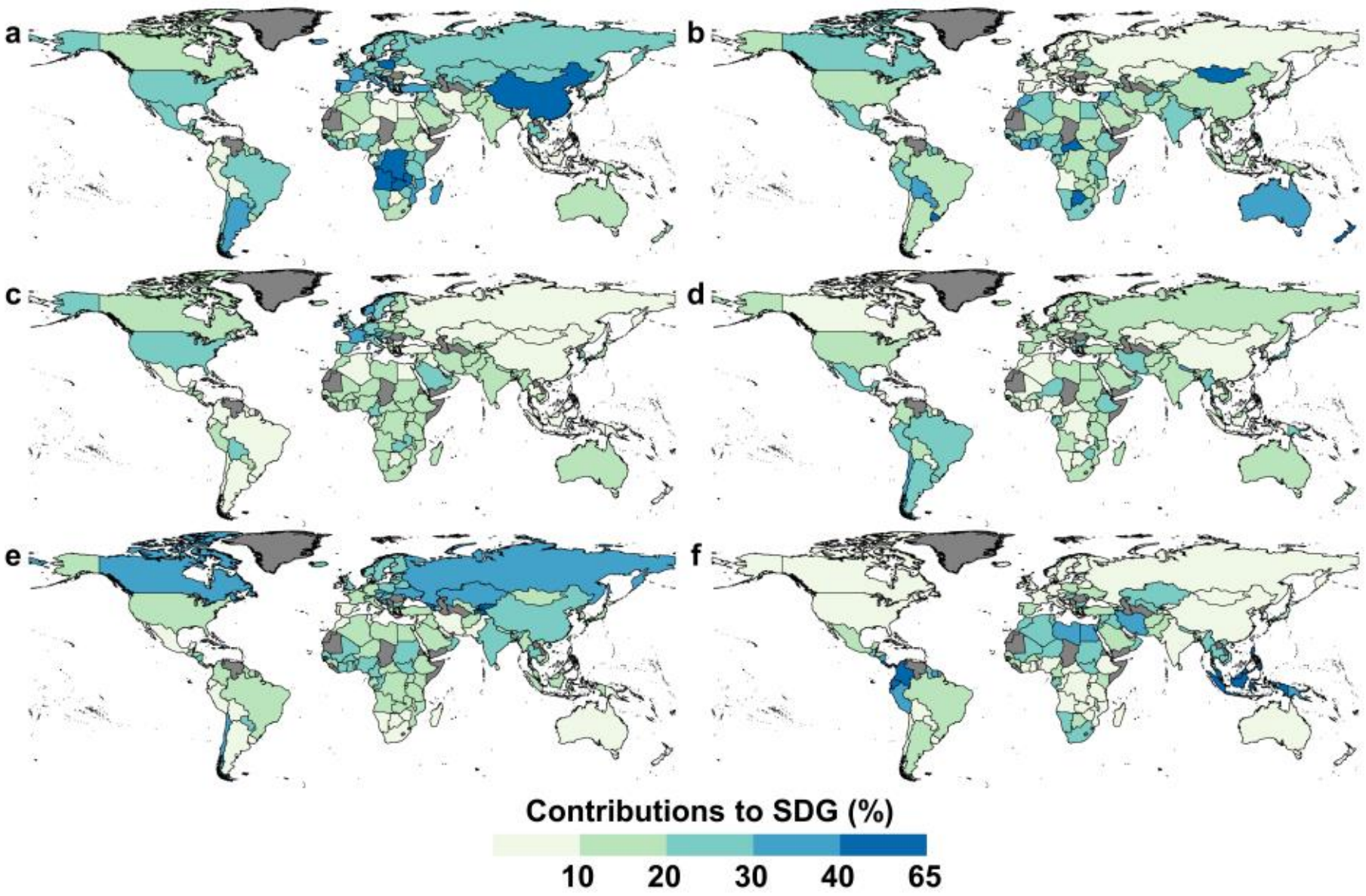

Supplementary Figure 4:

	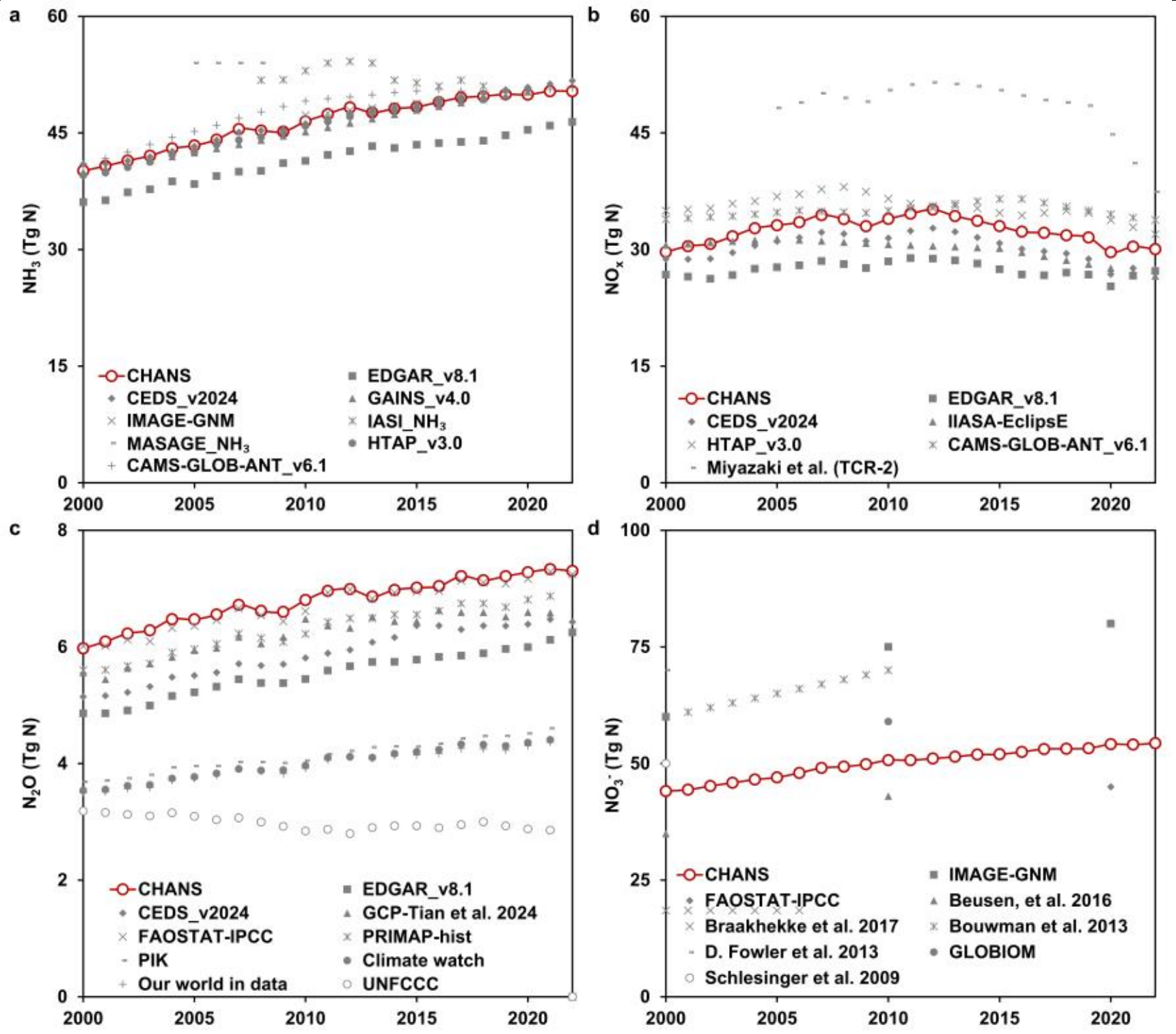 ”
(Clarity and context)<br>Recommend that intake and release are explained more clearly in the introduction. Also, the SDG index is not really well explained, nor what this data includes, so recommend addressing this in the text.	**R3-10:** Thank you for your suggestion. We have clarified the definitions of "nitrogen intake" and "nitrogen release" in the *Introduction*. Additionally, as per your advice, we have included an explanation of the "SDG index" and its data components in the introduction. The revised text is as follows:<br><br>*"…(1) examines temporal trends in per capita nitrogen intake from food systems, including both plant based and animal based consumption, and total nitrogen releases, including emissions to air and losses to water, (2) quantifies the effects of nitrogen related drivers on the SDG index, a composite indicator of national performance across the 17 SDGs calculated as the average of individual SDG scores…"*
(References)<br>The literature appear to have been referenced appropriately.	**R3-11:** Thank you for your recognition of the appropriate citation of references in the manuscript.

Reviewer #4:

Reviewer comment	**Number**, Response and Change
(General comments)<br>The paper addresses an important question, that of the dual role of nitrogen availability in agriculture as a key resource for food production and a source of environmental degradation through environmental loss. The paper states that few studies have addressed this issue as a cross-cutting theme of SDG. However, there is a vast literature on nitrogen use in agriculture that simultaneously assesses nitrogen-related food security and environmental impact. It is unclear what the new insights of the paper are on this theme. To this regard, a substantial discussion of the results obtained with the literature is missing.	**R4-1:** Thank you for highlighting the gap in our discussion regarding new insights in this study. We acknowledge that existing literature often evaluates the relationship between agricultural nitrogen use and its impact on food security and the environment, but these studies typically focus on the current nitrogen budget and progress on one or a few SDGs, such as SDG 2 and SDG 12. This study, however, takes a historical approach, analyzing nitrogen intake from human consumption and total system-wide nitrogen release trends, and their correlations with the progress of the comprehensive SDG index covering all 17 SDGs. The insight lies primarily in the broader system-wide nitrogen emission accounting and the inclusion of a more comprehensive assessment of SDG progress. We have revised and expanded the discussion on the content and novelty of the paper in the *Introduction*, and also highlighted the differences from existing literature. We hope this clarifies the new insights of the study. The revised text is as follows:<br><br>*"…Few studies have systematically quantified dietary nitrogen intake and nitrogen losses across the full supply chain across all sectors, or assessed how these metrics relate to progress on the overall SDG index. Their contributions to global sustainability therefore remain poorly resolved.*<br><br>*This study addresses this knowledge gap by integrating a global Coupled Human And Natural Systems (CHANS) model with structural equation modeling (SEM) and principal component analysis (PCA). We assess the influence of nitrogen on SDG progress across 166 countries from 2000 to 2022. Specifically, the study (1) examines temporal trends in per capita nitrogen intake from food systems, including both plant based and animal based consumption, and total nitrogen releases, including emissions to air and losses to water, (2) quantifies the effects of nitrogen related drivers on the SDG index, a composite indicator of national performance across the 17 SDGs calculated as the average of individual SDG scores, (3) explores interactions among nitrogen dynamics, socioeconomic factors, and environmental conditions, and (4) evaluates the relative contributions of these drivers to SDG performance. By embedding nitrogen flows within broader socioeconomic and climatic contexts, this study provides a comprehensive assessment of nitrogen's role in shaping global sustainability. The findings offer actionable insights for improving nitrogen management and for supporting integrated policies aligned with the 2030 sustainable development agenda."*
Moreover, the system boundary considered in the paper is also unclear as well as the internal system flows that are addressed. In addition, how the different models used are integrated in a	**R4-2:** We greatly appreciated this comment, which points out a crucial gap in our methodology description. In the revised manuscript, we have redrafted the *"Methods"* section and *"Supplementary Method 1"* to clearly define the system boundaries, map the internal nitrogen flows, and illustrate the integrated modelling framework (*Figure R1*). The revised text is as follows:

consistent modelling framework? Although some description is provided on the CHANS balance model, the other models used are not described, nor the integration between them.	***Methods-National-scale nitrogen budget:***<br><br>*"The national-scale nitrogen release data used in this study were derived from the global nitrogen budget constructed by the CHANS model[39]. CHANS is a data-driven mass-balance model that tracks nitrogen inputs, outputs, and accumulation across 14 coupled subsystems: cropland, livestock, grassland, human, industry, aquaculture, forest, pets, urban lawns, solid waste, wastewater, atmosphere, surface water, and groundwater (Supplementary Figure 5). It resolves multiple reactive nitrogen fluxes, including atmospheric emissions of N_2O, NO_x, and NH_3, and aquatic losses of NO_3^-, among others. Following Gu et al.[39, 40], we extracted NH_3, NO_x, N_2O, and NO_3^- fluxes from relevant subsystems. Additional methodological details, including the sector-specific nitrogen budget calculations and a full description of the model framework, are provided in the Supplementary Methods.*<br><br>*The spatial boundary of CHANS covers 166 countries (national scale) in this study, and results are reported as both national totals and per-capita values over 2000-2022 for all core analyses. While the model internally represents 14 subsystems, results are aggregated into eight policy-relevant emitting sectors: cropland, livestock, grassland, forest, household, industry, garbage (solid-waste) treatment, and wastewater treatment plants (WTP). External inventories with shorter temporal coverage were used for calibration/validation only. Nitrogen releases from natural ecosystems (e.g., wetlands, oceans, wildlife) are not included in national losses. For the SDG regressions, we use NO_3^- as the water-quality pressure indicator and do not include organic nitrogen exports to water. Finally, to avoid double counting, dietary nitrogen intake reflects apparent consumption (including net trade), whereas nitrogen releases are reported on a territorial (production-based) basis."*<br><br>***Supplementary Method 1 | System boundary and internal nitrogen cascade in the CHANS framework:***<br><br>*"The Coupled Human and Natural Systems (CHANS) model provides a robust framework for examining nitrogen (N) dynamics across interconnected human and natural environments. In this study, we used the global extension of CHANS (CHANS) to quantify nitrogen cycling within a harmonized system boundary for 166 countries over 2000-2022. The framework applies a process-driven, mass-balance approach to track nitrogen inputs, internal transfers, transformations, and releases across eight policy-relevant subsystems: cropland, livestock, industry, household, wastewater treatment plants (WTP), garbage (solid waste), forest, and grassland. The model integrates multiple data streams, including global statistical datasets, experimental and meta-analytical evidence, pollutant inventories, and customized emission factors (EFs), to represent key nitrogen pathways and associated environmental pressures.*

	Within this boundary, internal nitrogen dynamics are explicitly represented as a cascade of transfers and transformations linking production, consumption, treatment, and life-support compartments. Nitrogen enters the system through inputs such as biological fixation and synthetic fertilizer, circulates through production subsystems (cropland, livestock, and industry), and is transferred to the consumption subsystem (households) via food and goods chains. Nitrogen subsequently moves through treatment subsystems (WTP and garbage), before being released to life-support systems (atmosphere and hydrosphere).<br><br>*The model accounts for major reactive nitrogen flows, including (i) volatilization and deposition, whereby NHx and NOy emitted from agricultural activities, waste handling, and selected industrial uses are deposited back onto receiving land subsystems (cropland, grassland, and forest) using established country-level deposition datasets (Supplementary Method 3); (ii) soil nitrification–denitrification processes in cropland and grassland that transform reactive N into* N_2O*,* N_2*, and* NO_3^-*, with gaseous emissions and hydrological losses quantified and balances closed using NUE and N surplus; (iii) a hydrological cascade in which* NO_3^- *leaching and runoff from soils and manure-affected land enter surface- and groundwater pathways, while domestic and industrial wastewater N is routed through WTPs where nitrification/denitrification generate* N_2O/N_2 *and residual N is discharged to water or transferred via sludge to solid-waste systems; and (iv) recycling loops (e.g., straw return, manure application, and human excreta reuse) that return reactive N to croplands, alongside transfers of harvested crop N to livestock and households (dietary intake), with excretion partitioned between fields and WTPs depending on sanitation coverage. Terminal sinks and storage are also represented, with* N_2 *treated as an endpoint and biomass/soil accumulation tracked as temporary storage, particularly in forests.*<br><br>*To ensure internal consistency, we integrated multi-source datasets (e.g., FAOSTAT, the World Bank, region-specific emission factors) to inform the CHANS framework. Model outputs (nitrogen fluxes) were standardized and temporally aligned with the SDG dataset from the Sustainable Development Report. The resulting integrated dataset was assembled into a country-year matrix, which served as the core input for the SEM and PCA analyses described in the Methods."*

	Third Party Material Removed<br><br>***Figure R1 | Overview of the CHANS framework and the integration process. (for reviewer response only)***<br><br>*This figure illustrates the integration of various datasets and models within the CHANS framework for assessing global nitrogen cycling. It includes the data collection process, literature review, and datasets used (e.g., FAOSTAT, IPCC, EDGAR, and others). The model structure incorporates 14 subsystems and 7 sectors, covering nitrogen inputs, outputs, accumulation, and deposition across global regions. The final nitrogen budget is analyzed with spatial-temporal patterns, regional variations, and source contributions, feeding into uncertainty analysis and model optimization. This approach allows for a comprehensive evaluation of nitrogen dynamics from 1980 to 2022.*
The paper does not state neither the research hypothesis and assumptions on model parameters. More generally, the way the method and results are presented makes understanding the study very difficult. Authors should explain more thoroughly the methods: be more explicit about which data are used for which part of the model / analysis; be more explicit about what type of results are expected at each step of the method; in order for the reader to more easily identify how the results were obtained. The evidence used to calibrate the model and validate the modelling results is unclear. Furthermore, the paper employs path coefficients—a statistical measure used in structural equation modelling to quantify the strength and direction of relationships	**R4-3:** Thank you for your valuable suggestion. We have carefully revised the Methods section to clarify the data usage and the expected objectives for each model. Regarding your comments on the SEM approach, we have also revised this part and provided a more precise definition and application of path coefficients. The revised Methods section is as follows:<br><br>*"<u>Multiple regression analysis.</u> To assess the impact of nitrogen-related factors on the SDG index, we used multiple regression analysis. The four parameters considered are: per capita plant-based nitrogen intake, per capita animal-based nitrogen intake, per capita nitrogen release to air, and per capita nitrogen release to water. These variables were chosen based on their direct and indirect relationships to both environmental and socio-economic SDGs, and were analyzed using multiple regression with STATA/MP 18.0 software. The regression model was formulated as follows:*<br><br>$SDG\ index = \alpha \cdot N_{plant} + \beta \cdot N_{animal} + \gamma \cdot N_{air} + \delta \cdot N_{water} + \varepsilon + \theta_1 + \theta_2$ *(1)*<br><br>*where α, β, γ, and δ represent the coefficients calculated through regression analysis; ε is the constant term; θ_1, θ_2 are the error terms. N_{plant} represents the nitrogen content in per capita plant-based food intake, N_{animal} represents the nitrogen content in per capita animal-based food intake, N_{air} represents the nitrogen content in per*

between variables. However, as far as I understand, it does not precisely define and justify their application in this context.	*capita atmospheric nitrogen emissions, and* N_{water} *represents the nitrogen content in per capita nitrogen loss to water.*<br><br>*The model uses panel data from 2000 to 2022, which includes nitrogen intake, nitrogen release, and the SDG index for 166 countries. All nitrogen-related factors are expressed in kg of nitrogen per capita per year. To control for time-invariant national factors (e.g., long-term political policies, governance measures) that could influence nitrogen levels, we included a national-level intercept term (*θ_1*). Additionally, to account for global temporal trends (e.g., economic crises, political events), we included a year intercept term (*θ_2*). The objective of this regression analysis is to evaluate the influence of nitrogen-related variables on SDG progress, specifically to quantify the strength and direction of their relationships with the SDG index. This analysis provides insight into how different forms of nitrogen consumption and release contribute to the advancement of the SDGs.*<br><br>*Structural equation modeling. SEM was used to explore the complex relationships between nitrogen-related factors, socio-economic factors, environmental conditions, and the SDG index. SEM is an effective statistical tool for analyzing the interdependencies between multiple variables, including both observed (explicit) and latent (unobserved) variables. It provides a comprehensive understanding of both direct and indirect effects among factors, offering a more nuanced and holistic view of the relationships involved. Standardized path coefficients are used to represent the values derived from the path analysis in SEM*[35, 41]*. These coefficients indicate the strength and direction of relationships between variables in the model. They are standardized, meaning they are scaled within a range from -1 to 1, making it possible to compare different variables regardless of their original units. Path coefficients close to 1 or -1 indicate a strong relationship, while coefficients near 0 suggest a weak or no direct relationship. These coefficients help clearly identify the magnitude and direction of each relationship in the model, enabling comparisons of different paths within the model and providing insight into the overall impact of one variable on another. This makes them a key tool for assessing the contributions of various independent variables to the dependent variable and for understanding the structure of the correlation network.*<br><br>*While SEM can be used to test causal relationships, it is important to clarify that this study focuses on correlations rather than directly establishing causal pathways between variables. The goal of using SEM in this study was to model the multivariate relationships between nitrogen-related factors, social and environmental factors, and their collective impact on SDG progress. We aimed to understand both the direct and indirect associations of nitrogen on the SDGs, as well as the influence of socio-economic and environmental factors on nitrogen-related variables, to ultimately present their direct, indirect, and net effects, with a particular focus on how socio-economic and environmental factors mediate these relationships.*<br><br>*First, we identified potential interactions among the key variables based on the theoretical framework of nitrogen's role in driving SDG*

	outcomes, incorporating prior empirical knowledge on nitrogen's impact on agricultural productivity, food security, and environmental sustainability. After selecting the key variables, we used statistical tests to evaluate the significance of each variable's contribution to the model. Variables with insignificant contributions were progressively removed, and we compared the full model with the simplified version using goodness-of-fit statistics.<br><br>*To assess the model fit, we employed three widely accepted indices: SRMR (Standardized Root Mean Square Residual), with a value less than 0.08 indicating a good fit; RMSEA (Root Mean Square Error of Approximation), with a value less than 0.1 indicating a good fit; and the Chi-square test, where a P-value greater than 0.05 indicates a good fit. We selected the optimal structural equation model based on the Comparative Fit Index (CFI), where a higher CFI value indicates a better fit between the observed data and the theoretical model. The data inputs for SEM included the SDG index, nitrogen-related factors, socio-economic variables (such as GDP and urbanization rates), and environmental factors (such as temperature and precipitation), all of which were standardized and temporally aligned to ensure consistency across the model. Finally, Fig. 3, Supplementary Tables 6 and 7 present the direct and indirect impacts of all factors in the model, along with the standardized path coefficients, providing a detailed explanation of the roles and interrelationships of the variables within the model.*<br><br>*The multiple regression and SEM analyses in this study provide a comprehensive approach to understanding the relationships between nitrogen-related factors and SDG progress. The methods used allow for the evaluation of both direct and indirect impacts, offering valuable insights into the roles of different factors to the SDGs."*
In addition, the way the nitrogen cascade (the sequential transformation of different reactive nitrogen compounds) is taken into account (if it is) in the modelling framework is not explained neither. The paper does not at all mention the nitrogen cascade although it targets linking specific reactive nitrogen compounds into a negative impact SDG framework.	**R4-4:** Thank you for raising this crucial point. In the revision, we have explicitly introduced the nitrogen cascade concept and have clarified how sequential transformations and cross-media transfers of reactive nitrogen (Nr) have been represented in our modelling framework. We have also added text and a schematic (*Figure R2*) to make these linkages transparent. The most relevant modifications to *Supplementary Method 1* are as follows:<br><br>*"The model accounts for major reactive nitrogen flows, including (i) volatilization and deposition, whereby NHx and NOy emitted from agricultural activities, waste handling, and selected industrial uses are deposited back onto receiving land subsystems (cropland, grassland, and forest) using established country-level deposition datasets (Supplementary Method 3); (ii) soil nitrification–denitrification processes in cropland and grassland that transform reactive N into N_2O, N_2, and NO_3^-, with gaseous emissions and hydrological losses quantified and balances closed using NUE and N surplus; (iii) a hydrological cascade in which NO_3^- leaching and runoff from soils and manure-affected land enter surface- and groundwater pathways, while domestic and industrial wastewater N is routed through WTPs where*

<table>
<tr>
<td></td>
<td>nitrification/denitrification generate N_2O/N_2 and residual N is discharged to water or transferred via sludge to solid-waste systems; and (iv) recycling loops (e.g., straw return, manure application, and human excreta reuse) that return reactive N to croplands, alongside transfers of harvested crop N to livestock and households (dietary intake), with excretion partitioned between fields and WTPs depending on sanitation coverage. Terminal sinks and storage are also represented, with N_2 treated as an endpoint and biomass/soil accumulation tracked as temporary storage, particularly in forests.”

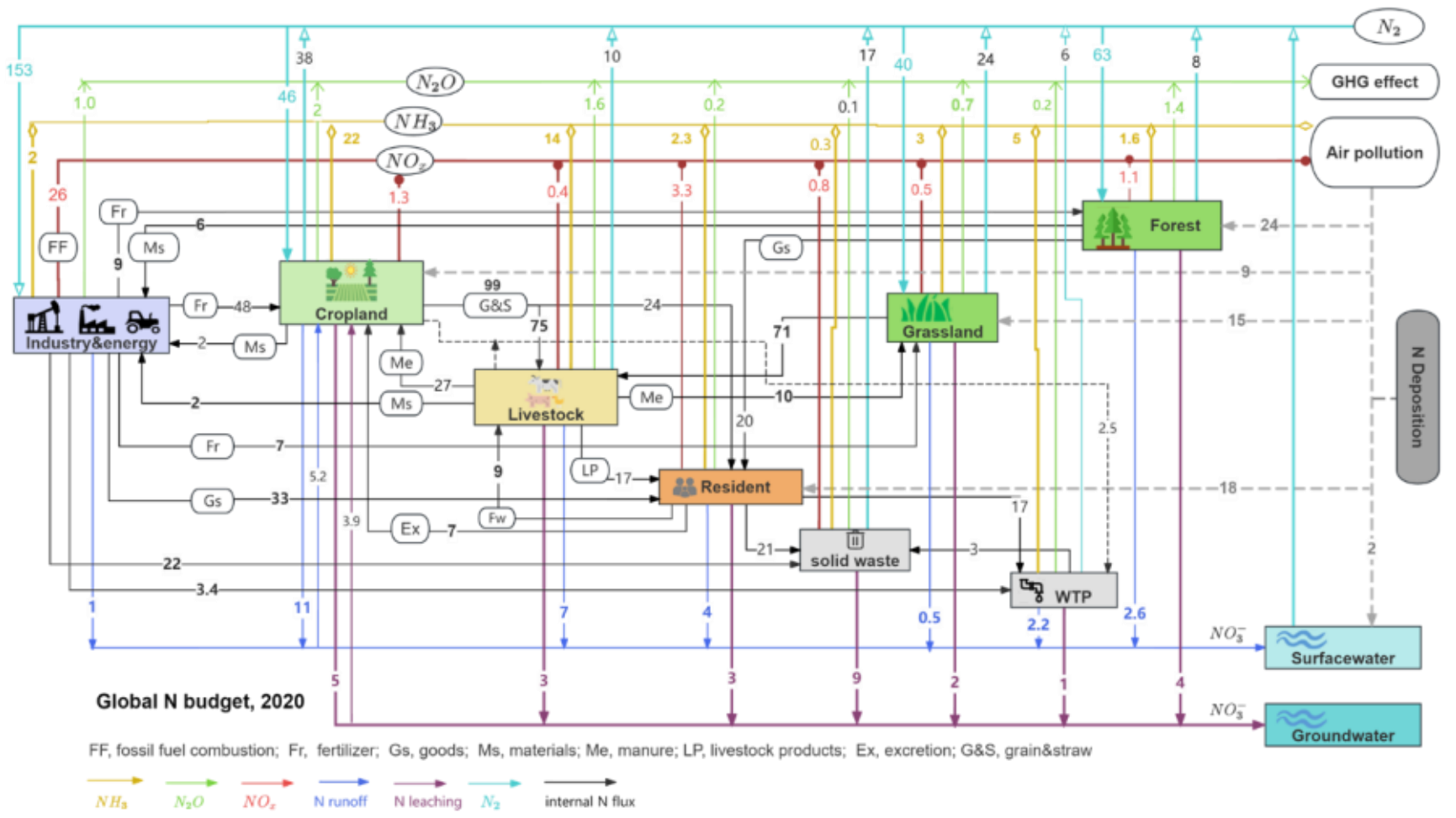

Figure R2 | Nitrogen cascade across subsystems in the CHANS model. (for reviewer response only)

This diagram illustrates the nitrogen cascade across various subsystems in the CHANS model, highlighting the transformations and cross-media transfers of Nr from input to final release. The arrows represent nitrogen fluxes between different compartments.</td>
</tr>
<tr>
<td>Also, the paper should be clearer in the results. Are the relations between the exposed variables correlations or actual causalities. Does the method allow to demonstrate causality and how?</td>
<td>R4-5: Thank you for your suggestion. This study uses a combination of SEM, PCA, and PCR to examine the driving relationships and contributions of nitrogen intake and nitrogen emissions to the SDGs. While SEM can hypothesize and test causal relationships, it cannot directly prove causality. PCA, on the other hand, reveals the underlying structure and correlation patterns among variables. Therefore, this study does not discuss actual causal relationships. To avoid confusion, we have carefully reviewed the relevant descriptions in the results section and revised all discussions of causal relationships to reflect correlation. For example, in Line 173, “a stronger effect” was replaced with “a stronger correlation”, Line 196, “exerts a strong positive direct effect on” was replaced with “shows a significant positive correlation with”, to enhance the clarity of the results section.

Additionally, we have added a clarification in the Methods section (Lines 460-461) to emphasize that this study focuses on the correlational relationships between the factors, rather than attempting to establish direct causal links. The revised text is as follows:

“While SEM can be used to test causal relationships, it is important to</td>
</tr>
</table>

	clarify that this study focuses on correlations rather than directly establishing causal pathways between variables...”
Another concern arises from the paper’s presentation of percentages reflecting the contribution of various factors to the advancement of SDG. It is unclear how different SDGs, which encompass diverse and sometimes incommensurable objectives, are rendered comparable or hierarchized. I did not find any robust comparability framework for assessing the relative contributions of factors to SDG. Additionally, the paper does not elucidate how these percentages correlate with macroeconomic features such as urbanization or infrastructure development, leaving a critical gap in understanding the systemic drivers of SDG outcomes.	**R4-6:** Thank you for your thoughtful comments. The primary aim of our study is to explore the complex and multidimensional relationships between nitrogen-related factors, socio-economic conditions, environmental factors, and the SDG index, rather than establishing a rigid comparability framework between the SDGs themselves. We recognize that SDGs encompass diverse and sometimes incommensurable objectives, but our focus lies in understanding how different factors contribute to SDG progress, with particular attention to their direct and indirect relationships.<br><br>Regarding urbanization, we have discussed its association with the SDG index in detail throughout the paper. As urbanization drives significant changes in infrastructure, resource efficiency, and food systems, its role is intrinsically linked to factors such as nitrogen intake and release, which ultimately influence SDG outcomes. We believe that urbanization captures many macroeconomic features related to infrastructure development and other macroeconomic drivers, which is why we did not treat infrastructure development as a separate factor in our model. Nevertheless, we have enhanced the discussion of macroeconomic factors such as urbanization and infrastructure in the results section, as they play an important role in shaping SDG progress. Examples of the revised parts of the relevant discussion:<br><br>*Line 104: “...Since the late 20th century and especially during the early 21st century, the globalization of food supply chains and urbanization have further enhanced access to animal products through improved distribution and affordability[22].”*<br><br>*Line 212: “...Urbanization also contributes positively to the SDG index (0.23), primarily through improved infrastructure and resource use efficiency with advanced technologies and management. However, urbanization also increases pollution in densely populated areas, amplifying nitrogen release in concentrated urban regions[42], with a negative impact of 0.015. At the same time, it exerts an indirect positive influence of 0.10 through nitrogen intake, reflecting its role in improving urban residents’ diets and in promoting larger agricultural land areas and farm size, which enhance agricultural productivity[43].”*<br><br>*Line 234: “...On the other hand, increased precipitation helps alleviate water scarcity, enhances the performance of water-supply infrastructure, and supports more sustainable water resource management[44]...”*<br><br>*Line 311: “...International support is also vital for addressing infrastructure gaps, while sustainable agricultural practices, such as the recycling of organic resources, can foster both environmental sustainability and poverty alleviation[15]...”*<br><br>As for the issue of comparing the contributions of different factors to SDG progress, we have acknowledged that the diverse nature of SDGs

	does pose challenges in establishing direct comparability. However, we have provided a comprehensive framework to analyze the relative influence of these factors through their correlation with the SDG index, while also considering their complex interrelationships. In the limitations section, we have added further clarification on our factor selection process and the rationale behind our approach, which we hope would address your concerns. Revised uncertainty and limitations can be found in detail in **R4-7**.
Another weakness is the absence of considerations on the interactions and combined impacts from Nitrogen and other biogeochemical elements, like phosphorus, carbon etc which together generate combined and cumulated impacts (cocktail effect). Consequently, the paper does not address the complexity and interdependence of global challenges in analysis of SDG scores. Moreover, it does not address how interlinked factors—such as climate change, economic inequality, and resource depletion—are integrated into the analysis.	**R4-7:** Thank you for your insightful comment. We fully acknowledge the limitations in factor selection in our study. However, the primary focus of this research is on nitrogen, and in some cases, factors such as nitrogen fertilizers show significant collinearity with phosphorus fertilizers. We have addressed this limitation in the limitations section, where we discuss the reasons for not considering interactions and cumulative effects between nitrogen and other biogeochemical elements like phosphorus and carbon.<br><br>Regarding climate change and economic inequality, our study's analysis of natural factors (MAT and MAP) aims to reflect the influence of climate change on SDG progress. Similarly, we capture the effects of economic inequality through the analysis of socio-economic factors such as PGDP and urbanization rates. Additionally, we conducted a classification analysis of countries with different income levels in Figure 4d to better understand how regional differences contribute to the overall SDG progress. We hope this approach helps clarify our perspective and analysis.<br><br>For other related factors such as resource depletion, we have also elaborated on their exclusion in the limitations section, providing a detailed discussion of why these aspects were not included in our study. We hope this clarifies our approach and addresses your concerns. The revised section is as follows:<br><br>*Uncertainty and limitations: "The limitations of our study primarily arise from several factors. First, the drivers considered in the analysis are selective in nature. The nitrogen intake and release parameters used in this study do not represent all possible forms of nitrogen presence, as the scope of inputs and outputs is limited. Additionally, the range of selected natural and social factors is also somewhat constrained. We selected GDP, urbanization, MAT, and MAP to ensure that the indicators are relatively independent yet complementary, thereby maintaining analytical parsimony, improving comparability, and minimizing interference from highly overlapping drivers. Alternative indicators (e.g., education attainment) could disproportionately inflate their apparent contributions to specific goals (e.g., SDG 4), whereas other dimensions, such as energy systems, natural resource use, and international trade, were not explicitly considered under the same selection principle. However, the primary objective of this study is to explore the role of key nitrogen parameters in driving SDG outcomes and to investigate the implications of the findings. This approach justifies the specific selection of factors.*

	Second, the study covers the period from 2000 to 2022, and does not account for the impacts of major global disruption events that occurred after this period. These events include the post-pandemic economic transformation, the intensification of geopolitical conflicts, and adjustments to environmental policies across various countries. All of these factors may have affected global nitrogen flow patterns, agricultural and industrial nitrogen management strategies, and pathways to achieving sustainability goals. Therefore, the conclusions drawn from historical data may have limited applicability in current and future contexts. However, this focus allows for a more focused examination of the role and contribution of nitrogen, excluding the complex influence of international politics. In the face of rapidly changing policy environments and global cooperation frameworks, the relevant conclusions and recommendations should be carefully reassessed and adjusted in light of emerging dynamics.<br><br>*Moreover, this study does not systematically examine the interactions and cumulative effects (cocktail effect) between nitrogen and other key biogeochemical elements such as phosphorus and carbon. While this single-element perspective may not fully capture the complexity of system behaviors under multiple environmental pressures, the coupled effects of nitrogen with phosphorus and carbon have been partially reflected in nitrogen management, as seen in the close coupling of nitrogen and phosphorus in fertilizers. In evaluating SDG pathways, the selected natural and social factors reflect the climatic and economic development conditions of different regions, but they do not comprehensively account for the interdependencies and synergistic effects between factors such as climate change, economic inequality, and resource depletion. In the future, we would expand the study to include more nitrogen management strategies and their broader impacts on SDGs, constructing a multi-element coupling analysis framework to more accurately reveal the systemic and holistic characteristics of sustainability challenges. Additionally, we will focus on exploring the significant roles of other elements, such as carbon and phosphorus, in driving macro-level sustainability and more fully integrate cross-element interactions, global disruption factors, and their cumulative effects on sustainable transitions."*
The section to promote the SDGs seems insufficient. Of course, policy recommendations should depend on the regional and economic context, which is mentioned in the article. But this section should explain why the analysis allows to conclude on specific policy recommendations, compare them to usual recommendations from the literature, and discuss differences.	**R4-8:** Thank you for your suggestion. We have revised the *"Promoting the SDGs from a nitrogen perspective"* section to improve its logical flow and language. In this revision, we have strengthened the connection between the research findings and policy recommendations. Specifically, we have linked the conclusions drawn from the contribution analysis to relevant policy suggestions, while also including a comparative discussion with existing literature. The revised text is as follows:<br><br>*"In low-income countries, where nitrogen intake and release have the most significant impact on SDG progress, improving access to fertilizers is crucial, as nitrogen management plays a key role in enhancing agricultural productivity. Expanding fertilizer availability and promoting NUE through precision agriculture and education are essential for improving food security and reducing malnutrition*[13].

	Additionally, by gaining insights into farmers' needs and preferences, governments can implement systematic policy interventions to improve fertilizer accessibility[14]*. This is a key strategy for enhancing agricultural productivity, increasing farmers' incomes, and ultimately reducing poverty levels. International support is also vital for addressing infrastructure gaps, while sustainable agricultural practices, such as the recycling of organic resources, can foster both environmental sustainability and poverty alleviation*[15]*. Although economic growth may present challenges, careful monitoring and regulation of fertilizer use are necessary to prevent unintended environmental damage.*<br><br>*In middle-income countries, where the contributions of various factors to SDG progress are relatively balanced, the impact of nitrogen release is more prominent in lower-middle-income countries. The primary challenge lies in balancing economic growth with environmental sustainability, particularly in regions dependent on intensive agriculture. These countries should focus on modernizing agricultural systems by integrating nutrient management strategies that combine synthetic fertilizers with organic alternatives*[16]*. Implementing pollution control frameworks, such as the European Union's National Emissions Ceilings Directive*[17]*, can mitigate nitrogen-related environmental harm. When designing policies, it is critical to ensure that smallholder farmers are not adversely affected. Providing compensation mechanisms and incentives to enhance fertilizer use efficiency is key to protecting vulnerable communities from the negative consequences of nitrogen pollution*[18]*.*<br><br>*In high-income countries, where nitrogen intake from animal-based sources plays a more prominent role, there is an opportunity to internalize agricultural externalities into food prices. This approach can encourage sustainable farming practices and dietary changes, such as reducing animal protein consumption*[19, 20]*. With access to advanced technologies and financial resources, these regions are well-positioned to enforce stringent nitrogen emission regulations. Investments in advancing NUE technologies, organic farming, and ecological practices can further reduce nitrogen's environmental footprint*[20]*. However, it is crucial that policies also support agricultural productivity and farmers' incomes, ensuring a balance between environmental goals and the economic realities of agriculture."*
The structure and clarity of the paper is also further compromised by the proliferation of numerical values within the text instead of results analysis. Moreover, the paper does not position its findings within the broader literature on food production capacity, nitrogen loss, impacts and SDG accounting.	**R4-9:** Thank you for your feedback. We acknowledge that the clarity and flow of the paper could be improved, particularly with regard to the overuse of numerical values within the text. In response to your concern, we have worked to streamline the presentation of results section and reduce the emphasis on numerical data. Instead, we have focused more on providing in-depth analysis and interpretation of the key findings, ensuring that they are clearly articulated and connected to the broader implications of nitrogen management and its role in sustainable development.<br><br>Moreover, we recognize the importance of situating our findings within the broader literature on food production capacity, nitrogen loss, and

	SDG accounting. In response to this, we have revisited the literature and incorporated relevant references to highlight how our study contributes to the ongoing discussions in these areas.
(Paragraph-specific comments)<br>(Abstract)<br>l.52: SDG 2 is justified above, but "poverty alleviation" is not justified, do you have a reference for this?	**R4-10:** Thank you for your suggestion. We have added two additional references in *Line 47* to better illustrate the role of nitrogen in agricultural production. These references further emphasize how effective nitrogen management can contribute to enhancing farmers' incomes and advancing the achievement of the Zero Hunger goal. The newly added references are as follows:<br><br>*"Erisman, J.W., Sutton, M.A., Galloway, J., Klimont, Z. & Winiwarter, W. How a century of ammonia synthesis changed the world. Nat Geosci* ***1****, 636-639 (2008). 10.1038/ngeo325*<br><br>*Zhang, X., et al. Managing nitrogen for sustainable development. Nature* ***528****, 51-59 (2015). 10.1038/nature15743"*
(Introduction)<br>The early citation of Supplementary Figure 1 in the introduction (line 5 of the introduction!) lacks contextual explanation, leaving readers to infer its relevance without guidance.	**R4-11:** Thank you for pointing out the issue regarding the premature and abrupt placement of figure citation. We have revised the text in *Line 38* to provide additional background information, ensuring that the citation and the reference to Supplementary Figure 1 are more logically integrated. The revised sentence now reads as follows:<br><br>*"…However, escalating climate change, resource scarcity, and environmental degradation have made the path toward achieving these goals by 2030 increasingly uncertain, with notable shortfalls persisting across economic prosperity, social inclusion, and environmental sustainability (Supplementary Figure 1)…"*
(Results and discussion)<br>Nitrogen Intake and Release: this section could almost be part of the introduction, as it is a primary data analysis from FAOStat, illustrated straightforward in a world map. This is a good introductory image to the topic, but is not the result of the study, which focuses of the impact of these numbers on the SDG achievement. You should be more explicit which data are directly drawn from FAOSTAT and which data were obtained after using CHANS model. Even if it was explained in the Methods section, it should be more explicit when presenting the results.	**R4-12:** Thank you for your suggestion. We understand your concern regarding the balance between "results reporting" and "data description" in the opening part of the Results section. The dietary structure and country-level food intake data are sourced from the FAO Food Balances database; however, the global dietary nitrogen intake metrics reported here are not directly taken from an existing dataset, but were recalculated in this study by converting FAO food supply data into per-capita dietary nitrogen intake. By contrast, all results on nitrogen releases are derived from CHANS model outputs. We therefore consider it appropriate to present these findings as the first part of the Results, as they summarize the empirically observed spatiotemporal patterns that underpin the subsequent analyses. Nevertheless, following your suggestion, we have added a brief clarification in *Lines 95 and 108* to make the data sources and processing steps explicit and improve transparency. The revised text is as follows:<br><br>*Line 95: "Using the Food Balances database from the Food and Agriculture Organization (FAO)*[45]*, we derived country-level per-capita dietary nitrogen intake…"*<br><br>*Line 108: "Nitrogen release estimates are based on CHANS model outputs…"*

l.77: even if it is said in the method section afterwards that data is from FAOStat, it would be helpful to remind quickly here which data allows to dray supplementary figure 2a. Strange data break in 2010, could be interesting to try to explain it.	**R4-13:** Thank you for your suggestion. We have clarified the data source for food intake at *Line 95* and in the *caption for panel a* of Supplementary Figure 2. We have also added a brief note to the caption to explain potential reasons for the apparent discontinuity around 2010 in dietary nitrogen intake. The revisions are as follows:<br><br>*Line 95: "Using the Food Balances database from the Food and Agriculture Organization (FAO)[45], we derived country-level per-capita dietary nitrogen intake…"*<br><br>*Supplementary Figure 2 (caption addition):*<br><br>*"...Nitrogen intake data was calculated based on the FAO Food Balances database (https://doi.org/10.4060/cd7162en), covering plant-based and animal-based food categories. An apparent step change around 2010 in dietary nitrogen intake may reflect updates to the FAO Food Balances series (e.g., a transition between database versions and/or revisions in balancing procedures, commodity coverage, and conversion factors), changes in country reporting and data completeness, and associated recalibration or imputation in the underlying food supply statistics. We therefore interpret this discontinuity as a potential data artifact rather than an abrupt real-world shift..."*
l.87: same here, what data is used for figure 2b ? Indicate it shortly on the figure or in the text. (probably data output from the CHANS model)	**R4-14:** Thank you for your suggestion. We have added the data source for *panel b* in the caption of Supplementary Figure 2, clarifying that the nitrogen release estimates are derived from CHANS model outputs. The revised text is as follows:<br><br>*"…Nitrogen release data is based on the CHANS model outputs."*
l.116: maybe a comment on the absolute value of nitrogen pollution in high income countries could be relevant? Even if some reductions were achieved, the levels of nitrogen release are still high.	**R4-15:** Thank you for your suggestion. We have revised the text at *Line 147* to clarify that, although high-income countries have achieved notable reductions, their nitrogen releases remain high in absolute per-capita terms. We also added representative per-capita values to support this point. The revised text is as follows:<br><br>*"…Yet, nitrogen releases in many high-income nations remain substantial due to persistently high resource demand and production intensity[23]. For example, in 2022, per-capita nitrogen release was 38 kg in North America and 24 kg in Europe, both above the global average..."*
(Driving Pathways:)<br>l.158-186: several concepts are confusing in the analysis:<br>- First, you present SDG index as determined by a combination of independent factors (Nitrogen, social and natural). However Nitrogen variables are then	**R4-16:** Thank you for your insightful comment. The focus of our study is to explore the drivers and contributions of nitrogen-related factors, socio-economic conditions, and environmental factors on the SDG index. These three factors are objectively independent or exhibit relatively low collinearity. In this section, we present the results from the Structural Equation Modeling (SEM), which not only examines the direct effects of these factors on the SDG index but also explores their indirect relationships. This is the main purpose of using SEM in our

presented as dependent on social and natural variables – which is understandable – but this is in contradiction with these variables being independent in the first place.	analysis. We recognize that the original presentation of this analysis was not clear and may have caused some confusion. Therefore, we have reorganized this section to discuss both the direct effects of the three factors and their indirect effects through nitrogen intake and nitrogen release, as mediated by socio-economic and natural factors. The aim is to provide a more comprehensive analysis of nitrogen management strategies and offer a holistic perspective for future nitrogen-related policy recommendations. We believe this is not a contradiction but rather a more nuanced analytical approach, and we hope this revision addresses your concerns. The modifications are as follows:<br><br>*Lines 194-241: "Our SEM analysis (Fig. 3a) reveals the complex multivariate relationships between nitrogen-related factors, socio-economic conditions, environmental factors, and their collective influence on the SDG index. Nitrogen intake from plant-based and animal-based foods shows a significant positive correlation with the SDG index (standardized path coefficient of 0.46). This direct relationship highlights the role of agricultural nitrogen in supporting food production systems, thereby contributing to broader SDG progress[46]. In contrast, nitrogen release shows a direct negative correlation with the SDG index (-0.10), indicating that increasing nitrogen losses are associated with declining SDG progress, particularly within the environmental domain.*<br><br>*Economic growth has a positive direct impact on the SDG index, and its influence is further mediated through nitrogen intake and nitrogen release. The indirect contributions through nitrogen intake and nitrogen release are 0.23 and -0.026, respectively, with the indirect contribution through nitrogen intake accounting for 73% of the total impact. Economic growth, driven by increased resource demand, particularly in food production and industry, leads to higher nitrogen intake. However, higher resource consumption and waste generation also result in increased nitrogen release. While economic growth supports SDG progress, its environmental impacts require mitigation through sustainable production methods[47]. Urbanization also contributes positively to the SDG index (0.23), primarily through improved infrastructure and resource use efficiency with advanced technologies and management. However, urbanization also increases pollution in densely populated areas, amplifying nitrogen release in concentrated urban regions[42], with a negative impact of 0.015. At the same time, it exerts an indirect positive influence of 0.10 through nitrogen intake, reflecting its role in improving urban residents' diets and in promoting larger agricultural land areas and farm size, which enhance agricultural productivity[43].*<br><br>*Natural factors further complicate these relationships. Climate change poses a significant threat to global sustainable development, with a strong negative direct correlation with the SDG index (-0.34). Global warming disrupts climate stability, exacerbates the risk of natural disasters, and threatens food systems[28]. It also affects nitrogen intake, resulting in a reduction of -0.12, consistent with evidence that global warming alone could reduce crop yields by 21%[28]. Additionally, the bidirectional influences of reactive nitrogen on climate cannot be*

	overlooked, with both warming effects form N_2O emission and cooling effects form NO_x and NH_3 emission through aerosol formation[37]*, and the warming influence of nitrogen emission significantly outweighs the regional cooling effect on a global scale*[38]*.*<br><br>*The impact of precipitation on SDG index is more complex. Altered precipitation patterns change the form, magnitude, and timing of nitrogen losses, resulting in indirect negative impacts through nitrogen release. On one hand, increased precipitation can intensify nitrogen leaching and runoff, reducing soil nitrogen availability and constraining crop growth, resulting in an indirect impact of -0.03. On the other hand, increased precipitation helps alleviate water scarcity, enhances the performance of water-supply infrastructure, and supports more sustainable water resource management*[44]*. Studies have found that heavy precipitation events would increase nitrogen loads in U.S. water bodies by about 20% by the end of the century*[29]*, with future hotspots of agricultural nitrogen loss expected in China, Southeast Asia, East Africa, and Brazil*[30]*. Therefore, although precipitation poses challenges from an agricultural perspective, its net influence on the SDG index is positive (0.19), as it contributes to progress toward water-related goals."*
(Driving Pathways:)<br>- Second, you present the relations between all variables as causality (e.g. l.160: "exerts a strong positive direct effect"), but does the study highlight correlations or actual causality effects ? (earlier l.139: "correlates positively"). Some clarification would be welcome on that point.	**R4-17:** Thank you for your suggestion. This study focuses on revealing the correlations between variables. Based on your recommendation, we have reviewed the entire results section and replaced all expressions that emphasize causality with more appropriate correlational language. For example, in *Line 173*, *"a stronger effect"* was replaced with *"a stronger correlation"*, *Line 196*, *"exerts a strong positive direct effect on"* was replaced with *"shows a significant positive correlation with"*, to enhance the clarity of the results section.<br><br>Furthermore, in the methodology section, we have also clarified that the study emphasizes correlation. The revision is as follows:<br><br>*Line 460: "While SEM can be used to test causal relationships, it is important to clarify that this study focuses on correlations rather than directly establishing causal pathways between variables..."*
(Contribution to the SDGs:)<br>l.195: same as above, it should be very clear if the PCA analysis highlights correlations between the studied variables, or if you really speak of causality here.	**R4-18:** Thank you for your suggestion. As per the changes in **R4-16**, the PCA in this study was primarily used for dimensionality reduction and to extract the main components from the data, helping to identify correlations between variables and further evaluate their contribution. To avoid any misunderstanding, we have replaced *"the most influential driver"* in *Line 250* with *"the most key driver."*
(Contribution to the SDGs:)<br>l.217: a suggestion here, fig. 4d might be easier to read if the bar chart was regrouped per income class instead of per drivers.	**R4-19:** Thank you for your suggestion. We have reorganized the bar chart in Fig. 4d by income groups, as per your recommendation. The revised Fig. 4 is as follows:

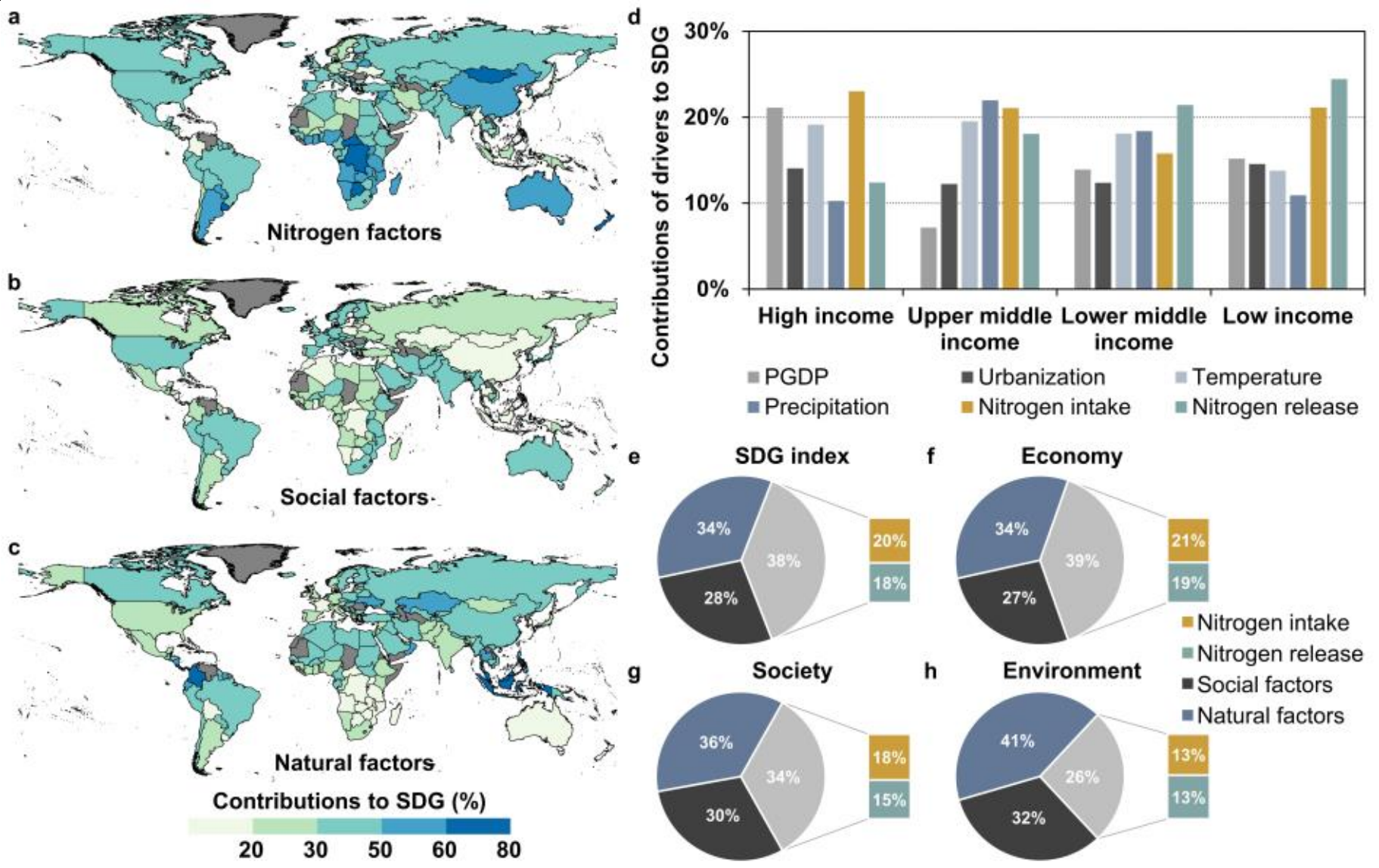

Fig. 4. Contribution of nitrogen-related, social, and natural factors to SDG index performance.

(Contribution to the SDGs:)
In the limits of the approach, the following questions could be treated more deeply:
- Is the SDG index entirely captured by the three dimensions Nitrogen/Social/Natural, and which critical dimensions could better explain the observed trends ? (e.g. energy, natural resources, national policies and international trades/geopolitical relationships)

R4-20: Thank you for your suggestion. We selected the three broad dimensions, nitrogen-related, socio-economic, and natural-condition factors, because the primary aim of this study is to quantitatively assess the associations and relative contributions of nitrogen-related factors to SDG performance. To maintain analytical parsimony, enhance comparability, and reduce interference from highly overlapping drivers, we focused on three relatively distinct dimensions with complementary coverage.

Following your recommendation, we have also expanded the *Limitations* section to clarify why other dimensions (e.g., energy, natural resources, international trade, and fluxes of other elements) were not explicitly included, in order to improve the completeness of the manuscript. The revised text is as follows:

"The limitations of our study primarily arise from several factors. First, the drivers considered in the analysis are selective in nature. The nitrogen intake and release parameters used in this study do not represent all possible forms of nitrogen presence, as the scope of inputs and outputs is limited. Additionally, the range of selected natural and social factors is also somewhat constrained. We selected GDP, urbanization, MAT, and MAP to ensure that the indicators are relatively independent yet complementary, thereby maintaining analytical parsimony, improving comparability, and minimizing interference from highly overlapping drivers. Alternative indicators (e.g., education attainment) could disproportionately inflate their apparent contributions to specific goals (e.g., SDG 4), whereas other dimensions, such as energy systems, natural resource use, and international trade, were not explicitly considered under the same selection principle. However, the primary objective of this study is to explore the role of key nitrogen parameters in driving SDG outcomes and to investigate the implications of the findings. This approach justifies the specific selection of factors.

	Second, the study covers the period from 2000 to 2022, and does not account for the impacts of major global disruption events that occurred after this period. These events include the post-pandemic economic transformation, the intensification of geopolitical conflicts, and adjustments to environmental policies across various countries. All of these factors may have affected global nitrogen flow patterns, agricultural and industrial nitrogen management strategies, and pathways to achieving sustainability goals. Therefore, the conclusions drawn from historical data may have limited applicability in current and future contexts. However, this focus allows for a more focused examination of the role and contribution of nitrogen, excluding the complex influence of international politics. In the face of rapidly changing policy environments and global cooperation frameworks, the relevant conclusions and recommendations should be carefully reassessed and adjusted in light of emerging dynamics.<br><br>*Moreover, this study does not systematically examine the interactions and cumulative effects (cocktail effect) between nitrogen and other key biogeochemical elements such as phosphorus and carbon. While this single-element perspective may not fully capture the complexity of system behaviors under multiple environmental pressures, the coupled effects of nitrogen with phosphorus and carbon have been partially reflected in nitrogen management, as seen in the close coupling of nitrogen and phosphorus in fertilizers. In evaluating SDG pathways, the selected natural and social factors reflect the climatic and economic development conditions of different regions, but they do not comprehensively account for the interdependencies and synergistic effects between factors such as climate change, economic inequality, and resource depletion. In the future, we would expand the study to include more nitrogen management strategies and their broader impacts on SDGs, constructing a multi-element coupling analysis framework to more accurately reveal the systemic and holistic characteristics of sustainability challenges. Additionally, we will focus on exploring the significant roles of other elements, such as carbon and phosphorus, in driving macro-level sustainability and more fully integrate cross-element interactions, global disruption factors, and their cumulative effects on sustainable transitions."*
(Contribution to the SDGs:)<br>- Are the Social/Natural dimensions correctly captured by the selected indicators (GDP, urbanization, MAT, MAP) and would the results vary significantly when selecting different indicators? (e.g. education, employment, or extreme weather events occurrence)	**R4-21:** Thank you for your suggestion. As noted in **R4-19**, we focused on three relatively independent yet complementary dimensions (nitrogen-related, socio-economic, and natural-condition factors) to keep the analysis parsimonious, enhance cross-country comparability, and reduce interference from highly collinear drivers. Within this framework, GDP per capita and the urbanization rate were selected as concise proxies for socio-economic development, while MAT and MAP were chosen as broadly relevant indicators of natural conditions.<br><br>Using alternative indicators (e.g., education attainment or employment) could disproportionately amplify the socio-economic contribution to specific goals such as SDG 4 or SDG 8. Similarly, using metrics such as the frequency of extreme events could narrow the representativeness of the natural-condition dimension for overall SDG progress. Because

	indicator choice can materially affect the relative contributions and thus the objectivity of cross-region comparisons, we adopted a set of widely applicable and complementary variables that best aligns with the scope of this study. We have also added this clarification to the *Limitations* section. The revised text is as follows:<br><br>*"…Additionally, the range of selected natural and social factors is also somewhat constrained. We selected GDP, urbanization, MAT, and MAP to ensure that the indicators are relatively independent yet complementary, thereby maintaining analytical parsimony, improving comparability, and minimizing interference from highly overlapping drivers. Alternative indicators (e.g., education attainment) could disproportionately inflate their apparent contributions to specific goals (e.g., SDG 4), whereas other dimensions, such as energy systems, natural resource use, and international trade, were not explicitly considered under the same selection principle. However, the primary objective of this study is to explore the role of key nitrogen parameters in driving SDG outcomes and to investigate the implications of the findings. This approach justifies the specific selection of factors."*
(Promoting the SDGs from a nitrogen perspective:)<br>The policy recommendations derived from the study seem overly simple in this paragraph and miss some primordial policy recommendations. Also, there are always risks associated with certain policies, which should be mentioned as well. Of course, these recommendations should be tailored to the economic and regional context of the area as done in the paper. But from current literature, the following recommendations are relevant (see examples below), and it could be interesting to explain whether your analysis justifies similar conclusions or not. For example, why does the analysis not mention management of organic fertilizer sources?<br><br>Examples of policy recommendations:<br>Low-Income Countries:<br>Increase access to affordable fertilizers and improve nitrogen use efficiency (NUE) through education and extension services.<br>Promote site-specific nutrient management and recycling of	**R4-22:** Thank you for your detailed suggestions on the policy implications of this study. In response, and grounded in our empirical findings, we have carefully revised, expanded, and strengthened the nitrogen-related policy recommendations, together with a clearer discussion of the associated risks and trade-offs. The revised text is as follows:<br><br>*"Achieving the SDGs, particularly with regard to nitrogen management, requires tailored strategies that consider the specific environmental conditions and developmental stages of each region. Given the varying contributions of nitrogen, socioeconomic factors, and climate to SDG progress across different areas, it is essential to adopt region-specific approaches. A one-size-fits-all policy is inadequate to address the diverse challenges associated with nitrogen use in different contexts.*<br><br>*In low-income countries, where nitrogen intake and release have the most significant impact on SDG progress, improving access to fertilizers is crucial, as nitrogen management plays a key role in enhancing agricultural productivity. Expanding fertilizer availability and promoting NUE through precision agriculture and education are essential for improving food security and reducing malnutrition[13]. Additionally, by gaining insights into farmers' needs and preferences, governments can implement systematic policy interventions to improve fertilizer accessibility[14]. This is a key strategy for enhancing agricultural productivity, increasing farmers' incomes, and ultimately reducing poverty levels. International support is also vital for addressing infrastructure gaps, while sustainable agricultural practices, such as the recycling of organic resources, can foster both environmental sustainability and poverty alleviation[15]. Although economic growth may present challenges, careful monitoring and regulation of fertilizer use are necessary to prevent unintended environmental damage.*

organic sources (e.g., manure). Support smallholder farmers with knowledge-based management and locally adapted practices.<br>Risks: Over-application as economies grow can quickly lead to pollution if not managed.<br>Middle-Income Countries<br>Implement integrated nutrient management, combining synthetic and organic sources. Remove blanket fertilizer subsidies and instead incentivize efficiency improvements and recycling. Invest in technologies like enhanced-efficiency fertilizers and precision application. Develop compensation mechanisms for smallholder farmers affected by policy changes (e.g., fertilizer taxes).<br>Risks: Policy changes can impact food prices and farmer livelihoods if not carefully designed.<br>High-Income Countries<br>Tighten regulations on fertilizer use, promote extensification, and internalize environmental costs in food prices. Encourage dietary shifts (less animal protein), food waste reduction, and circular nutrient flows. Support agroecological and organic practices, and invest in advanced technologies for NUE.<br>Risks: Overly strict policies may reduce yields or farm income if not balanced with market incentives.<br><br>(references: Zhang X. – Nature – 2015 (528[7580]) ; Wang X. – Sustainability Science – 2023 (18[3]) ; You L. – Nature Communications – 2023 (14[1]) ; Stevens C.J. – Science – 2019 (363[6427]) ; Guo Y. – Nature Food – 2020 (1[10]) ; Billen G. – One Earth – 2021 (4[6]) ; Cui Z. – Nature – 2018 (555[7696]) ;	*In middle-income countries, where the contributions of various factors to SDG progress are relatively balanced, the impact of nitrogen release is more prominent in lower-middle-income countries. The primary challenge lies in balancing economic growth with environmental sustainability, particularly in regions dependent on intensive agriculture. These countries should focus on modernizing agricultural systems by integrating nutrient management strategies that combine synthetic fertilizers with organic alternatives*[16]*. Implementing pollution control frameworks, such as the European Union's National Emissions Ceilings Directive*[17]*, can mitigate nitrogen-related environmental harm. When designing policies, it is critical to ensure that smallholder farmers are not adversely affected. Providing compensation mechanisms and incentives to enhance fertilizer use efficiency is key to protecting vulnerable communities from the negative consequences of nitrogen pollution*[18]*.*<br><br>*In high-income countries, where nitrogen intake from animal-based sources plays a more prominent role, there is an opportunity to internalize agricultural externalities into food prices. This approach can encourage sustainable farming practices and dietary changes, such as reducing animal protein consumption*[19, 20]*. With access to advanced technologies and financial resources, these regions are well-positioned to enforce stringent nitrogen emission regulations. Investments in advancing NUE technologies, organic farming, and ecological practices can further reduce nitrogen's environmental footprint*[20]*. However, it is crucial that policies also support agricultural productivity and farmers' incomes, ensuring a balance between environmental goals and the economic realities of agriculture.*<br><br>*Effective nitrogen management is central to advancing the SDGs. By improving NUE across the whole chain, mitigating emissions, and addressing the environmental and economic impacts of nitrogen pollution, countries can make significant progress toward achieving these goals*[21]*. However, managing potential risks and ensuring that policies benefit all sectors, particularly vulnerable communities, is essential. International cooperation and knowledge sharing are crucial to supporting these efforts, making nitrogen management a key component of global sustainability. A balanced approach that addresses environmental, social, and economic needs will contribute to a more sustainable and equitable future for all."*

Xia L. – Global Change Biology – 2017 (23[5]) ; Maaz T.M. – Global Change Biology – 2021 (27[8]) ; Leip A. – Global Food Security – 2020 (28) ; van Grinsven H.J.M. – Environmental Research Letters – 2015 (10[2]) ; Gu B. – Environmental Science & Technology – 2019 (53[15])	
(Promoting the SDGs from a nitrogen perspective:)<br>L.246-247: maybe the first step to mitigate the impact of nitrogen pollution is to mitigate the pollution itself with nitrogen management strategies for better NUE, before health caretaking (which is of course important too).	**R4-23:** Thank you for your suggestion. We have refined the wording in this section and, following your recommendations in **R4-21**, revised the policy implications for low-income countries accordingly. The revised text is as follows:<br><br>*"In middle-income countries, where the contributions of various factors to SDG progress are relatively balanced, the impact of nitrogen release is more prominent in lower-middle-income countries. The primary challenge lies in balancing economic growth with environmental sustainability, particularly in regions dependent on intensive agriculture. These countries should focus on modernizing agricultural systems by integrating nutrient management strategies that combine synthetic fertilizers with organic alternatives[16]. Implementing pollution control frameworks, such as the European Union's National Emissions Ceilings Directive[17], can mitigate nitrogen-related environmental harm. When designing policies, it is critical to ensure that smallholder farmers are not adversely affected. Providing compensation mechanisms and incentives to enhance fertilizer use efficiency is key to protecting vulnerable communities from the negative consequences of nitrogen pollution[18]."*
(Methods:)<br>l.271: what do you mean by "indicator"? unclear formulation	**R4-24:** Thank you for your suggestion. The term "indicator" was redundant in this context, and we have removed it.
(Methods:)<br>l.276: Supplementary tables 6 and 7 are not necessarily needed as plant-based and animal-based food categories are already presented in the FAOStat database.	**R4-25:** Thank you for your suggestion. We have removed Supplementary Tables 6 and 7 from the Supplementary Information.
(Methods:)<br>Data sources: it would be good to have precise links or DOIs to have easy and direct access to the data used for this study.	**R4-26:** Thank you for your suggestion. We have added the corresponding URLs or DOIs for all data sources in the *"Data sources"* section to improve transparency and readability. The revised text is as follows:<br><br>*"We categorized the data into three main categories: nitrogen-related factors, social factors, and natural factors. The data spans from 2000 to 2022, covering a total of 23 years of panel data across 166 countries. The nitrogen intake data used in this study is derived from the Food Balances database of the United Nations Food and Agriculture Organization (FAO, https://doi.org/10.4060/cd7162en)[45], which*

	provides detailed protein supply records on plant-based and animal-based food consumption categories.<br><br>*Full-system nitrogen release estimates were derived primarily from the CHANS mass-balance framework*[39]*. Inputs used to parameterize CHANS were assembled from three main sources. First, national- or regional-level socio-economic and activity data were compiled from global statistical sources (e.g., FAOSTAT (https://www.fao.org/faostat/en/), the World Bank (https://data.worldbank.org/), and IFASTAT (https://www.ifastat.org/databases)), covering population and economic activity, land use, agricultural production and management (including cropland area, fertilizer use, crop yields, livestock numbers, and manure management), transport activity, biomass burning, and wastewater treatment. Second, non-agricultural activity data (e.g., fuel consumption) were taken from the International Energy Agency (IEA) World Energy Balances (https://www.iea.org/data-and-statistics/data-product/world-energy-balances). Third, parameters and emission factors were obtained from the recent literature (field experiments and meta-analyses) and national emission inventories, including variables such as crop nitrogen content, harvest indices, manure recycling rates, and region-specific emission factors; where country-specific factors were unavailable, globally documented default values were applied to ensure consistent accounting. The data sources for each subsystem are detailed in Supplementary Method 3.*<br><br>*To support the compilation, harmonization, and cross-checking of sectoral emission information, we drew on several widely used global emission datasets (Supplementary Figure 4), including the Community Earth atmospheric Data System (CEDS, https://www.pnnl.gov/projects/ceds)*[48]*, the Emissions Database for Global Atmospheric Research (EDGAR, https://edgar.jrc.ec.europa.eu/)*[49]*, the Integrated Model to Assess the Global Environment (IMAGE, https://www.pbl.nl/en/image/home) framework*[50]*, and the Model of Agricultural Production and its Impact on the Environment (MAgPIE, https://www.pik-potsdam.de/en/institute/departments/activities/land-use-modelling/magpie)*[51]*.*<br><br>*Socio-economic variables were obtained from the World Bank database*[52] *(https://data.worldbank.org/), from which per capita GDP (measured in 2021 USD equivalents) and urbanization rates for each country are calculated using total GDP and population statistics. Natural-condition variables were derived from the most recent Copernicus Climate Change Service Information 2025 published by the European Union's Copernicus Earth Observation Programme*[53] *(https://climate.copernicus.eu/), which was used to extract and calculate the annual average temperature and annual average precipitation for each country."*
(Methods:)<br>National Scale Nitrogen Budget: the 3d paragraph about data	**R4-27:** Thank you for your suggestion. We have integrated the third paragraph of the "National-scale nitrogen budget" into the "Data sources" section, which makes the structure of this part of the methods

sources could be combined with the previous "Data Sources" section, in a clearer comprehensive section. It is unclear overall which data sources are used to feed which section of the CHANS model. It would be appreciated to have a clear DOI for each data source, associated with the specific use made of these data sources.	more comprehensive and clearer. Additionally, we have added DOIs for each data source in the "Data sources" section. Based on your feedback on the integration of data sources with CHANS model inputs, we have optimized the expression in *Lines 357-381* to clarify the linkage between the data sources and the model inputs. Furthermore, we have included additional discussions regarding the CHANS model in *Supplementary Method 1*. The revision to the Methods section is as follows:<br><br>*"Full-system nitrogen release estimates were derived primarily from the CHANS mass-balance framework[39]. Inputs used to parameterize CHANS were assembled from three main sources. First, national- or regional-level socio-economic and activity data were compiled from global statistical sources (e.g., FAOSTAT (https://www.fao.org/faostat/en/), the World Bank (https://data.worldbank.org/), and IFASTAT (https://www.ifastat.org/databases)), covering population and economic activity, land use, agricultural production and management (including cropland area, fertilizer use, crop yields, livestock numbers, and manure management), transport activity, biomass burning, and wastewater treatment. Second, non-agricultural activity data (e.g., fuel consumption) were taken from the International Energy Agency (IEA) World Energy Balances (https://www.iea.org/data-and-statistics/data-product/world-energy-balances). Third, parameters and emission factors were obtained from the recent literature (field experiments and meta-analyses) and national emission inventories, including variables such as crop nitrogen content, harvest indices, manure recycling rates, and region-specific emission factors; where country-specific factors were unavailable, globally documented default values were applied to ensure consistent accounting. The data sources for each subsystem are detailed in Supplementary Method 3.*<br><br>*To support the compilation, harmonization, and cross-checking of sectoral emission information, we drew on several widely used global emission datasets (Supplementary Figure 4), including the Community Earth atmospheric Data System (CEDS, https://www.pnnl.gov/projects/ceds)[48], the Emissions Database for Global Atmospheric Research (EDGAR, https://edgar.jrc.ec.europa.eu/)[49], the Integrated Model to Assess the Global Environment (IMAGE, https://www.pbl.nl/en/image/home) framework[50], and the Model of Agricultural Production and its Impact on the Environment (MAgPIE, https://www.pik-potsdam.de/en/institute/departments/activities/land-use-modelling/magpie)[51]."*
(Methods:)<br>l.314-318: can you add the references to these "Recently published field experiments, […] inventories" ?	**R4-28:** Thank you for your suggestion. Since this section involves data sources from different subsystems in the model, we have provided a detailed explanation of the data sources and their application in *Supplementary Method 3*. Following your recommendation, we have added a citation to the appendix at *Line 371* to improve the readability of the manuscript. The revised text is as follows:<br><br>*"…The data sources for each subsystem are detailed in Supplementary*

	Method 3.”
(Methods:)<br>Statistical analysis: overall, it is very hard to understand the work described in this section: many analyses and models are described, but it is not completely explicit what data intake is used, what is the exact goal of the model. There is a clear gap in the definition of the terms and a lack of clarity in the explanation of the approach and goal for each step of this study.	**R4-29:** Thank you for your suggestion. We have revised the *“Statistical analysis”* section to make the methodology of each step more accurate and clearer. The revised content now explicitly defines the data inputs for each step and the specific objectives of each model, while also refining the definitions of the terms. The revision is as follows:<br><br>*“Multiple regression analysis. To assess the impact of nitrogen-related factors on the SDG index, we used multiple regression analysis. The four parameters considered are: per capita plant-based nitrogen intake, per capita animal-based nitrogen intake, per capita nitrogen release to air, and per capita nitrogen release to water. These variables were chosen based on their direct and indirect relationships to both environmental and socio-economic SDGs, and were analyzed using multiple regression with STATA/MP 18.0 software. The regression model was formulated as follows:*<br><br>$SDG\ \ index = \alpha \cdot N_{plant} + \beta \cdot N_{animal} + \gamma \cdot N_{air} + \delta \cdot N_{water} + \varepsilon + \theta_1 + \theta_2$ *(1)*<br><br>*where* α, β, γ, *and* δ *represent the coefficients calculated through regression analysis;* ε *is the constant term;* θ_1, θ_2 *are the error terms.* N_{plant} *represents the nitrogen content in per capita plant-based food intake,* N_{animal} *represents the nitrogen content in per capita animal-based food intake,* N_{air} *represents the nitrogen content in per capita atmospheric nitrogen emissions, and* N_{water} *represents the nitrogen content in per capita nitrogen loss to water.*<br><br>*The model uses panel data from 2000 to 2022, which includes nitrogen intake, nitrogen release, and the SDG index for 166 countries. All nitrogen-related factors are expressed in kg of nitrogen per capita per year. To control for time-invariant national factors (e.g., long-term political policies, governance measures) that could influence nitrogen levels, we included a national-level intercept term* (θ_1)*. Additionally, to account for global temporal trends (e.g., economic crises, political events), we included a year intercept term* (θ_2)*. The objective of this regression analysis is to evaluate the influence of nitrogen-related variables on SDG progress, specifically to quantify the strength and direction of their relationships with the SDG index. This analysis provides insight into how different forms of nitrogen consumption and release contribute to the advancement of the SDGs.*<br><br>*Structural equation modeling. SEM was used to explore the complex relationships between nitrogen-related factors, socio-economic factors, environmental conditions, and the SDG index. SEM is an effective statistical tool for analyzing the interdependencies between multiple variables, including both observed (explicit) and latent (unobserved) variables. It provides a comprehensive understanding of both direct and indirect effects among factors, offering a more nuanced and holistic view of the relationships involved. Standardized path coefficients are used to represent the values derived from the path analysis in SEM*[35, 41].

	These coefficients indicate the strength and direction of relationships between variables in the model. They are standardized, meaning they are scaled within a range from -1 to 1, making it possible to compare different variables regardless of their original units. Path coefficients close to 1 or -1 indicate a strong relationship, while coefficients near 0 suggest a weak or no direct relationship. These coefficients help clearly identify the magnitude and direction of each relationship in the model, enabling comparisons of different paths within the model and providing insight into the overall impact of one variable on another. This makes them a key tool for assessing the contributions of various independent variables to the dependent variable and for understanding the structure of the correlation network.<br><br>*While SEM can be used to test causal relationships, it is important to clarify that this study focuses on correlations rather than directly establishing causal pathways between variables. The goal of using SEM in this study was to model the multivariate relationships between nitrogen-related factors, social and environmental factors, and their collective impact on SDG progress. We aimed to understand both the direct and indirect associations of nitrogen on the SDGs, as well as the influence of socio-economic and environmental factors on nitrogen-related variables, to ultimately present their direct, indirect, and net effects, with a particular focus on how socio-economic and environmental factors mediate these relationships.*<br><br>*First, we identified potential interactions among the key variables based on the theoretical framework of nitrogen's role in driving SDG outcomes, incorporating prior empirical knowledge on nitrogen's impact on agricultural productivity, food security, and environmental sustainability. After selecting the key variables, we used statistical tests to evaluate the significance of each variable's contribution to the model. Variables with insignificant contributions were progressively removed, and we compared the full model with the simplified version using goodness-of-fit statistics.*<br><br>*To assess the model fit, we employed three widely accepted indices: SRMR (Standardized Root Mean Square Residual), with a value less than 0.08 indicating a good fit; RMSEA (Root Mean Square Error of Approximation), with a value less than 0.1 indicating a good fit; and the Chi-square test, where a P-value greater than 0.05 indicates a good fit. We selected the optimal structural equation model based on the Comparative Fit Index (CFI), where a higher CFI value indicates a better fit between the observed data and the theoretical model. The data inputs for SEM included the SDG index, nitrogen-related factors, socio-economic variables (such as GDP and urbanization rates), and environmental factors (such as temperature and precipitation), all of which were standardized and temporally aligned to ensure consistency across the model. Finally, Fig. 3, Supplementary Tables 6 and 7 present the direct and indirect impacts of all factors in the model, along with the standardized path coefficients, providing a detailed explanation of the roles and interrelationships of the variables within the model.*<br><br>*The multiple regression and SEM analyses in this study provide a*

	comprehensive approach to understanding the relationships between nitrogen-related factors and SDG progress. The methods used allow for the evaluation of both direct and indirect impacts, offering valuable insights into the roles of different factors to the SDGs."
(Methods:)<br>l.322: I find no definition of the SDG index, what does it relate to? I imagine it relates to data presented here: SDG Index, but it should be more explicit in the paper.	**R4-30:** Thank you for your suggestion. To improve clarity and consistency, we have added the definition of the "SDG index" in the Introduction. The revisions are as follows:<br><br>*Line 83: "…quantifies the effects of nitrogen related drivers on the SDG index, a composite indicator of national performance across the 17 SDGs calculated as the average of individual SDG scores…*
(Methods:)<br>l.325: which exact panel data are you referring to?	**R4-31:** Thank you for your suggestion. We have clarified at *Line 432* what is meant by "panel data" in this context. The revised text is as follows:<br><br>*"The model uses panel data from 2000 to 2022, which includes nitrogen intake, nitrogen release, and the SDG index for 166 countries. All nitrogen-related factors are expressed in kg of nitrogen per capita per year…"*
(Methods:)<br>l.349: which background and knowledge are you referring to?	**R4-32:** Thank you for your suggestion. We have refined the description at *Line 469* regarding the background and knowledge underpinning the model specification. The revised text is as follows:<br><br>*"First, we identified potential interactions among the key variables based on the theoretical framework of nitrogen's role in driving SDG outcomes, incorporating prior empirical knowledge on nitrogen's impact on agricultural productivity, food security, and environmental sustainability…"*
(References:)<br>Include DOI or links for easier recovery of references	**R4-33:** Thank you for your suggestion. We have updated all references in both the manuscript and the Supplementary Information to include DOIs or persistent URLs, where available.

Reviewer #5:

Reviewer comment	**Number**, Response and Change
I co-reviewed this manuscript with one of the reviewers who provided the listed reports. This is part of the Nature Communications initiative to facilitate training in peer review and to provide appropriate recognition for Early Career Researchers who co-review manuscripts.	**R5-1:** Thank you for your comment. We have carefully revised the manuscript and supplementary information based on the feedback from all reviewers, and we hope this addresses your concerns.

References:

1. Erisman, J.W., Sutton, M.A., Galloway, J., Klimont, Z. & Winiwarter, W. How a century of ammonia synthesis changed the world. *Nat Geosci* **1**, 636-639 (2008). 10.1038/ngeo325
2. Zhang, X., *et al*. Managing nitrogen for sustainable development. *Nature* **528**, 51-59 (2015). 10.1038/nature15743
3. Stevens, C.J. Nitrogen in the environment. *Science* **363**, 578-580 (2019). 10.1126/science.aav8215
4. Schulte-Uebbing, L.F., Beusen, A.H.W., Bouwman, A.F. & de Vries, W. From planetary to regional boundaries for agricultural nitrogen pollution. *Nature* **610**, 507-512 (2022). 10.1038/s41586-022-05158-2
5. Sutton M, H.C.A.T., *et al*. Nitrogen Grasping the Challenge. (UKCEH, Edinburgh, 2019).
6. Hungate, B.A., Dukes, J.S., Shaw, M.R., Luo, Y. & Field, C.B. Nitrogen and Climate Change. *Science* **302**, 1512-1513 (2003). 10.1126/science.1091390
7. Chen, B., Zhang, X. & Gu, B. Managing nitrogen to achieve sustainable food-energy-water nexus in China. *Nat Commun* **16**, 4804 (2025). 10.1038/s41467-025-60098-5
8. MA, S., CM, H., KE, M., W, B. & C, C. Nitrogen Opportunities for Agriculture, Food & Environment: UNECE Guidance Document on Integrated Sustainable Nitrogen Management. (UK Centre for Ecology & Hydrology, Edinburgh, 2022).
9. FAO. *Sustainable nitrogen management in agrifood systems* (FAO, Rome, 2025). 10.4060/cd3388en
10. Kanter, D.R., *et al*. Nitrogen pollution policy beyond the farm. *Nat Food* **1**, 27-32 (2020). 10.1038/s43016-019-0001-5
11. Morseletto, P. Confronting the nitrogen challenge: Options for governance and target setting. *Global Environmental Change* **54**, 40-49 (2019). 10.1016/j.gloenvcha.2018.10.010
12. Kanter, D.R., Chodos, O., Nordland, O., Rutigliano, M. & Winiwarter, W. Gaps and opportunities in nitrogen pollution policies around the world. *Nat Sustain* **3**, 956-963 (2020). 10.1038/s41893-020-0577-7
13. Gu, B., *et al*. Cost-effective mitigation of nitrogen pollution from global croplands. *Nature* **613**, 77-84 (2023). 10.1038/s41586-022-05481-8
14. Ma, W., Rahut, D.B., Sonobe, T. & Gong, B. Linking farmers to markets: Barriers, solutions, and policy options. *Econ Anal Policy* **82**, 1102-1112 (2024). 10.1016/j.eap.2024.05.005
15. You, L., *et al*. Global mean nitrogen recovery efficiency in croplands can be enhanced by optimal nutrient, crop and soil management practices. *Nat Commun* **14**, 5747 (2023). 10.1038/s41467-023-41504-2
16. Guo, Y., *et al*. Air quality, nitrogen use efficiency and food security in China are improved by cost-effective agricultural nitrogen management. *Nat Food* **1**, 648-658 (2020). 10.1038/s43016-020-00162-z
17. EU. National Emission reduction Commitments Directive (2023). https://www.eea.europa.eu/themes/air/air-pollution-sources-1/national-emission-ceilings
18. Cui, Z., *et al*. Pursuing sustainable productivity with millions of smallholder farmers. *Nature* **555**, 363-366 (2018). 10.1038/nature25785
19. Billen, G., *et al*. Reshaping the European agro-food system and closing its nitrogen cycle: The potential of combining dietary change, agroecology, and circularity. *One Earth* **4**, 839-850 (2021). 10.1016/j.oneear.2021.05.008
20. van Grinsven, H.J.M., Erisman, J.W., de Vries, W. & Westhoek, H. Potential of extensification of European agriculture for a more sustainable food system, focusing on nitrogen. *Environ Res Lett* **10**, 25002 (2015). 10.1088/1748-9326/10/2/025002
21. Zhang, X., *et al*. Managing nitrogen for sustainable development. *Nature* **528**, 51-59 (2015). 10.1038/nature15743
22. Davis, K.F., Downs, S. & Gephart, J.A. Towards food supply chain resilience to environmental shocks. *Nat Food* **2**, 54-65 (2021). 10.1038/s43016-020-00196-3
23. Liu, L., *et al*. Exploring global changes in agricultural ammonia emissions and their contribution to nitrogen deposition since 1980. *Proceedings of the National Academy of Sciences* **119**, e2121998119 (2022). 10.1073/pnas.2121998119
24. Kraak, V.I. & Aschemann-Witzel, J. The Future of Plant-Based Diets: Aligning Healthy Marketplace Choices with Equitable, Resilient, and Sustainable Food Systems. *Annu Rev Public Health* **45**, 253-275 (2024). 10.1146/annurev-publhealth-060722-032021
25. Tessier, A., *et al*. Optimal dietary patterns for healthy aging. *Nat Med* **31**, 1644-1652 (2025). 10.1038/s41591-025-03570-5
26. Uwizeye, A., *et al*. Nitrogen emissions along global livestock supply chains. *Nat Food* **1**, 437-446 (2020). 10.1038/s43016-020-0113-y
27. Bai, Z., *et al*. Livestock Housing and Manure Storage Need to Be Improved in China. *Environ Sci Technol* **51**, 8212-8214

(2017). 10.1021/acs.est.7b02672
28. Cui, J., *et al*. Adaptive Mitigation of Warming-Induced Food Crisis and Nitrogen Pollution. *Environ Sci Technol* **59**, 3527-3536 (2025). 10.1021/acs.est.4c05531
29. Sinha, E., Michalak, A.M. & Balaji, V. Eutrophication will increase during the 21st century as a result of precipitation changes. *Science* **357**, 405-408 (2017). 10.1126/science.aan2409
30. Yang, Y., *et al*. Climate change exacerbates the environmental impacts of agriculture. *Science* **385**, eadn3747 (2024). 10.1126/science.adn3747
31. Turner, C., *et al*. Food Environment Research in Low- and Middle-Income Countries: A Systematic Scoping Review. *Adv Nutr* **11**, 387-397 (2020). 10.1093/advances/nmz031
32. UN. The Sustainable Development Goals Report 2022. (2022).
33. Moellendorf, D. Climate change and global justice. *WIREs Climate Change* **3**, 131-143 (2012). 10.1002/wcc.158
34. Archibugi, D. & Pietrobelli, C. The globalisation of technology and its implications for developing countries. *Technol Forecast Soc Change* **70**, 861-883 (2003). 10.1016/S0040-1625(02)00409-2
35. Xu, J., *et al*. Soil health contributes to variations in crop production and nitrogen use efficiency. *Nat Food* **6**, 597-609 (2025). 10.1038/s43016-025-01155-6
36. Abel, C., *et al*. The human–environment nexus and vegetation–rainfall sensitivity in tropical drylands. *Nat Sustain* **4**, 25-32 (2021). 10.1038/s41893-020-00597-z
37. Andreae, M.O., Jones, C.D. & Cox, P.M. Strong present-day aerosol cooling implies a hot future. *Nature* **435**, 1187-1190 (2005). 10.1038/nature03671
38. Zaehle, S., Ciais, P., Friend, A.D. & Prieur, V. Carbon benefits of anthropogenic reactive nitrogen offset by nitrous oxide emissions. *Nat Geosci* **4**, 601-605 (2011). 10.1038/ngeo1207
39. Gu, B. & Zhang, X. The Coupled Human And Natural Systems (CHANS) Nitrogen Cycling Model (2020). https://person.zju.edu.cn/bjgu#928507
40. Gu, B., Ju, X., Chang, J., Ge, Y. & Vitousek, P.M. Integrated reactive nitrogen budgets and future trends in China. *Proceedings of the National Academy of Sciences* **112**, 8792-8797 (2015). 10.1073/pnas.1510211112
41. Ren, C., *et al*. Ageing threatens sustainability of smallholder farming in China. *Nature* **616**, 96-103 (2023). 10.1038/s41586-023-05738-w
42. Lu, M., Zhou, C., Wang, C., Jackson, R.B. & Kempes, C.P. Worldwide scaling of waste generation in urban systems. *Nature Cities* **1**, 126-135 (2024). 10.1038/s44284-023-00021-5
43. Wang, S., *et al*. Global urbanization benefits food security and nature restoration. *Resources, Conservation and Recycling* **216**, 108174 (2025). 10.1016/j.resconrec.2025.108174
44. Wehbe, Y., Griffiths, S., Al Mazrouei, A., Al Yazeedi, O. & Al Mandous, A. Rethinking water security in a warming climate: rainfall enhancement as an innovative augmentation technique. *NPJ Clim Atmos Sci* **6**, 171 (2023). 10.1038/s41612-023-00503-2
45. FAOSTAT. Food Balances (2010-) (2025). https://www.fao.org/faostat/
46. Potapov, P., *et al*. Global maps of cropland extent and change show accelerated cropland expansion in the twenty-first century. *Nat Food* **3**, 19-28 (2022). 10.1038/s43016-021-00429-z
47. Loiseau, E., *et al*. Green economy and related concepts: An overview. *J Clean Prod* **139**, 361-371 (2016). 10.1016/j.jclepro.2016.08.024
48. Hoesly, R.M., *et al*. Historical (1750–2014) anthropogenic emissions of reactive gases and aerosols from the Community Emissions Data System (CEDS). *Geosci Model Dev* **11**, 369-408 (2018). 10.5194/gmd-11-369-2018
49. Crippa, M., *et al*. Insights into the spatial distribution of global, national, and subnational greenhouse gas emissions in the Emissions Database for Global Atmospheric Research (EDGAR v8.0). *Earth Syst Sci Data* **16**, 2811-2830 (2024). 10.5194/essd-16-2811-2024
50. PBL. Integrated Model to Assess the Global Environment (IMAGE) model Documentation (2020). https://models.pbl.nl/image/index.php/Welcome_to_IMAGE_3.2_Documentation
51. Dietrich, J.P., *et al*. MAgPIE - An Open Source land-use modeling framework - Version 4.0 (2018). https://rse.pik-potsdam.de/doc/magpie/4.0/

52. WB. World Bank Open Data (2025). https://data.worldbank.org/
53. EU. Copernicus Climate Change Service Information 2025 (2025). https://climate.copernicus.eu/

Point-by-point response to reviewers

Reviewer #1:

Reviewer comment	Response
I have reviewed the revised manuscript and the authors' responses to the reviewers' comments. All my previous concerns and suggestions have been satisfactorily addressed, leading to a significantly improved manuscript. I have no further comments and recommend the manuscript for publication.	We sincerely thank the reviewer for the positive evaluation of our revised manuscript and for recommending it for publication. We are grateful that the reviewer considered our previous responses and revisions satisfactory.

Reviewer #2:

**Reviewer comment**	Response
Most of the comments are addressed sufficiently.	We sincerely thank the reviewer for the constructive comments and for recognizing that most of the previous concerns have been sufficiently addressed.

Reviewer #3:

Reviewer comment	Response
I am satisfied that all my comments have been adequately addressed and would recommend acceptance of this manuscript.	We sincerely thank the reviewer for the positive assessment of our revised manuscript and for recommending acceptance. We appreciate the reviewer's recognition that the previous comments have been adequately addressed.

Reviewer #6:

**Reviewer comment**	**Number**, Response and Change
The manuscript "Quantifying the role of nitrogen in achieving global sustainable development goals" addresses a highly ambitious and interdisciplinary question. Despite the inherent complexity of linking nitrogen dynamics with broad SDG performance, including social dimensions that are not commonly integrated into nitrogen-related studies, I consider this work to be potentially valuable and conceptually interesting. In particular, the manuscript provides a broad systems-level perspective that could stimulate future research on the role of nitrogen management in sustainable development pathways.	**R6-1:** We sincerely thank the reviewer for the positive and encouraging assessment of our manuscript. We are grateful that the reviewer recognized the value and conceptual interest of addressing nitrogen dynamics within the broader SDG framework, as well as the potential of this systems-level perspective to stimulate future research on nitrogen management and sustainable development pathways.
Overall, I agree with most of the concerns raised by reviewer #4. In my opinion, reviewer #4 identified several important methodological and conceptual issues that required clarification, particularly regarding the definition of system boundaries, the integration of the CHANS framework with the statistical analyses, the distinction between causality and correlation, and the transparency of the modelling approach. I also believe that the authors have substantially improved the manuscript in response to these comments. The revised version is considerably clearer in terms of methodological traceability, model description, and interpretation of the statistical analyses.	**R6-2:** We thank the reviewer for the thoughtful evaluation of our previous revision. We appreciate the reviewer's recognition that the revised manuscript has substantially improved in terms of methodological traceability, model description, and interpretation of the statistical analyses. These aspects were central to our previous revision, and we have continued to refine them in the current version.

There are only a few points raised by reviewer #4 with which I partially disagree. First, regarding the concern about whether the “Social” and “Natural” dimensions are adequately represented by the selected indicators (GDP, urbanization, MAT, and MAP), I consider the authors’ response reasonable within the scope of the study. Although alternative indicators could certainly have been included, the authors adequately justify their selection criteria based on analytical parsimony, comparability, and reduction of collinearity among explanatory variables.	**R6-3:** We appreciate the reviewer’s balanced assessment regarding the representation of the “Social” and “Natural” dimensions. We are grateful that the reviewer considered our selection of GDP, urbanization, MAT, and MAP reasonable within the scope of the study, particularly in light of our aims of maintaining analytical parsimony, cross-country comparability, and reduced collinearity among explanatory variables.
Second, reviewer #4 questioned the novelty of the manuscript, arguing that there is already extensive literature linking nitrogen use, food security, and environmental degradation. While I agree that this literature is indeed extensive, I believe the novelty of the present study lies in its attempt to integrate nitrogen intake and release dynamics within the broader framework of all 17 SDGs, rather than focusing only on SDGs directly associated with food production or environmental pollution (e.g., SDGs 2, 6, 12, 13, 14, and 15). In this regard, I believe the revised manuscript now better contextualizes its contribution relative to previous studies.	**R6-4:** We sincerely thank the reviewer for recognizing the novelty of our study. We appreciate the reviewer’s view that the contribution of this work lies in integrating nitrogen intake and release dynamics within the broader framework of all 17 SDGs, rather than focusing only on SDGs directly linked to food production or environmental pollution. This is consistent with the central motivation of our study.

I also agree that reviewer #4 raised an important conceptual limitation regarding the exclusion of interactions with other biogeochemical elements such as phosphorus and carbon. This is indeed one of the first questions that arises when reading the manuscript. However, although this limitation remains relevant, I consider the authors' decision to maintain a nitrogen-centered framework acceptable within the scope of the study, particularly after the expanded discussion included in the revised limitations section.	**R6-5:** We thank the reviewer for this constructive comment. We agree that interactions between nitrogen and other biogeochemical elements, such as phosphorus and carbon, are important and remain a relevant limitation. We appreciate the reviewer's recognition that a nitrogen-centered framework is acceptable within the scope of the present study, particularly after the expanded discussion included in the revised limitations section.
Importantly, the comments raised by reviewer #4 did not reduce my perception of the conceptual relevance or novelty of the manuscript. On the contrary, I believe the revised version is substantially stronger because the review process improved the transparency of the data sources, clarified the modelling framework, and moderated several interpretations that were initially presented too strongly. Overall, I consider reviewer #4's comments constructive, scientifically justified, and highly valuable for improving the manuscript.	**R6-6:** We sincerely appreciate the reviewer's positive assessment that the revised manuscript is substantially stronger. We are grateful that the reviewer recognized the improvements in data-source transparency, modelling-framework clarification, and the more cautious interpretation of the statistical results. These points have also guided our additional revision in this round.

I also do not consider the additional analyses and revisions requested by reviewer #4 to be unfeasible or beyond the scope of the study. In fact, if I had reviewed the original version of the manuscript, I would likely have raised several similar concerns.	**R6-7:** We thank the reviewer for this fair and constructive assessment. We agree that Reviewer #4's comments were scientifically valuable and helped improve the manuscript. We also appreciate the reviewer's view that the additional analyses and revisions requested were feasible and within the scope of the study.
Beyond these points, I would like to add a few additional comments regarding the current structure and interpretation of the manuscript.	**R6-8:** We thank the reviewer for introducing additional comments on the current structure and interpretation of the manuscript. We have carefully considered these remaining suggestions and have addressed them point-by-point below, with corresponding revisions made where appropriate.
1. First, I agree with reviewer #4 that Figures 1 and 2 remain primarily descriptive. Although the data are normalized on a per-capita basis, these figures still appear to represent a direct visualization of underlying database trends rather than central analytical outcomes derived from the modelling framework itself. In my opinion, they function more as contextual information introducing the topic than as core results of the study. More specifically, Figures 1 and 2 appear closely connected to the information already presented in Supplementary Figure 2a and 2b, essentially reflecting a comparison between the years 2000 and 2022, although Supplementary Figure 2b additionally provides information regarding the forms of nitrogen losses. For this reason, I feel that the main figures do not substantially extend the analytical interpretation beyond the temporal trends already shown in the Supplementary Information. I believe these figures could	**R6-9:** We thank the reviewer for this constructive suggestion. To improve the balance between descriptive context and analytical results in the main manuscript, we have condensed and reorganized these figures. Specifically, we combined the key information from the original Figures 1 and 2 into *a single revised main figure*, which now presents only total dietary nitrogen intake and total nitrogen release in 2000 and 2022, together with their changes from 2000 to 2022. The more detailed component-specific maps, including plant-based and animal-based nitrogen intake and nitrogen release to air and water, have been moved to the Supplementary Information as *Supplementary Figs. 3 and 4*. In addition, all relevant figure references and subsequent figure numbering have been updated accordingly. The revised and organized figures are as follows.

potentially be condensed, incorporated more explicitly as introductory/background material, or partially transferred to the Supplementary Information in order to improve the balance between descriptive context and analytical results in the main manuscript.

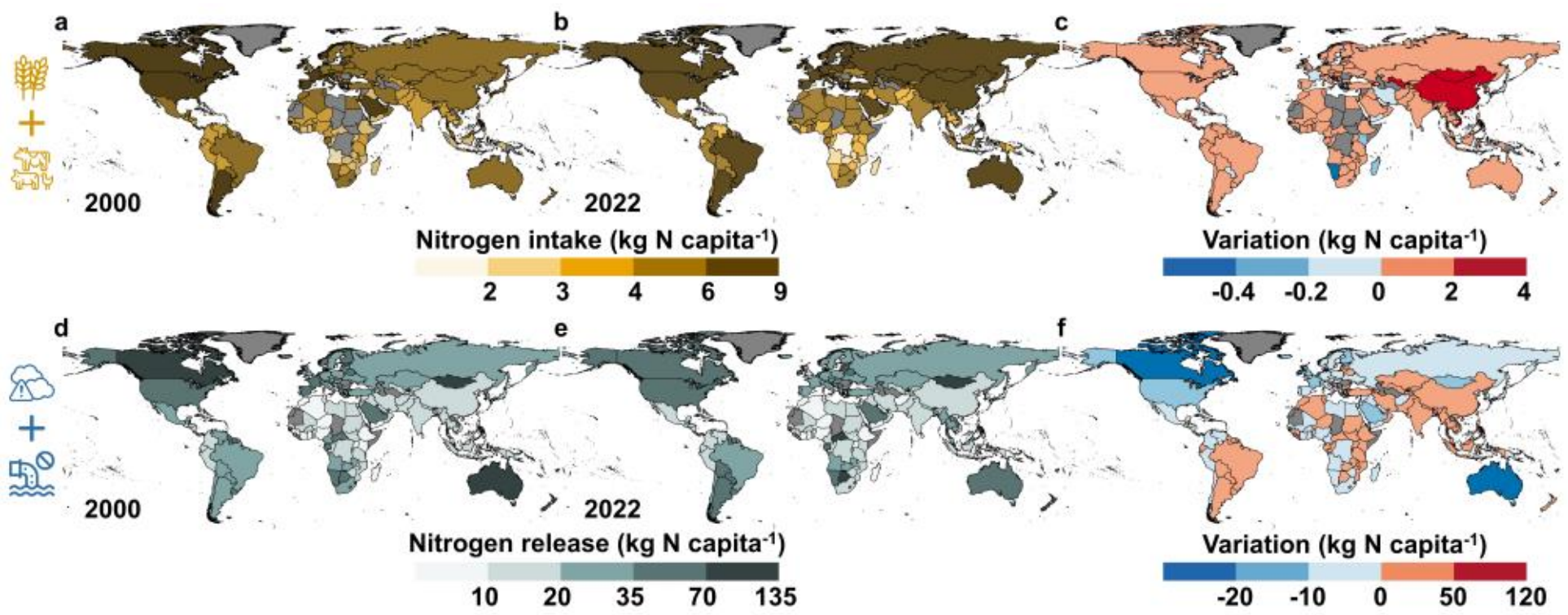

Fig. 1 | Global patterns and changes in total nitrogen intake and release from 2000 to 2022. *(a, b) Spatial distribution of total dietary nitrogen intake in 2000 and 2022, respectively. (c) Change in total dietary nitrogen intake from 2000 to 2022. (d, e) Spatial distribution of total nitrogen release to air and water in 2000 and 2022, respectively. (f) Change in total nitrogen release from 2000 to 2022. Dietary nitrogen intake was calculated from the FAO Food Balances database (https://doi.org/10.4060/cd7162en). Nitrogen release includes* NH_3*,* NO_x*, and* N_2O *emissions to air and* NO_3^- *losses to water, estimated using the CHANS model. All values are expressed as kg N capita*$^{-1}$*; changes represent values in 2022 minus values in 2000. Grey areas indicate missing data. The base map with administrative boundaries was created using data from GADM (https://gadm.org/data.html), without endorsement.*

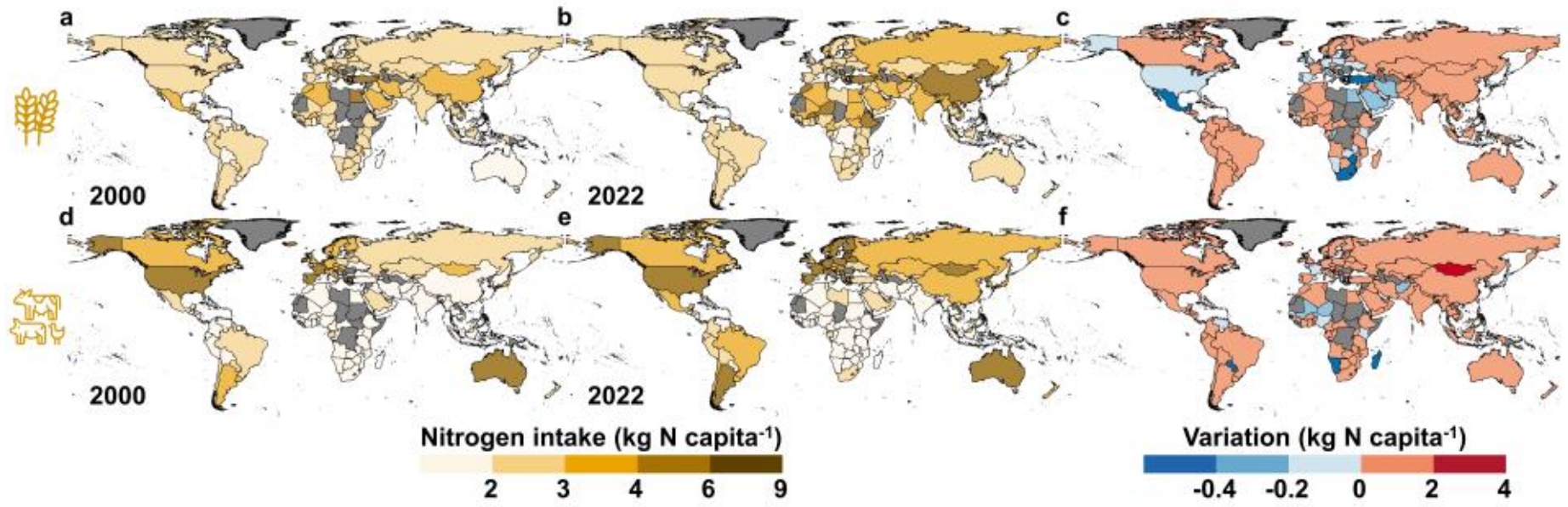

Supplementary Fig. 3 | Global patterns and changes in dietary nitrogen intake from plant- and animal-based foods from 2000 to 2022.

(a, b) Spatial distribution of nitrogen intake from plant-based food in 2000 and 2022, respectively. (c) Change in nitrogen intake from plant-based food from 2000 to 2022. (d, e) Spatial distribution of nitrogen intake from animal-based food in 2000 and 2022, respectively. (f) Change in nitrogen intake from animal-based food from 2000 to 2022. Dietary nitrogen intake was calculated from the FAO Food Balances database (https://doi.org/10.4060/cd7162en). All values are expressed as kg N capita^{-1}; changes represent values in 2022 minus values in 2000. Grey areas indicate missing data. The base map with administrative boundaries was created using data from GADM (https://gadm.org/data.html), without endorsement.

	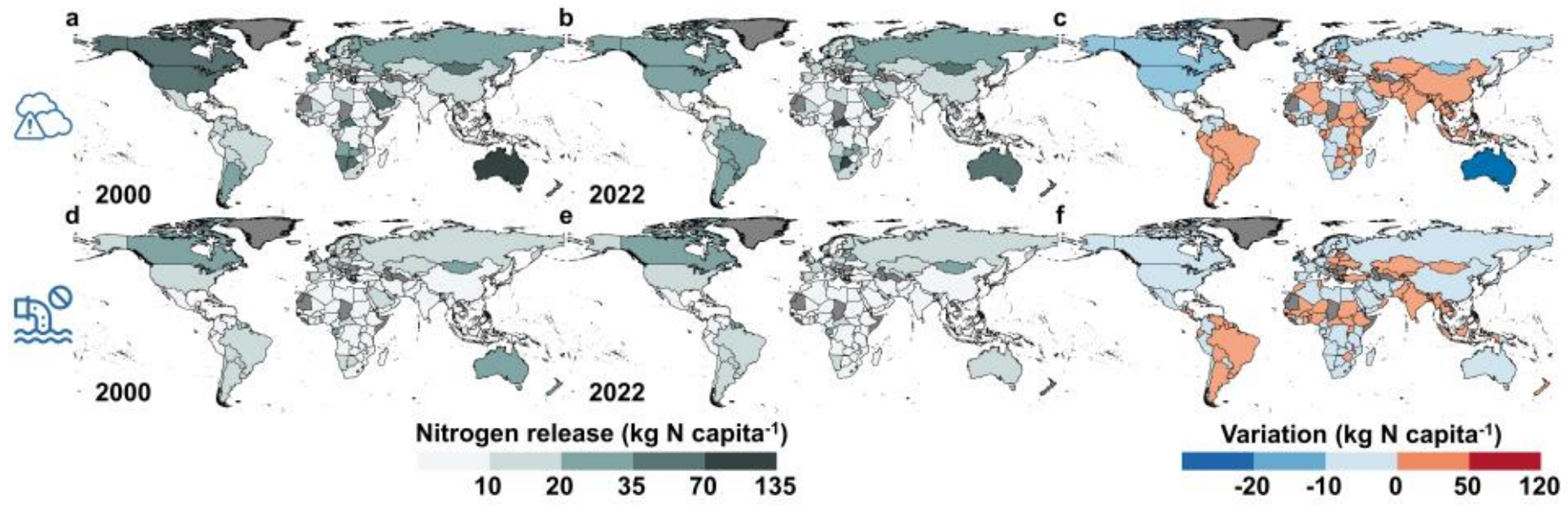<br><br>***Supplementary Fig. 4 | Global patterns and changes in nitrogen release to air and water from 2000 to 2022.***<br>*(a, b) Spatial distribution of nitrogen release to air in 2000 and 2022, respectively. (c) Change in nitrogen release to air from 2000 to 2022. (d, e) Spatial distribution of nitrogen release to water in 2000 and 2022, respectively. (f) Change in nitrogen release to water from 2000 to 2022. Nitrogen release to air includes NH_3, NO_x, and N_2O emissions, and nitrogen release to water includes NO_3^- losses through leaching and runoff, estimated using the CHANS model. All values are expressed as kg N capita^{-1}; changes represent values in 2022 minus values in 2000. Grey areas indicate missing data. The base map with administrative boundaries was created using data from GADM (https://gadm.org/data.html), without endorsement.*
2. Second, I found the transition between the “Driving pathways” and “Factors contributing to the SDGs” sections somewhat inconsistent in terms of the grouping strategy used for countries. In the “Driving pathways” section, the analyses and discussion are primarily organized by world regions, whereas in Figure 4d and in the policy-oriented section “Promoting the SDGs from a nitrogen perspective”, the	**R6-10:** We thank the reviewer for pointing out the potential inconsistency between the regional framing in the “Driving pathways” section and the income-group framing in the “Factors contributing to the SDGs” and policy sections. We agree that the analytical logic linking these two perspectives was not sufficiently explicit in the previous version. We have therefore clarified that regional and income-group classifications serve different but complementary purposes: the regional perspective captures spatial heterogeneity in dietary

interpretation shifts toward country classification by income group. In my opinion, this creates a degree of conceptual inconsistency throughout the manuscript.	patterns, agricultural systems, climatic background, and nitrogen-management contexts, whereas the income-group perspective helps interpret differences in development stage, infrastructure, governance capacity, fertilizer accessibility, and pollution-control ability. The revised text is provided in **R6-11**.
I believe this issue is particularly important because several results presented in the manuscript seem to support the idea that socio-economic development level may ultimately explain SDG-related nitrogen dynamics more consistently than broad geographical classification alone. For example, in the SEM presented in Figure 3, the relationships between socio-economic variables (particularly GDP and urbanization) and nitrogen intake/release appear substantially stronger than those associated with natural variables such as MAT and MAP. Although climatic differences clearly remain important, this pattern may suggest that the economic development level of a country exerts a stronger influence on how nitrogen dynamics interact with SDG performance.<br><br>This interpretation also appears to be consistent with the contribution analysis shown in Figure 4d. While the contribution of nitrogen intake remains relatively similar across the four income groups, the contribution of nitrogen release progressively decreases with increasing income level. This is, in my opinion, one of the most interesting results of the manuscript, because it may indicate that as countries improve the control and management of reactive nitrogen losses, the relative influence of nitrogen release on SDG performance	**R6-11:** We appreciate the reviewer's insightful observation that income level may provide an important explanatory lens for understanding nitrogen–SDG dynamics. We agree that the SEM results and contribution analysis both suggest that socio-economic development is closely associated with nitrogen intake, nitrogen release, and their relationships with SDG performance.<br><br>To address this point, we have revised three relevant sections of the manuscript. First, in the *"Driving pathways"* section, we have clarified why regional grouping is used in this part of the analysis. The revised text is as follows.<br><br>*Lines 163-166:*<br>*"We first discuss nitrogen–SDG associations by world region, because regional groupings capture spatial heterogeneity in dietary structure, agricultural systems, climatic background, and nitrogen-management context. These regional patterns provide the geographical basis for interpreting the country-level associations identified by the regression analysis…"*<br><br>Second, we have strengthened the income-group interpretation in the *"Factors contributing to the SDGs"* section. We now explicitly discuss that GDP and urbanization show strong associations with nitrogen intake and release in the SEM analysis, and that the contribution of nitrogen release decreases with increasing income level. We further explain that this pattern may reflect improved reactive nitrogen control and pollution-management capacity in higher-income countries, whereas nitrogen-related constraints remain more prominent in low- and lower-middle-income countries. The revised text is as follows.

diminishes, while other drivers, particularly climate-related factors, become proportionally more important. Interestingly, this interpretation is also consistent with the discussion already included in the revised manuscript regarding high-income countries (lines 275 to 280 in the revised version).<br><br>Furthermore, the regions showing the highest contribution percentages of nitrogen-related drivers in Figure 4a largely overlap with countries classified as low- or lower-middle-income. Similarly, Figures 2g,h,i show that the strongest positive trends in nitrogen release variation are concentrated in many of these same countries. Therefore, the income-level perspective already appears implicitly throughout multiple sections of the manuscript, including the final policy recommendations section, which is itself structured according to income categories.<br><br>For these reasons, I believe the manuscript could benefit from a more consistent analytical framing throughout the different sections, either by maintaining the regional approach more systematically or, alternatively, by strengthening the interpretation based on income-group classification across the manuscript.	*Lines 252-259:*<br>*"…This understanding requires both spatial and developmental perspectives. The regional analysis in the previous section highlights geographical heterogeneity in nitrogen intake, nitrogen release, and their associations with SDG performance. Here, we further evaluate the relative contributions of nitrogen-related, socio-economic, and natural factors at the national scale and interpret these contributions using both regional patterns and income-group classification. The regional perspective helps identify where nitrogen-related constraints are concentrated, whereas the income-group perspective reflects differences in development stage, infrastructure, governance capacity, fertilizer accessibility, and pollution-control ability."*<br><br>*And Lines 288-309:*<br>*"The income-group perspective provides an additional lens for understanding the regional patterns described above (Fig. 3d). In the SEM analysis, socio-economic variables, particularly GDP and urbanization, show strong associations with both nitrogen intake and nitrogen release, suggesting that development stage is closely linked to the way nitrogen dynamics interact with SDG performance. This interpretation is also consistent with the contribution analysis across income groups. While the contribution of nitrogen intake remains relatively similar across the four income groups, the contribution of nitrogen release decreases with increasing income level. This pattern suggests that, as countries improve pollution control and reactive nitrogen management, the relative importance of nitrogen release for SDG performance declines, whereas other drivers, especially climate-related constraints, become proportionally more prominent. For example, high-income and upper-middle-income countries have generally made substantial progress toward the SDGs and have more stable economic development, stronger environmental governance, and more advanced nitrogen-loss control. With the Zero Hunger goal largely achieved and nitrogen losses more effectively managed, the potential for further improvements through traditional socio-economic factors*

	is relatively limited[67]*. However, because of the widespread, long-term, and transboundary nature of climate risks, global warming has become an increasingly important constraint on sustainable development in these countries, particularly in relation to climate adaptation, energy transition, and international responsibility*[68]*. By contrast, in low-income countries, nitrogen-related constraints remain dominant, contributing 46%, with both agricultural nitrogen use and dietary nitrogen intake playing central roles, as basic food security and nutrition needs often take precedence over climate adaptation strategies*[69]*."*<br><br>Finally, we have revised the opening of the policy section to clarify why the recommendations are structured primarily by income group, while still acknowledging regional heterogeneity. This revision helps align the analytical framing of the results with the policy implications. The revised text is as follows.<br><br>*Lines 321-327:*<br>*"Achieving the SDGs through improved nitrogen management requires strategies that account for both regional heterogeneity and differences in development stage. Regional differences reflect diverse agricultural systems, dietary structures, climatic conditions, and nitrogen-loss pathways, whereas income groups better capture differences in fertilizer accessibility, infrastructure, governance capacity, and pollution-control ability. Therefore, the policy discussion below is structured primarily by income group, while also recognizing the regional contexts in which these nitrogen-management challenges occur."*

3. Finally, regarding the policy recommendations section, I think the revised version has improved substantially after reviewer #4's comments. However, I believe it would still be valuable to emphasize more strongly the importance of farmer education and technical support in low- and lower-middle-income countries, particularly regarding nitrogen loss mitigation and fertilizer overapplication practices. In many agricultural systems, excessive fertilizer application is still used as a strategy to "ensure yield security," despite its environmental consequences.	**R6-12:** We thank the reviewer for this valuable suggestion. We agree that farmer education and technical support deserve stronger emphasis, particularly in low- and lower-middle-income countries where excessive fertilizer application is often used as a risk-avoidance strategy to ensure yield security.<br><br>To address this point, we have revised the policy section to highlight the importance of extension services, field-based training, soil testing, nutrient budgeting, and site-specific fertilizer recommendations. We now clarify that improving fertilizer access should be accompanied by knowledge-based support so that farmers can maintain yields while avoiding unnecessary fertilizer inputs and reducing nitrogen losses to air and water. The revised text is as follows.<br><br>*Lines 329-347:*<br>*"In low-income countries, where nitrogen intake and release have the most significant impact on SDG progress, improving access to fertilizers remains crucial, as nitrogen management plays a key role in enhancing agricultural productivity. Expanding fertilizer availability and promoting NUE through precision agriculture and education are essential for improving food security and reducing malnutrition[71]. These challenges are also relevant to many lower-middle-income countries, where farmer education and technical support are particularly important for improving NUE and reducing nitrogen losses. In many smallholder systems, fertilizer overapplication is often used as a risk-avoidance strategy to ensure yield security, especially where farmers face limited access to soil testing, technical advice, stable markets, or crop insurance[72]. Therefore, expanding fertilizer availability should be accompanied by extension services, field-based training, soil testing, nutrient budgeting, and site-specific fertilizer recommendations. Additionally, by gaining insights into farmers' needs and preferences, governments can implement systematic policy interventions to improve fertilizer accessibility[73]. This is a key strategy for enhancing agricultural productivity, increasing farmers' incomes, and ultimately reducing poverty levels. International support is also vital for*

	addressing infrastructure gaps, while sustainable agricultural practices, such as the recycling of organic resources, can foster both environmental sustainability and poverty alleviation[74]. Although economic growth may present challenges, careful monitoring and regulation of fertilizer use are necessary to prevent unintended environmental damage."
I would also suggest some caution regarding the promotion of organic alternatives without discussing their potential trade-offs (line 322 in the revised version). Depending on their C:N ratio and management conditions, some organic amendments may stimulate denitrification processes and increase greenhouse gas emissions. Therefore, the manuscript could perhaps mention that improvements in nitrogen use efficiency may also involve enhanced-efficiency fertilizers or the use of crops with biological nitrification or denitrification inhibition capacity (BNI/BDI systems), which represent promising complementary approaches for reducing reactive nitrogen losses.	**R6-13:** Thanks for this important comment. We agree that organic amendments should not be presented as universally beneficial. Following your suggestions, we have revised the policy section to present organic resources more cautiously and to discuss their potential trade-offs. We have also added enhanced-efficiency fertilizers, controlled-release fertilizers, nitrification inhibitors, and crop varieties or rotations with BNI or BDI capacity as complementary approaches for improving nitrogen use efficiency and reducing reactive nitrogen losses. The revised text is as follows.<br><br>*Lines 349-367:*<br>*"In middle-income countries, where the contributions of various factors to SDG progress are relatively balanced, the impact of nitrogen release is more prominent in lower-middle-income countries. The primary challenge lies in balancing economic growth with environmental sustainability, particularly in regions dependent on intensive agriculture. These countries should focus on modernizing agricultural systems through integrated nutrient management, including the coordinated use of synthetic fertilizers and organic resources where locally appropriate[75]. However, organic amendments are not risk-free, and their outcomes depend on management conditions such as carbon-nitrogen ratio, application rate and timing, soil moisture, and aeration. If poorly managed, some organic amendments may stimulate denitrification and increase greenhouse gas emissions[76]. Therefore, improvements in NUE should also include complementary technologies, such as enhanced-efficiency fertilizers, controlled-release fertilizers, nitrification inhibitors, and crop varieties or rotations with biological nitrification inhibition (BNI) or biological*

	denitrification inhibition (BDI) capacity[77]. Implementing pollution control frameworks, such as the European Union's National Emissions Ceilings Directive[78], can further mitigate nitrogen-related environmental harm. When designing policies, it is critical to ensure that smallholder farmers are not adversely affected. Providing compensation mechanisms, technical support, and incentives to enhance fertilizer use efficiency is key to protecting vulnerable communities from the negative consequences of nitrogen pollution[79]."
